# Deciphering acclimation to sublethal combined and sequential abiotic stresses in *Arabidopsis thaliana*

**DOI:** 10.1093/plphys/kiae581

**Published:** 2024-10-29

**Authors:** Zhang Jiang, Ava Verhoeven, Yihong Li, Romy Geertsma, Rashmi Sasidharan, Martijn van Zanten

**Affiliations:** Plant Stress Resilience, Institute of Environmental Biology, Utrecht University, Padualaan 8, 3584CH Utrecht, The Netherlands; Plant Stress Resilience, Institute of Environmental Biology, Utrecht University, Padualaan 8, 3584CH Utrecht, The Netherlands; Plant Stress Resilience, Institute of Environmental Biology, Utrecht University, Padualaan 8, 3584CH Utrecht, The Netherlands; Plant Stress Resilience, Institute of Environmental Biology, Utrecht University, Padualaan 8, 3584CH Utrecht, The Netherlands; Plant Stress Resilience, Institute of Environmental Biology, Utrecht University, Padualaan 8, 3584CH Utrecht, The Netherlands; Plant Stress Resilience, Institute of Environmental Biology, Utrecht University, Padualaan 8, 3584CH Utrecht, The Netherlands

## Abstract

Plants are frequently exposed to environmental challenges. Responses to sublethal abiotic stress combinations are complex and often distinct from responses to individual stresses and remain poorly understood. Investigating traits and molecular factors mediating acclimation to stress combinations is essential for the development of climate change-resilient field crops. Here, we studied the morphological, physiological, and molecular responses of *Arabidopsis thaliana* to (i) co-occurring high temperature and drought and (ii) flooding followed by drought, both of which have increased in frequency due to climate change, and the individual component stresses: high temperature, drought, and flooding. A set of 15 physiological and morphological traits were assessed during single and combined stresses. By combining these comprehensive trait analyses with transcriptome characterization, we detected generally additive negative effects of simultaneous or sequential stresses on plant morphology and physiology compared with the corresponding individual stresses. Although drought had a mild effect on various growth, morphological, and physiological traits in both stress combinations, a unique transcriptome signature emerged upon combination with high temperature simultaneously, or flooding sequentially. Molecular processes identified as important for multistress resilience included plastid–nucleus communication, abscisic acid signaling, and photo-acclimation. Based on the RNA-seq data, a set of 39 genes were identified as potential multistress response regulators. Mutants were tested to validate the contribution of these genes to plant survival and phenotypic acclimation under combined stress. We confirmed the involvement of several genes. Among the identified factors were EARLY FLOWERING 6 (ELF6) and ARABIDOPSIS TÓXICOS EN LEVADURA 80 (ATL80), with substantial effects on plant growth, leaf development, and plant survival (wilting) during high-temperature drought and postsubmergence drought, respectively.

## Teaser

Plants exposed to simultaneous or sequential abiotic stresses display unique trait combinations and transcriptome signatures that emerge only when these abiotic stresses are combined.

## Introduction

Increased occurrences of abiotic stresses such as heat, drought, and flooding due to climate change are negatively impacting crop productivity (Shabbir et al. 2022; Kopecká et al. 2023) and thereby food security (Masson-Delmotte et al. 2021; Cushman et al. 2022; Kumar et al. 2022). Although the effect of such abiotic stresses on plant functioning has been extensively studied (Larkindale et al. 2005; Harb et al. 2010; van Veen et al. 2016), the focus has been predominantly on single stressors, often involving abrupt transfer of plants into harsh stressful conditions. In natural or agricultural settings, abiotic stresses seldom happen in isolation and usually occur at a sublethal severity (Bailey-Serres and Colmer 2014; Van Dooren et al. 2020; Zhang et al. 2020; De Smet et al. 2021) with multiple stresses coinciding simultaneously or sequentially (Mittler 2006; Suzuki et al. 2014).

Co-occurring stresses can elicit synergistic or antagonistic effects or are a blend of responses to individual stresses. Hence, responsiveness cannot be simply deduced by taking the sum of the responses of individual stresses (Rizhsky et al. 2004; Mittler 2006; Suzuki et al. 2014; Balfagón et al. 2019; Zandalinas et al. 2020a, 2020b; Rivero et al. 2022). For instance, high temperature induces stomatal opening to allow leaf cooling through transpiration, while under drought stomatal conductance is reduced to prevent unnecessary water loss. However, when high temperature and drought occur concurrently a signaling conflict occurs and the stomata tend to be closed (Rizhsky et al. 2004; Mittler and Blumwald 2010; Zandalinas et al. 2016a, 2016b).

The precise acclimation strategy fitting a given stress combination is determined by various (sometimes limiting) parameters, such as plant genotype, developmental age, duration, and severity of stresses (Zhou et al. 2017; Sinha et al. 2021; Zandalinas et al. 2021a, 2016b; Rivero et al. 2022; Zandalinas and Mittler 2022). Additionally, the sequence of events plays a pivotal role (Banti et al. 2008; Kong and Henry 2016). For example, soil salinity pretreatment can alleviate effects of subsequent low-temperature damage in tomato plants, as salt stress-triggered signal cascades can activate downstream overlapping signal transduction pathways (e.g. reactive oxygen species, abscisic acid [ABA], and low-temperature signaling) that enhance photosynthetic acclimation under low temperatures (Yang et al. 2022).

Despite its importance, multistress acclimation mechanisms remain poorly understood (Zandalinas et al. 2020a, 2020b; Rivero et al. 2022; Zandalinas and Mittler 2022). Here, we characterized the effects of 2 stress combinations typical of climate change in *Arabidopsis thaliana.* These were: (i) combined high temperature and drought and (ii) submergence followed by drought, and their corresponding individual stresses. A comprehensive assessment of the impact of these stress combinations on plant morphology, physiology, and transcriptome revealed different, in general additive negative, effects on plant growth, development, and physiology compared with the corresponding individual stresses. The unique phenotypic characteristics corresponded with distinct transcriptomic signatures involving synchronous regulation of multiple generic processes such as plastid–nucleus communication (retrograde signaling), ABA signaling, and photo-acclimation.

A reverse genetics approach was used to validate the contribution of a set of 44 candidate genes to multistress acclimation responses. Effects were confirmed for several factors, including EARLY FLOWERING 6 (ELF6) and ARABIDOPSIS TÓXICOS EN LEVADURA 80 (ATL80) that impact on specific subsets of traits related to growth, development, and water status maintenance during high temperature/drought or submergence/drought combinations. Our results reveal that acclimation to different sublethal combinatorial stresses is likely coordinated by distinct regulatory genes acting as either positive or negative regulators of multistress acclimation traits.

## Results

### Combined and sequential abiotic stresses elicit predominantly additive negative responses

**Figure 1.**
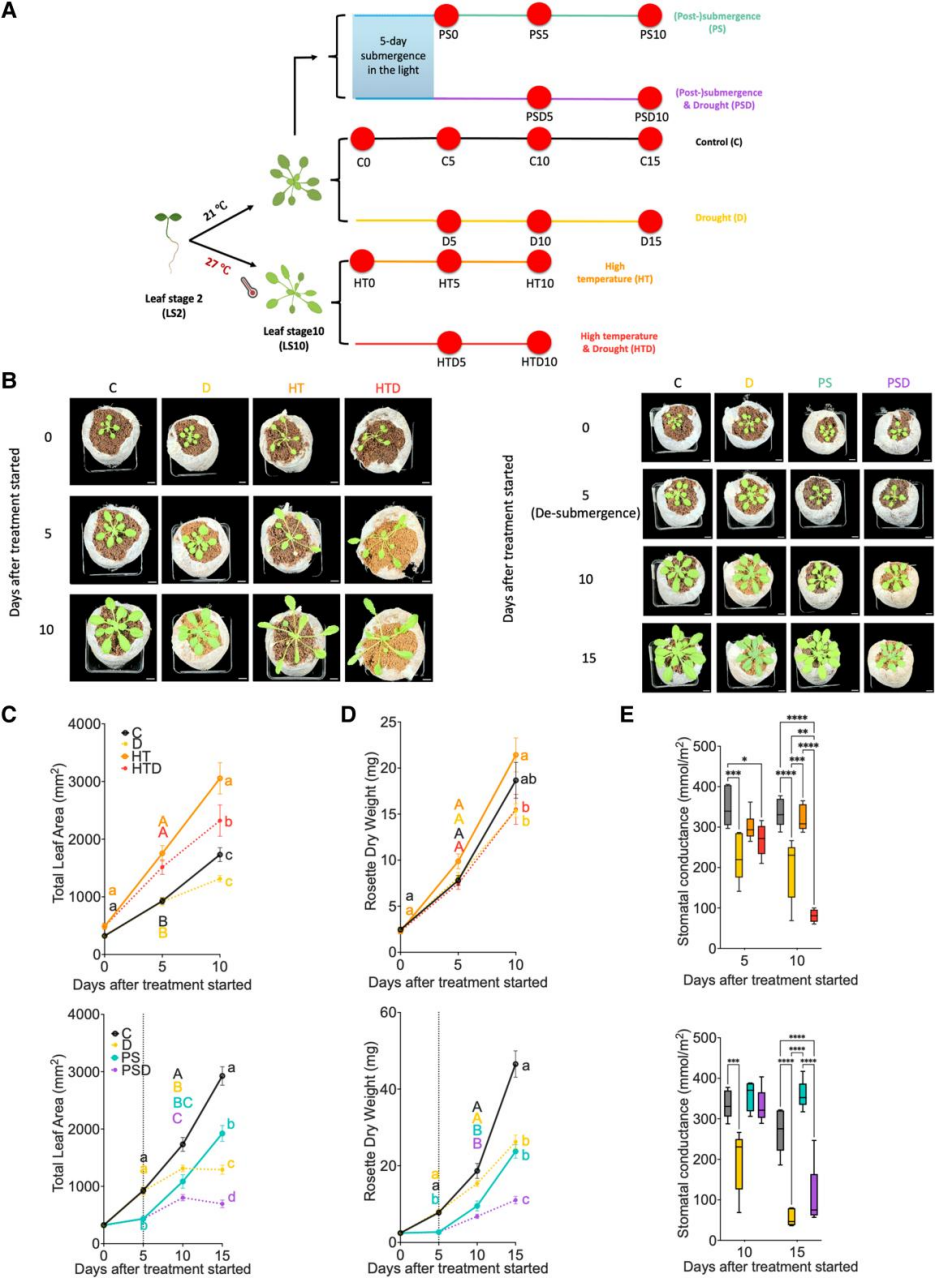
Effects of combined and sequential sublethal stresses and corresponding single stresses on phenotypic traits. **A)** Experimental setup. Harvest moments and measurements (indicated by dots) were at 0, 5, 10, and 15 d **(C** and **D)** after the drought (**D**, combined HTD and sequential PSD) treatment started at average %SWC of 100%, 50% to 65%, 30% to 40%, and ∼20%, respectively (Supplementary Fig. S1, A and B). For C, HT, and PS, the %SWC were maintained at the saturated level (95% to 100%) across all timepoints. **B)** Representative images of plants on Jiffy 7c coconut pellet growth substrate, subjected to combined (left) and sequential (right) stresses and the corresponding single stresses at 0, 5, 10, and 15 d, counted from the moment that the treatments started. Images were digitally extracted for comparison. Scale bars indicate 1 cm. **C–E)** Total leaf area of the whole rosette (**C**, *n* = 14 to 20), rosette dry weight (**D**, *n* = 15 to 18), and stomatal conductance of young leaves (**E**, *n* = 5 to 6) of plants subjected to combined (upper row) and sequential (lower row) stresses and the relevant single stresses at the corresponding harvesting timepoints. Error bars indicate means ± SEM. Letters denote significant differences between different treatments at the same timepoints (*P* < 0.05, 2-way ANOVA with Tukey's post hoc test). The dashed vertical lines indicate the moment plants were de-submerged. Boxes indicate boundaries of the second and third quartiles (Q) of the data distribution. Black horizontal bars indicate median and whiskers Q1 and Q4 values within 1.5 times the interquartile range. Asterisks represent significant differences (*P < 0.05, **P < 0.01, ***P < 0.001, ****P < 0.001, 2-way ANOVA with Tukey's post hoc test). C, control (black lines and symbols); D, drought (yellow); HT, high temperature (orange); HTD, high temperature and drought (red); S/PS, 5 d submergence/(post)submergence and recovery (blue); PSD, (post)submergence and drought (purple). Panel **A** is in part created using www.biorender.com.

*Arabidopsis* Columbia-0 (Col-0) plants were subjected to 2 different stress combinations upon reaching the 10-leaf stage (LS10): (i) high ambient temperature (27 °C) combined with progressive drought (HTD) and (ii) 5 d of submergence, sequentially followed by progressive drought after de-submergence (PSD; 21 °C) (Fig. 1A), control conditions (C; 21 °C), and corresponding single stresses: high temperature (HT; 27 °C) and submergence (S; 21 °C), whereafter plants were well watered during the postsubmergence phase (PS) (Fig. 1A). Drought (D) was imposed progressively by withholding watering. After 5 d, the average soil water content (%SWC) dropped to 50% to 65% field water capacity (=100% saturation) and to 30% to 40% after 10 d (Supplementary Fig. S1, A and B). Plants subjected to combined HTD and sequential PSD displayed earlier wilting than when D was imposed in isolation (Supplementary Fig. S2, A to C) and produced fewer new leaves than those subjected to the corresponding single stresses (Supplementary Fig. S2, D and E). Drought-treated plants (D and PSD) showed increased chlorophyll content compared with the relative controls (C and PS) (Supplementary Figs. S3 and S4). Based on these responses, we focused on the 0, 5, 10, and 15 d following stress initiation timepoints in the remainder of this study (Fig. 1A).

#### High temperature and drought

D did not substantially hamper rosette growth nor dry weight accumulation, but triggered primary root lengthening (Fig. 1, B–D; Supplementary Fig. S5D). Plants in HT showed the expected typical thermomorphogenic phenotype (Quint et al. 2016), including elongated petioles and hyponastic leaves (Fig. 1B; Supplementary Figs. S5, A–C and S6). Combined HTD treatment resulted in smaller rosettes and shorter petioles and leaf blades compared with plants exposed to HT, but rosette size, petiole length, and leaf blade length under HTD exceeded the values observed under D (Fig. 1C; Supplementary Figs. S5, B and C and S6). Plants grown in HT or D did not differ in dry weight compared with plants in C (Fig. 1D). However, HTD resulted in significant dry weight reduction after 10 d compared with HT, but not C (Fig. 1D). Primary root length was significantly promoted under HTD compared with the individual stresses (Supplementary Fig. S5D). Together, this indicates that during the subjection to HTD, D, and HT stresses interact.

Plants contained relatively more water under high-temperature conditions (HT and combined HTD) than at control temperatures (C and D) (Supplementary Fig. S7A). Moreover, the differences in leaf surface temperature between “control” and drought-treated plants at 27 °C (HT and HTD) were greater than at 21 °C (C and D) (Supplementary Fig. S7, B and C). Together with an accelerated drought-induced temperature increase at 27 °C compared with 21 °C (Supplementary Fig. S7, B and C), these results indicate that the drought effect on leaf temperature was more pronounced when combined with high temperature. However, stomatal conductance of plants in C and HT was not significantly different, whereas C and combined HTD did differ significantly, with HTD having lower stomatal conductance (Fig. 1E). D caused, as expected, a reduction in relative water content corresponding with a significant decline in stomatal conductance compared with C (Fig. 1E; Supplementary Fig. S7A).

No differences in malondialdehyde (MDA) content were observed across treatments up to Day 10 (Supplementary Fig. S7D), suggesting that no substantial oxidative stress occurs. However, enhanced ion leakage in HT-treated plants (Supplementary Fig. S7E) points to membrane damage at high temperatures (Nielsen et al. 1997; Sharma et al. 2012). The addition of 10-d drought (HTD) tempered this effect, while no effect was observed when D was applied in isolation (Supplementary Fig. S7E).

#### Submergence and drought

Five-d complete submergence (S) led to severely inhibited rosette growth compared with nonsubmerged plants (Fig. 1, B–D; Supplementary Figs. S8 to S10). During postsubmergence plants could recover if irrigation was applied (PS) (Fig. 1, B–D; Supplementary Figs. S8 to S10). D had a significant negative effect on total rosette area and dry weight, compared with C after 10 d (Fig. 1, C and D). When D was imposed following de-submergence (PSD), total rosette area and dry weight accumulation were further reduced compared with PS (Fig. 1, C and D; Supplementary Fig. S10, A and E). In contrast to D, S restricted primary root lengthening, whereas root elongation was enhanced under PSD (Supplementary Figs. S8D and S10F).

D negatively affected relative water content after 15 d (Supplementary Fig. S11A), which was enhanced under sequential PSD (Supplementary Figs. S11A and S12A). Both D and PSD treatments resulted in a reduction of stomatal conductance (Fig. 1E; Supplementary Fig. S12B). Accordingly, leaf temperature was raised under these conditions (Supplementary Fig. S11, B and C).

Drought increased MDA accumulation, especially when combined with prior submergence (PSD) (Supplementary Figs. S11D and S12C). Yet, no clear effect of PSD, D, or PS was observed on ion leakage (Supplementary Figs. S11E and S12D).

### Transcriptomic responses elicited by combined and sequential stresses

Next, a comparative RNA-sequencing approach was taken using young leaves sampled 0, 5, and 10 d after stress initiation (Fig. 1A) to uncover molecular processes underlying responses to combined and sequential stresses. In total, 58 samples, distributed over 15 different harvesting timepoints were sequenced, resulting in 3,628,334,664 detected reads (Supplementary Fig. S13A). On average, 72% of the mapped reads aligned to the Arabidopsis transcriptome (5.4 to 61.8 million reads per sample). For approximately one-third of sequenced samples <50% of the reads mapped to the Arabidopsis transcriptome (Supplementary Fig. S13A). 98.2% of the unmapped reads were identified as RNA segments of *Arabidopsis Latent Virus 1* (ArLV1 RNA1 and RNA2; GenBank accession nos. MH899120.1 and MH899121.1, respectively) (Verhoeven et al. 2023). Although this virus does not evoke visible symptoms in plants (Verhoeven et al. 2023), viral infections could impact the host transcriptome (Shates et al. 2019). We therefore compared samples with reads that mapped more than 50% with those mapping less than 50% to the Arabidopsis transcriptome on Day 5 C treatment (Supplementary Fig. S13B). No differentially expressed genes between both fractions were detected, implying that ArLV1 does not substantially impact the plant transcriptome.

Principal component analysis (PCA) revealed significant changes across time (PCO1, explaining 29.88% of observed variation) and treatments (separated over PCO1 and PCO2) (Supplementary Fig. S14A). As expected, individual biological replicates of the same timepoint and stress treatment clustered together (Supplementary Figs. S14 and S15). Superimposing the fraction of reads mapping to the Arabidopsis genome per sample on the PCA revealed no clear pattern (Supplementary Fig. S14B), confirming that ArLV1 contamination did not substantially affect the transcriptome. We therefore decided to continue processing our data using all samples except 2 (Day 5 control) of which the mapping percentages to Arabidopsis transcriptome were below 11% (Supplementary Fig. S13).

### High temperature and submergence alter transcriptome responses to drought

#### High temperature and drought

**Figure 2.**
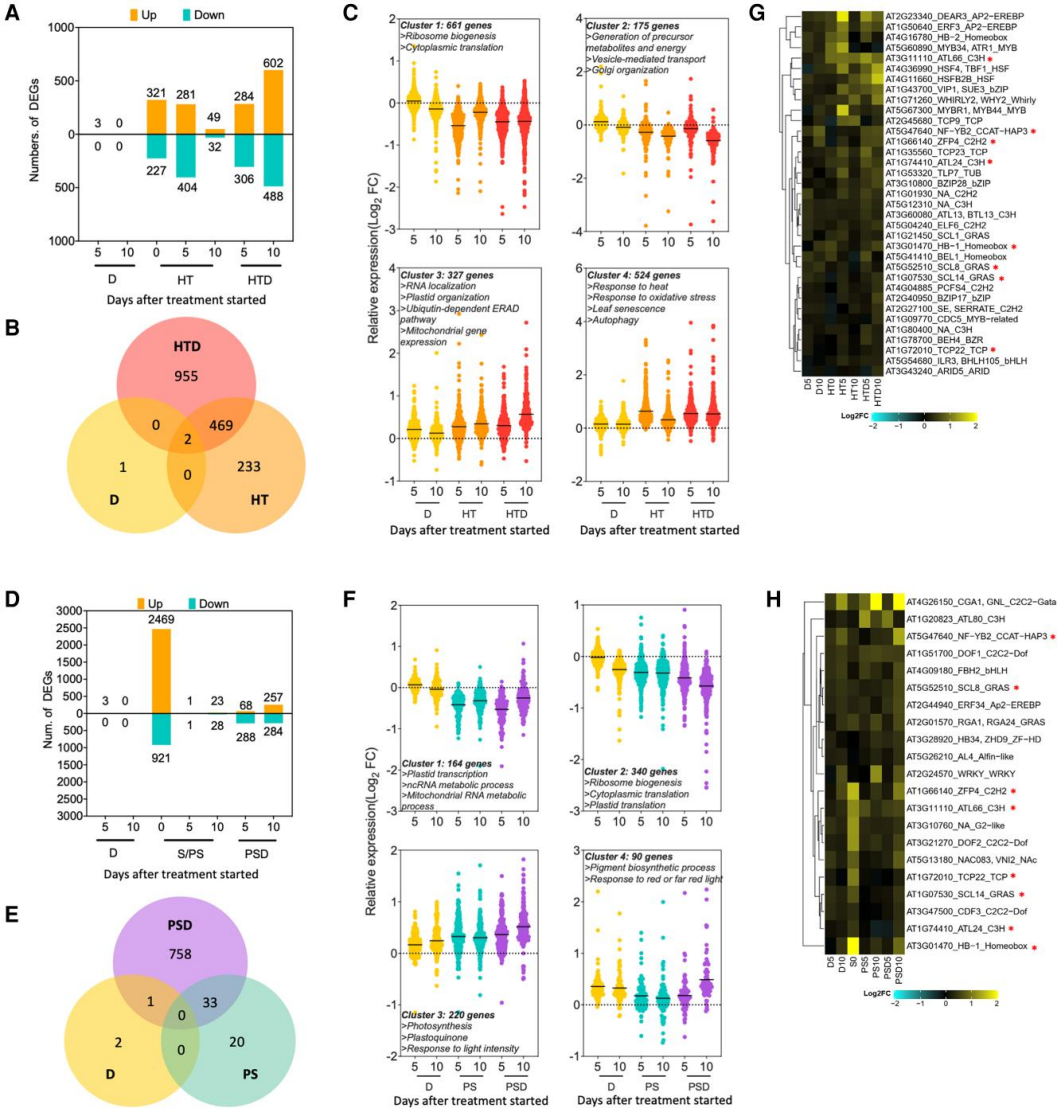
Effect of combined and sequential stresses and corresponding single stresses on the transcriptome. **A)** and **D)** Number of upregulated (orange) and downregulated (green) regulated DEGs (*P* < 0.05) at 0 (the pregrowth effect of HT before the drought treatment started), 5, and 10 d after the treatments [D] started (note HT was prolonged), compared with control (C) conditions. **B)** and **E)** Venn diagrams showing the number of DEGs commonly or differently detected in the combined (HTD), sequential (PSD), and single (D, HT, and PS) stresses. **C)** and **F)**  *k-means* clustering presenting expression patterns of all identified DEGs at timepoints 5 and 10 d during combined or sequential stresses and the corresponding individual stresses. Violin plots indicate the distributions of relative expression (Log_2_FC) of identified DEGs. Note that in each treatment all genes are indicated that are significantly regulated in at least one or more of the treatments. Mean values of each violin plot are indicated by a solid black line. Key enriched biological processes identified by GO analysis and number of genes assigned to each cluster are listed above or below the graphs. **G)** and **H)** Hierarchically clustered heatmaps showing relative Log_2_FC levels of TFs (Supplementary Tables S3 and S4) identified among the upregulated DEGs in the HTD and PSD datasets. For each identified TF, the AGI gene locus ID, the commonly used abbreviation (if available) and TF family the factor belonged to, are listed. Color scales indicate Log_2_FC values (relative to control [C] conditions). Asterisks indicate overlapping TFs between HTD and PSD. For treatment abbreviations and used colors, see legend of Fig. 1.

In response to D, only 3 differentially expressed genes were detected, despite using a relatively lenient threshold (DEGs FDR < 0.05); *GUARD-CELL-ENRICHED GDSL LIPASE 3* (*GGL3*), *PHOSPHOFRUCTOKINASE 1* (*PFK1*), and *AT5G16990* (Fig. 2A). This is likely due to the mild progressive drought used here. However, plants in our treatment did experience drought as evidenced by typical physiological responses such as an elongated primary root (Supplementary Fig. S5D) and reduced stomatal conductance (Fig. 1E). Under combined HTD, the drought effect became more apparent (590 and 1,090 DEGs after 5 and 10 d of HTD, respectively) (Fig. 2A). Moreover, combined HTD resulted in 955 unique DEGs that were not detected under D or HT (Fig. 2B). Thus, combined stress evokes a unique transcriptomic response and has greater impact than individual stresses. HT and HTD shared 469 DEGs, suggesting that HT has a stronger effect on the transcriptome than D in the combined stress (HTD) situation (Fig. 2B). Only 2 DEGs, *PFK1* and *AT5G16990*, were commonly regulated by D, HT, and HTD (Fig. 2B), suggesting that these genes might be involved in early stress responses in young leaves.

*K-means* clustering of the 1,687 detected DEGs (Days 5 and 10) across individual and combined stress treatments revealed 4 groups with distinct expression patterns (Fig. 2C). Clusters 1 and 2 consisted of DEGs downregulated under HT and combined HTD conditions and unchanged under D. Gene Ontology (GO) annotation revealed that these clusters were dominated by genes associated with ribosome activities and protein processing (Fig. 2C; Supplementary Table S1). DEGs that were upregulated in HT and HTD were in Clusters 3 and 4 (Fig. 2C). These DEGs are associated with RNA processing (particularly alternative splicing), organelle activities including ubiquitin-depended endoplasmic reticulum–associated protein degradation pathway, mitochondrial activities, and stress responsiveness such as response to heat, starvation, or oxidative stress and leaf senescence (Fig. 2C; Supplementary Table S1).

#### Submergence and drought

To characterize the effects of submergence followed by drought (PSD), the following comparisons (Log_2_FC in gene expression) were made: (i) 5-d submergence (S) compared with presubmergence (submergence effect), (ii) 5 or 10 d of D compared with C (drought effect), and (iii) 5- or 10-d PS or PSD compared with C, respectively, to determine effects of S followed by irrigation (PS) or drought (PSD). This approach ensured comparisons between samples at similar developmental stages.

Five-d submergence resulted in 3,390 DEGs (2,469 up- and 921 downregulated) (Fig. 2D), reflecting considerable transcriptome reconfiguration. Following de-submergence (PS), only a limited number of DEGs were detected after 5 and 10 d (2 and 51 DEGs, respectively; Fig. 2D). Upregulation of several core hypoxia-responsive genes (Mustroph et al. 2009) during 5-d S pointed to occurrence of tissue hypoxia (Supplementary Fig. S16). As expected, the upregulation was reversed by re-oxygenation following de-submergence (PS and PSD at Days 5 and 10) (Supplementary Fig. S16). D during de-submergence (PSD) resulted in 356 and 541 DEGs at 5 and 10 d of PSD, respectively (Fig. 2D). Like combined HTD, sequential PSD also led to a unique transcriptomic signature, as the majority of DEGs identified after PSD treatment (758 DEGs) were not affected by PS nor D (Fig. 2E). Only 33 DEGs were shared between PSD and PS (Supplementary Fig. S17).

*k-means* clustering including all 814 DEGs (Days 5 and 10 across D, PS, and PSD treatments; Fig. 2F) revealed 4 clusters. Two clusters contained genes downregulated by PS and PSD (Clusters 1 and 2) and 2 contained genes upregulated in these conditions (Clusters 3 and 4) (Fig. 2F; Supplementary Table S2). GO analysis revealed that Clusters 1 and 2 were dominated by genes associated with nucleic acid, ribosome, and translation-related processes. Several of the downregulated GO terms in Clusters 1 and 2 were associated with plastid functions, indicating impeded chloroplast activities during postsubmergence (PS and PSD). In Cluster 3, photosynthesis-related GO terms were significantly enriched, while in Cluster 4 only 2 GO categories were enriched (Fig. 2F; Supplementary Table S2). Of note, *CYTOKININ-RESPONSIVE GATA FACTOR 1* (*CGA1*/*GNL*) was shared between these 2 GO groups.

### Transcription factors mediating acclimation to combined and sequential stresses

In total, 48 different transcription factors (TFs) from 23 TF families were upregulated by combined HTD (35) or sequential PSD (21), with 8 TFs overlapping between the 2 groups (Fig. 2, G and H; Supplementary Fig. S18). Only 7 out of 35 HTD-upregulated TFs were also upregulated under HT (Supplementary Table S3), and only 2 out of 21 PSD-upregulated TFs, were induced under 5- or 10-d PS (Supplementary Table S4). This underscores the unique response that is evoked by combined stress application. Considering sequential PSD, of the 19 uniquely induced TFs, 12 were significantly upregulated by 5-d S (Supplementary Table S4). This points to a large effect of prior submergence on the sequential stress transcriptome signature.

Gene regulatory networks (GRNs) were constructed with DEGs from the combined HTD and sequential PSD-upregulated clusters (Clusters 3 and 4) to identify putative regulatory nodes using the TF2network tool (Kulkarni et al. 2018) (Supplementary Fig. S19, A and B). This tool integrates the information of both of protein–DNA interactions and co-expression that are experimentally confirmed or computationally predicted in Arabidopsis. The obtained combined HTD network consisted of 12,913 interactions between 847 regulators (nodes). Only 4,169 interactions between 340 regulators were formed in the sequential PSD network (Supplementary Fig. S19, A and B). To identify putative “master regulators,” 11 nodes with the highest number of connections with the other regulators in the same GRN were selected. Four of these were shared between the HTD and PSD networks: *G-BOX BINDING FACTOR 3* (*GBF3*), *ABSCISIC ACID RESPONSIVE ELEMENT-BINDING FACTOR 1* (*ABF1*), *ABA INSENTITIVE 5* (*ABI5*), and *NUCLEAR FACTOR Y, Subunit B2* (*NF-YB2*) (Supplementary Fig. S19, table embedded in figure). Note that *THYLAKOID LUMEN PROTEIN* (*TLP18.3*) ranked 12th in the degree analysis and is associated with chloroplast function (Wu et al. 2011; Järvi et al. 2016). *GENOMES UNCOUPLED 4* (*GUN4*) and *AT1G66130* had the same number of nodes in GRN; therefore, in total 11 overrepresented genes for HTD and PSD are indicated in Supplementary Fig. S19.

### Validation of identified candidate genes by mutant analysis

Next, a reverse genetics approach was taken to validate the effects of identified factors on multistress acclimation (Supplementary Table S5). Because the validation was based on assessing mutants, only TFs that were upregulated during either combined HTD or sequential PSD, supplemented with some of the overrepresented genes from the GRNs, were considered in this study. Hence, the downregulated DEGs from Clusters 1 and 2 under HTD (836 DEGs) and PSD (504 DEGs) (Fig. 2) were not considered here. In total, 41 confirmed homozygous T-DNA insertion mutants, covering in total 39 candidate genes (28 for HTD and 19 for PSD), were obtained (Supplementary Tables S6 and S7).

#### High temperature and drought

**Figure 3.**
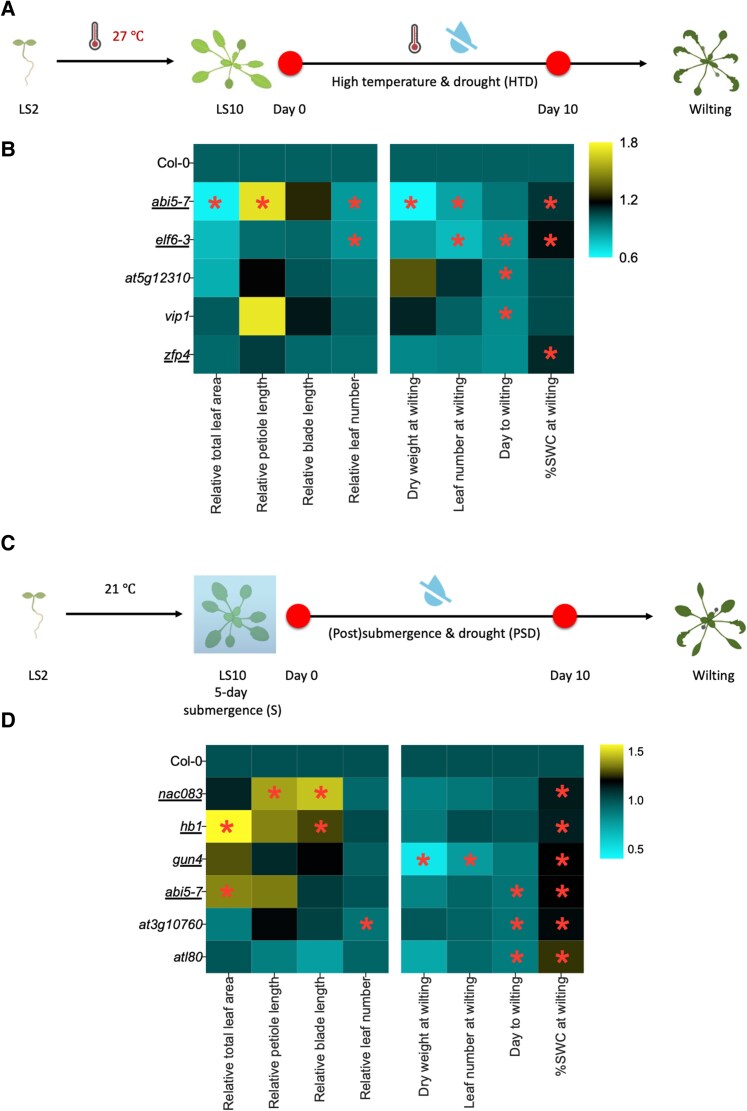
Plant growth, development, and wilting traits of selected mutants under combined or sequential stresses. **A)** and **C)** Experimental schemes for the used reverse genetic analyses. Two-leaf stage (LS2) Arabidopsis mutants and corresponding WT plants were pregrown at 27 °C (HTD; **A)** or 21 °C (PSD; **C)** until they reached the 10-leaf stage (LS10). Subsequently, the plants were subjected to 10 d of combined high-temperature and drought conditions (HTD), or 5-d submergence in the light, followed by 10-d drought treatment (PSD) and thereafter were both left unwatered until wilting occurred. Measurements of phenotypic traits were taken at Days 0 and 10 and at the moment of wilting (i.e. timepoint differed per plant). **B)** and **D)** Heatmaps indicating phenotypic traits of Arabidopsis mutants with either significantly advanced wilting time and/or high %SWCW, relative to the corresponding WT plants (Col-0) during the exposure to combined HTD **(B)** or sequential PSD **(D)**. The relative values of total leaf area, petiole/blade length, and leaf number were calculated by normalizing the data obtained at Day 10 by that of Day 0 (left 4 columns in each panel). Dry weight and leaf number at wilting, Days to wilting and %SWC at wilting are indicated in the right 4 columns per panel. The abbreviated names of tested mutants and the WT (Col-0) plants are indicated per row. Color scales indicate relative values of measured traits (relative to the corresponding WT Col-0 plants). Yellow and cyan indicate positive and negative effect, respectively, compared with Col-0. Underlined mutants indicate confirmed knockout of the corresponding genes based on previous studies (Supplementary Tables S6 and S7). Asterisks represent significant differences between mutants and WT plants for the particular trait (Supplementary Tables S8 and S9) (*P* < 0.05, unpaired *t*-test). *n* = 8 to 12. Panels **A** and **C** are in part created using www.biorender.com.

Traits indicative of plant growth and development were measured at 0 and 10 d after stress initiation. Subsequently, the stress treatment was extended until the plants showed wilting symptoms (Fig. 3A). Of the 29 obtained homozygous mutants with a putative effect on HTD traits (Fig. 3A; Supplementary Table S6), *aba insensitive 5* (*abi5-7*), *early flowering 6* (*elf6-3*), *at5g12310*, *VIRE2-interacting protein 1 (vip1)*, and *zinc finger protein 4* (*zfp4*) displayed early wilting phenotypes (Fig. 3B; Supplementary Table S8). As expected, the ABA signaling mutant *abi5-7* (Dekkers et al. 2016; Bi et al. 2017) showed substantially deviating phenotypes under combined HTD compared with the wild type (WT) in addition to clear wilting phenotypes (Fig. 3B; Supplementary Table S8). This points to a pivotal role for ABA signaling in modulating plant growth and development under HTD. The *elf6-3* mutant showed accelerated wilting (Fig. 3B; Supplementary Table S8) and a considerable delay in leaf initiation rate.

#### Submergence and drought

Of the 20 obtained homozygous mutants tested (Fig. 3C; Supplementary Table S7), 6 (*NAC domain containing protein 83* (*nac083*), *homeobox1* (*hb1*), *gun4*, *at3g10760*, *abi5-7*, and *arabidopsis tóxicos en levadura 80* (*atl80*)) exhibited early wilting phenotypes under sequential PSD (Fig. 3D; Supplementary Table S9). The *nac083*, *hb1*, and *abi5-7* mutants exhibited a significant increase in total leaf area and/or leaf length (Fig. 3D; Supplementary Table S9). Both *gun4* and *at3g10760* showed reduced leaf initiation rates. Notably, *gun4* weighed only ∼50% of the (dry) weight at the moment of wilting compared with WT (Supplementary Table S9). Despite wilting at a high %SWC level, *atl80* mutant phenotypic traits were comparable to WT (Fig. 3D; Supplementary Table S9).

**Figure 4.**
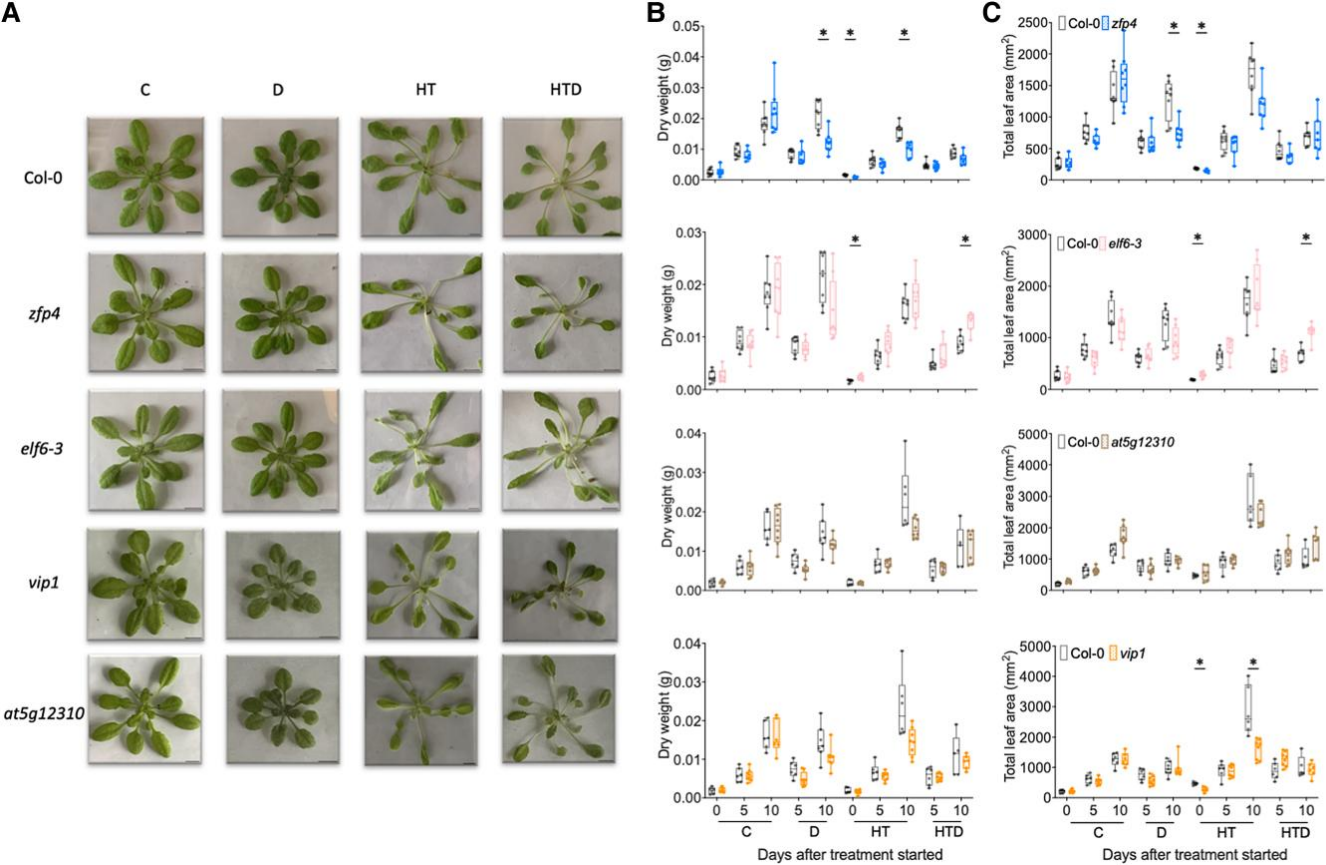
Effect of combined and individual stresses on phenotypic traits of selected Arabidopsis mutants. **A)** Representative images of whole rosettes of selected mutants and corresponding WT plants (Col-0) grown on Jiffy 7c coconut pellet substrate, subjected to single and combined stresses at 10 d after the treatments initiated. Scale bars indicate 1 cm. Rosette dry weight **(B)** and total leaf area **(C)** of Arabidopsis mutants (*zfp4* [blue], *elf6-3* [pink], *at5g12310* [brown], and *vip1* [orange]) and the WT plants (Col-0; gray). For plants that wilted before the harvesting timepoints, traits were measured at the day of wilting onset. Boxes indicate boundaries of the second and third quartiles (Q) of the data distribution. Horizontal bars indicate median and whiskers Q1 and Q4 values within 1.5 times the interquartile range. Asterisks represent significant differences between the mutant and the corresponding WT plants within the same timepoint (*P* < 0.05, multiple unpaired *t*-test with Holm–Šídák correction). Numbers indicate days after treatments started. For treatment abbreviations and used colors, see legend of Fig. 1. *n* = 5 to 10.

**Figure 5.**
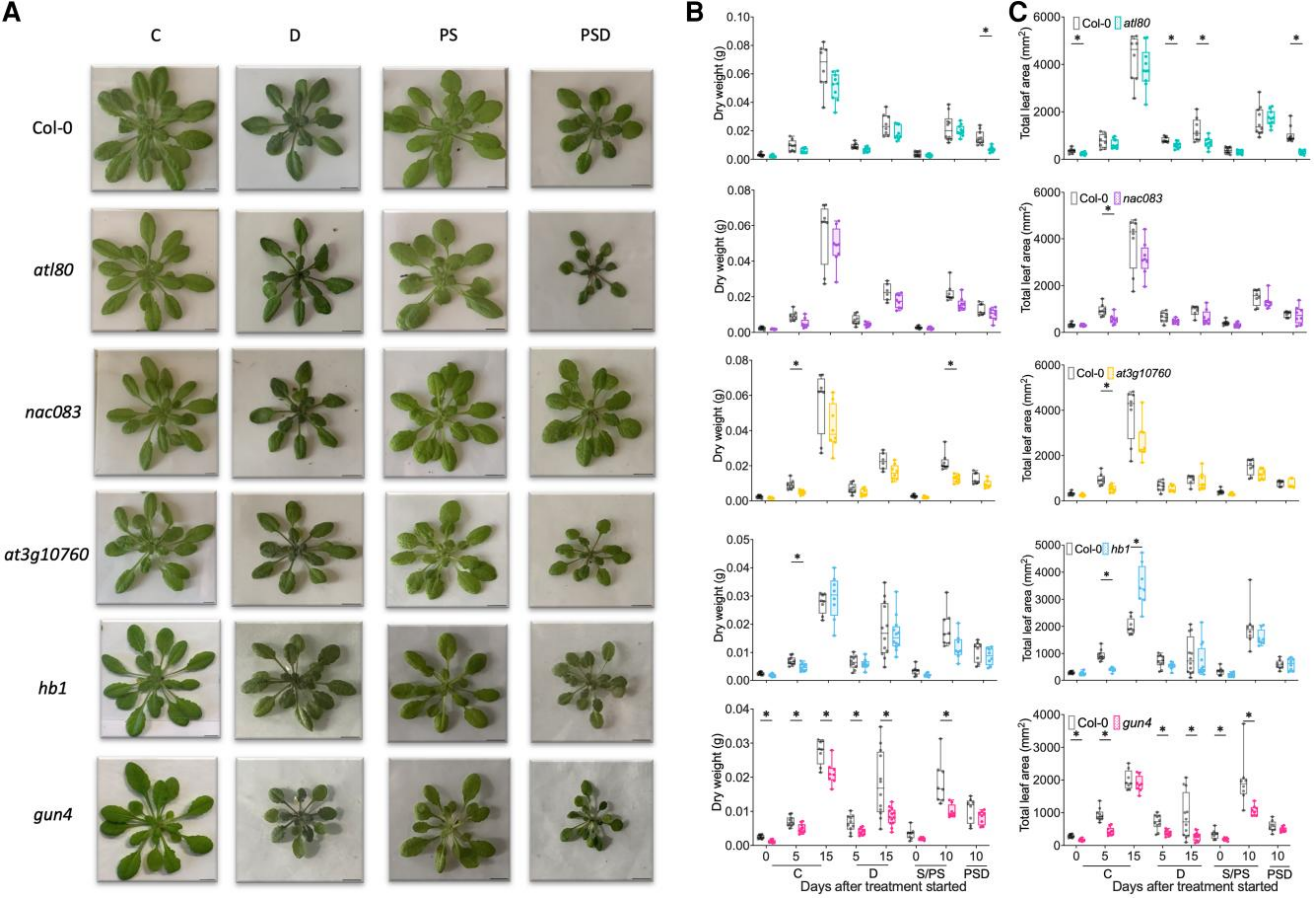
Effect of sequential and individual stresses on phenotypic traits of selected Arabidopsis mutants. **A)** Representative images of whole rosettes of selected mutants and the corresponding WT plants (Col-0) grown on Jiffy 7c coconut pellet substrate, subjected to single and sequential stresses 15 d after the treatments started. Scale bars indicate 1 cm. Rosette dry weight **(B)** and total leaf area **(C)** of Arabidopsis mutants (*atl80* [aqua], *nac083* [purple], *at3g10760* [yellow], *hb1* [azure], and *gun4* [magenta]) and the WT plants (Col-0; gray). For plants that wilted before the harvesting timepoints, the traits were measured on the day of wilting onset. Boxes indicate boundaries of the second and third quartiles (Q) of the data distribution. Horizontal bars indicate median and whiskers Q1 and Q4 values within 1.5 times the interquartile range. Asterisks represent significant differences between the mutant and the corresponding WT plants within the same timepoint (*P* < 0.05, multiple unpaired *t*-test with Holm–Šídák correction). Numbers indicate days after treatment started. For treatment abbreviations and used colors, see legend of Fig. 1. *n* = 6 to 13.

For mutants exhibiting early wilting during combined HTD (*elf6-3*, *at5g12310*, *vip1*, and *zfp4*) or PSD (*atl80, nac083, at3g10760, hb1, gun4*), experiments were extended with inclusion of the corresponding individual stresses (Figs. 4 and 5; Supplementary Figs. S20 to S23). The *abi5-7* mutant was omitted as ABA signaling is already studied in great detail in plant acclimation to diverse stresses (Skubacz et al. 2016; Wang et al. 2021).

#### Validation of high-temperature and drought mutants

Compared with WT, the *elf6-3* mutant had higher dry weight and larger rosette area under combined HTD treatment (Fig. 4, B and C). This mutant also displayed enhanced dry weight and had a larger rosette area at 0 d (pregrowth) at HT, but neither at later HT timepoints nor under C and D treatments (Fig. 4, B and C). This indicates that ELF6 is a negative regulator of plant growth and rosette expansion under combined HTD. The *zfp4* mutant showed reduced dry weight and total leaf area under 0-d HT and 10-d D, whereas *vip1* only showed a smaller total leaf area than the WT under 0- and 10-d HT (Fig. 4, B and C). This suggests that both ZFP4 and VIP1 mediate plant growth during D and/or HT but not HTD.

Under combined HTD treatment, all mutants with accelerated wilting displayed a significant decrease in chlorophyll content (Supplementary Fig. S21A). In contrast, both *vip1* and *at5g12310* displayed higher chlorophyll content under 10-d D (Supplementary Fig. S21A). No significant differences in chlorophyll abundance were observed between mutants and WT during HT single treatment (Supplementary Fig. S21A). Under HT and HTD, stomatal conductance of all mutants was comparable to WT (Supplementary Fig. S21B). Notably, 5-d D significantly reduced stomatal conductance in both *vip1* and *at5g12310* mutants compared with WT (Supplementary Fig. S21B).

#### Validation of postsubmergence and drought mutants

The *atl80* mutant exhibited decreased dry weight and total leaf area compared with the WT when subjected to sequential PSD and developed smaller rosettes and shorter leaves during C and D treatment (Fig. 5, A–C; Supplementary Fig. S22, A and B). Both *nac083* and *hb1* mutants had similar dry weights and total leaf area as the WT during individual (D, PS) and sequential stresses (PSD) (Fig. 5, B and C). However, prolonged PS treatment resulted in decreased blade length and leaf number in *nac083* (Supplementary Fig. S22, B and C). As reflected by reduced dry weight accumulation, blade length, and leaf number during 10-d PS, the *at3g10760* mutant was unable to recover to the same extent as WT after submergence (Fig. 5B; Supplementary Fig. S22, B and C). The *gun4* mutant appeared highly sensitive to D and PS, as dry weight, total leaf area, leaf length, and leaf initiation rate were significantly reduced (Fig. 5, B and C; Supplementary Fig. S22, B and C). However, *gun4* also exhibited a significant reduction in these traits under C (Fig. 5, B and C; Supplementary Fig. S22, B and C). Chlorophyll content and stomatal conductance remained unchanged for most mutants (Supplementary Fig. S23, A and B), except *gun4,* which had higher chlorophyll and stomatal conductance under 10-d C (Supplementary Fig. S23, A and B).

### Principal component analysis reveals trait correlations and validates mutant effects

**Figure 6.**
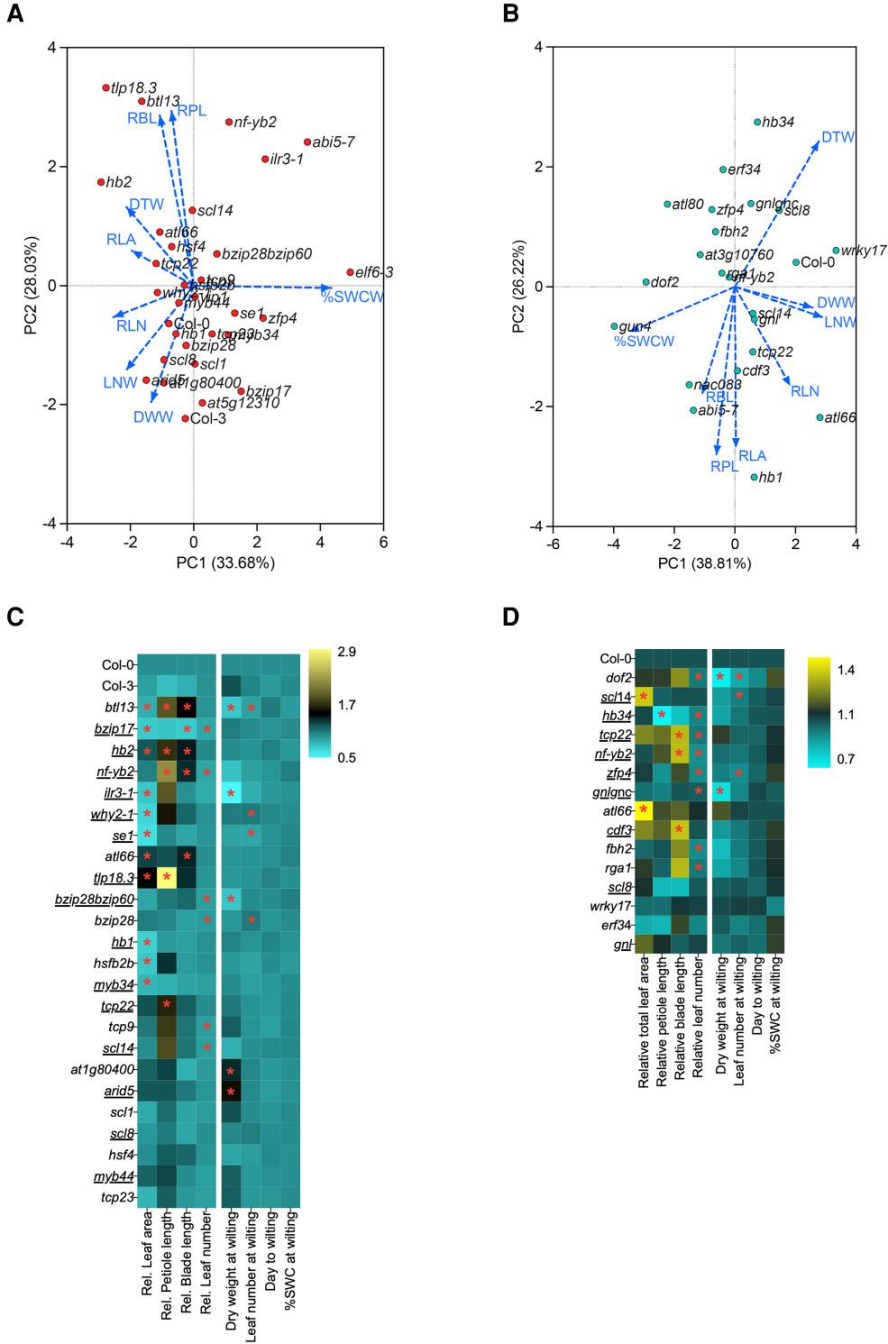
Plant growth, development, and wilting trait analyses of selected mutants under combined or sequential stresses. PCA of all measured traits of all tested mutants and corresponding WT plants subjected to combined HTD **(A)** or sequential PSD **(B)**. Visualized are the distributions of mutants and WT plants indicated by symbols and correlations between measured traits by arrows. **C)** and **D)** Heatmaps indicating phenotypic traits of mutants without early wilting phenotypes (opposed to those indicated in Figs. 3 and 4) along with the corresponding WT plants (Col-0 and Col-3) in response to combined HTD **(C)** or sequential PSD **(D)**. Relative values of total leaf area, petiole/blade length, and leaf number were calculated by normalizing the data obtained at Day 10 by that of Day 0 (left 4 columns). Dry weight and leaf number at wilting, DTW, and %SWCW are indicated in the (right 4 panels). Abbreviated names of mutants and the WT plants are indicated per row. Color scales indicate relative values of measured traits (relative to the corresponding WT plants, with Col-3 relative to Col-0); yellow and cyan indicate positive and negative effect, respectively, compared with Col-0. Underlined mutants indicate confirmed knockout mutants of the corresponding genes based on previous studies (Supplementary Tables S6 and S7). Asterisks represent significant differences between mutant and the corresponding WT plants for the particular trait (Supplementary Tables S8 and S9) (*P* < 0.05, unpaired *t*-test), *n* = 8 to 12. For treatment abbreviations and used colors, see legend of Fig. 1.

A PCA incorporating all measured traits of all mutants and WTs revealed strong correlation between leaf development traits (including relative petiole length [RPL] and relative blade length [RBL], and relative total leaf area [RLA] (Fig. 6, A and B). Likewise, relative leaf number (RLN), leaf number at wilting (LNW), and relative dry weight at wilting (DWW) were positively correlated (Fig. 6, A and B). As expected, days to wilting (DTW) and %SWC at wilting (%SWCW) negatively correlated (Fig. 6, A and B), indicating that fast-wilting mutants indeed also generally contained a high %SWC at the moment of wilting.

#### High temperature and drought

The *tlp18.3* and *bca2a zinc finger atl13* (*btl13*) mutants exhibited altered leaf lengthening traits under combined HTD treatment and displayed significantly increased (young) leaf lengthening compared with WT (Col-0 or Col-3) (Fig. 6, A and C; Supplementary Table S8). For example, *tlp18.3* recorded a RPL that was ∼3-fold higher than Col-0 (Fig. 6C; Supplementary Table S8). A similar trend was observed for *homeobox 2* (*hb2*), *nf-yb2*, *arabidopsis tóxicos en levadura 66* (*atl66*), and *teosinte branched1*/*cycloidea*/*proliferating cell factor 22* (*tcp22*) mutants, exhibiting enhanced relative petiole and/or blade elongation (RPL and RBL) under combined HTD (Fig. 6, A and C; Supplementary Table S8). The mutants *btl13*, *why2-1*, and *serrate* (*se-1*) showed reduced total leaf area and leaf number at wilting compared with the WT (Fig. 6C; Supplementary Table S8).

The basic region/leucine zipper motif (bZIP) TFs, bZIP17, and bZIP28 proteins have been implicated in heat stress responses (Kataoka et al. 2017; Gao et al. 2022). Compared with the WT, *bzip17* displayed significantly reduced total rosette area, blade lengthening, and leaf formation under HTD, while *bzip28* had reduced leaf number (Fig. 6C; Supplementary Table S8). The *bzip28 bzip60* double mutant (Samperna et al. 2021) showed a significant delay in both leaf formation and dry weight accumulation (Fig. 6C; Supplementary Table S8). *ilr3-1* mutant plants exhibited strongly enhanced hyponastic growth when grown at elevated ambient temperature (27 °C), whereas additional drought during high temperature (combined HTD) did not further alter this phenotype (Supplementary Fig. S24, A and B). Next to these observations, several mutants displayed significant changes in only one of the measured traits (Fig. 6C; Supplementary Table S8). Notably, *ilr3-1* phenotypically resembled *abi5-7* (Fig. 6, A and C).

#### Postsubmergence and drought

The *DNA binding with one finger 2* (*dof2*) mutant showed reduced leaf formation rate and dry weight accumulation under sequential PSD (Fig. 6D; Supplementary Table S9). The *scl14* mutant showed increased total leaf area, whereas leaf number at wilting was reduced (Fig. 6D; Supplementary Table S9). Interestingly, *hb34*, which displayed decreased petiole length and leaf formation rate, exhibited a typical leaf-rolling phenotype in sequential PSD, but not under C (Fig. 6D; Supplementary Fig. S25, A and B). Additionally, *tcp22* and *nf-yb2* mutants had significantly longer leaf blades but reduced leaf initiation rates (Fig. 6D; Supplementary Table S9). The *zfp4* mutant only showed a lag in leaf formation in sequential PSD (Fig. 6D; Supplementary Table S9). The single *gnl* mutant (Richter et al. 2013) had no clear phenotype (Fig. 6D, Supplementary Table S9). However, in combination with a knockout of the *GNL* homolog, *GATA NITRATE-INDUCIBLE CARBON-METABOLISM INVOLVED* (*GNC*) (*gnlgnc*) reduced dry weight and impaired leaf initiation were noted (Fig. 6D; Supplementary Table S9). Additionally, both *atl66* and *cycling dof factor 3* (*cdf3*) mutants had larger leaves (Fig. 6D; Supplementary Table S9). The *flowering bhlh 2* (*fbh2*) and *repressor of ga1-3 1* (*rga1*) mutants showed a significant reduction in RLN compared with the WT when exposed to sequential PSD (Fig. 6D; Supplementary Table S9).

## Discussion

Co-occurring abiotic stresses often have distinct effects on plants and elicit different acclimation responses compared with individual stresses (Choudhury et al. 2017; Zhang and Sonnewald 2017; Zandalinas and Mittler 2022; Nadeem et al. 2023). In addition, (combined) abiotic stresses encountered in natural or agricultural conditions are often at a gradual or sublethal severity, and hence relatively mild, compared with those reported in experimental laboratory studies (Zhang et al. 2020; Zandalinas et al. 2021b; Morales et al. 2022). Previous research has revealed that in general, multifactorial stress combinations of sublethal severity have negative impacts on plant growth and survival (Zandalinas et al. 2021b). This highlights the importance of studying plant acclimation to stress combinations at a relevant stress intensity.

The experimental system that we present in this work departed from standard protocols of abrupt stress exposures. Arabidopsis plants were exposed to sublethal single and combined/sequential stresses that typify weather events exacerbated by climate change. Our comprehensive dataset reveals a suite of unique phenotypic and molecular signatures of plant responses that emerge only when these sublethal stresses are applied sequentially or simultaneously (e.g. Figs. 1 and 2). These responses are also highly dependent on the duration and sequence of stress imposition (Guo et al. 2021). For instance, many studies have explored the combined effects of heat and drought, but with varying results. For example, a predominant drought signature was noted on plant growth and development when heat and drought were applied concurrently (Zhou et al. 2017). In addition, in wheat (*Triticum aestivum*), episodes of prolonged drought in combination with heat waves exacerbate biomass reduction and loss of grain yield, when compared with individually applied drought or heat (Pradhan et al. 2012; Perdomo et al. 2015; Tricker et al. 2018). This is attributed to the negative interactions between heat and drought, with the effects becoming additive when combined (Suzuki et al. 2014). Additionally, a meta-analysis using >120 published cases studying crop responses to combined heat and drought revealed that it caused on average twice the decrease in yield (relative to control) compared with exposure to heat stress alone (Cohen et al. 2021). In apparent contrast, in our study, HT had larger impact than D on plant rosette growth and leaf development (Fig. 1, B–D; Supplementary Figs. S5 and S6). This is likely because in our experiments HT was applied from germination onwards, well before D was applied.

Submergence substantially hampered plant growth but did not cause plant death (e.g. Figs. 1 and 5). This is likely because our flooding treatment occurred under the regular day/night regime, which differs from studies where lethality was noted using submergence in darkness (Vashisht et al. 2011; Van Veen et al. 2013). Plants were able to steadily recover if optimal irrigation was applied following de-submergence (Fig. 1, Supplementary Figs. S8 to S10). However, when experiencing drought following de-submergence (PSD), leaf lengthening, rosette area increase, and dry mass accumulation were significantly impacted (Fig. 1, B–D; Supplementary Figs. S8 to S10). This is in line with Luo et al. (2009) who highlighted the essential role of photosynthetic acclimation during submergence recovery (Luo et al. 2009). Interestingly, phenotypic differences between PS and sequential PSD were greater than those between C and D after 10 d (Supplementary Fig. S10). This implies that PS exerted a negative synergic effect on D in regulating plant growth and development in the PSD treatment.

Although both HT and PS interact with D and consequently elicit distinct effects on plant phenotypes under the combined/sequential stress conditions, HT and PS seemingly affect physiological responsiveness to combined HTD and sequential PSD to different degrees. For example, when combined with HT, drought-promoted leaf temperature increase occurred substantially earlier than under individual drought conditions (Supplementary Fig. S7, B and C). In contrast, a submergence pretreatment (S) did not accelerate drought-induced leaf temperature elevation (Supplementary Fig. S11, B and C). Furthermore, stomatal conductance decreased 5 d after combined HTD (Fig. 1E), but not during 5-d PSD (Fig. 1E; Supplementary Fig. S12B). A likely explanation is that stomata are highly susceptible to high temperature, resulting in a pronounced stomatal response when combined with drought (Xu et al. 2023).

Drought can have a major impact on the transcriptome (Bouzid et al. 2019; Chen et al. 2021). However, while our plants clearly experienced drought (e.g. Fig. 1, B–D; Supplementary Figs. S1 and S2), D did not elicit significant transcriptomic changes (3 DEGs, Fig. 2, A and D) nor evoked strong effects on phenotypic traits shortly after stress initiation (Fig. 1, B–D; Supplementary Figs. S3 to S12). This is consistent with a study by Prasch and Sonnewald (2013) in which only 41 DEGs were identified after 5-d progressive drought treatment. The relatively mild drought intensity likely explains the limited number of DEGs (Clauw et al. 2015). In addition, the young leaves that were sampled may be less responsive to drought than for instance roots tissues, as plants might evoke protective mechanisms to prioritize stress protection of younger leaves and meristematic tissues (Verelst et al. 2013; Rankenberg et al. 2024, 2021).

In line with observed unique responses at the phenotypic level, combinatorial stresses imposed distinct and more profound effects on the transcriptome compared with single-stress application (Fig. 2; Supplementary Fig. S14). For both stress combinations, we observed a considerable number of unique multistress-specific DEGs. Notably, the majority of sequential PSD-triggered DEGs were not affected by PS nor D (Fig. 2, D and E). This difference in transcriptome reconfiguration is consistent with the concept that prior exposure to one stress can have consequences for the response to the second stress (Rejeb et al. 2014). We thus propose that changes in gene expression triggered by combined or sequential stresses should be interpreted as a different reprogramming of the transcriptome as compared to the individual stresses, rather than as a simple tweaking of the single-stress responses, although some overlap in DEGs is noted.

Our RNA-seq dataset permitted the identification of genes putatively contributing to morpho-physiological responses to combinatorial and/or individual stresses. To identify these candidate genes, we focused on upregulated TFs and regulatory hubs identified from constructed GRNs (Fig. 2, G and H; Supplementary Fig. S19 and Table S5). Thirty-nine candidate genes identified in this way were experimentally validated to probe their functions in combinatorial stress acclimation. The aim was to unveil genetic effects based on testing a broad range of candidate factors, rather than focusing on deciphering the precise molecular mechanisms underlying selected genes. This approach admittedly neglects downregulated genes, which might also play important roles in mediating responses to combined and/or sequential stresses and are worthwhile to test in future experiments. Also, since the mild HT (at 27 °C), S (in the light), and PS treatments did not cause plant mortality, the survival-related measurements were only conducted on plants exposed to HTD or PSD.

We identified *ELF6* as a negative regulator of phenotypic acclimation to HTD, while *ATL80* is a positive regulator for sequential PSD (Figs. 3, B and D, 4, and 5; Supplementary Figs. S20 and S22). *ELF6* and its homolog *RELATIVE OF EARLY FLOWERING 6* (*REF6*) that lacks a predicted coiled-coil region compared with *ELF6* (Yu et al. 2008), function as histone H3 at Lys27 trimethylation (H3K27me3) demethylases in Arabidopsis (Lu et al. 2011; Crevillén et al. 2014; He et al. 2022). H3K27me3 is crucial for the regulation of plant thermomorphogenesis and heat stress memory (Casal and Balasubramanian 2019; Yamaguchi et al. 2021; Perrella et al. 2022). Therefore, the involvement of *ELF6* in regulating plant growth and development under combined HTD may connect to H3K27me3-mediated high-temperature responses. ATL80 is a plasma membrane-localized RING E3 ubiquitin ligase that negatively regulates cold stress response and phosphate mobilization (Suh and Kim 2015). ATL80 was recently found to potentially target components within the retrograde signaling pathways for degradation (Méndez-Gómez et al. 2024). Additionally, we identified regulators such as *ZFP4* and *GUN4*, as factors in controlling plant wilting under combined HTD or sequential PSD. *ZFP4* and *GUN4* appear to participate in rosette expansion and/or dry weight accumulation under individually applied stresses such as D, HT, and PS, but not combinatorial stresses (Figs. 3, B and D, 4, and 5; Supplementary Figs. S20 and S22). Further investigations are needed to decipher their precise roles in controlling (combinatorial) stress acclimation.

## Conclusions

From the observed diversity in affected phenotypic traits across the tested mutants, treatments, and timepoints (Figs. 3 to 6, Supplementary Figs. S20 to S25), we conclude that different aspects of acclimation strategies of plants to deal with combined HTD or sequential PSD are coordinated by a multitude of genetic factors. These factors together determine certain phenotypic outcomes depending on developmental (st)age and relative stress levels. For example, in response to HTD, TFs such as TLP18.3, BTL13, TCP22, and HB2 are likely involved in orchestrating leaf elongation, possibly as negative regulators, as their corresponding mutant lines displayed induced relative leaf lengthening (Fig. 6, A and C). Upon sequential PSD treatment, HB1 and CDF3 seemingly inhibit the lengthening of petiole and blade (Fig. 6, B and D).

Altogether, our work contributes to a better understanding of plant response to sublethal combinatorial stresses. Generated insights can contribute to the establishment of mechanistic models for predicting trait responsiveness under the given stress condition. In addition, it should be investigated how sublethal combinatorial stresses affect productivity in agronomical crops and whether the multitude of identified multistress regulators also contribute to resilience of commercial crops. Such investigations are critical considering the increased co-occurrence of these stresses in the context of climate change. This work has therefore the potential to contribute to breeding and/or engineering of field crops that maintain optimal yields in future climate conditions.

## Materials and methods

### Plant materials, growth conditions, and stress treatments

*Arabidopsis thaliana* Col-0 seeds were sown on Primasta potting soil (Asten, The Netherlands) and stratified 4 d in darkness. Plants were grown in MD1400 (Snijders, The Netherlands) climate chambers under 8-h photoperiod, 21 °C or 27 °C (day and night), 130 to 150 *μ*mol m^−2^ s^−1^ PAR with a fluorescence tube or LED lightening, and 70% RH. Plants that reached the 2 true-leaf stage were transplanted to Jiffy 7c coconut pellet substrate (Jiffy Products International BV, Zwijndrecht, The Netherlands) that were presoaked in water and 50 mL Hoagland solution (Millenaar et al. 2005) (saturated weight = 250 ± 20 g), contained in 9 × 9 cm pots for stability. Additional Hoagland solution was supplied 2, 5, and 7 d after transplantation. Every second day, water was added and pots were randomized to account for position effects.

Plants containing 10 true leaves (LS10) were subjected to sublethal stress conditions 2 h after the photoperiod started or remained in control conditions (C/HT). Progressive drought (HTD/D) consisted of withholding watering. Drought severity, expressed as relative %SWC, refers to the ratio between the measured pellet weight and weight at full field capacity (Supplementary Fig. S1, A and B). Plants subjected to submergence (PS and PSD) were pregrown and treated at 21 °C. One day before treatment initiation, plastic tubs (54 × 27 × 37 cm) were disinfected with a chlorine tablet (Diversey Inc., Racine, USA) and thereafter rinsed. The tubs were filled with deionized water and allowed to equilibrate to the climate room's temperature. Plants were completely submerged (S) for 5 d while receiving the regular day/night treatment. Thereafter, the pellets containing the plant were covered by absorbent papers to remove excess water until the weight was comparable to nonsubmerged control (C) and were then either subjected to the regular regime of irrigation (PS), or to progressive drought conditions by withholding watering (sequential PSD).

### Morphological analyses

To assess leaf angles, the 2 most hyponastic intermediate-aged leaves were imaged after placement perpendicular to a camera. Plant shoots were thereafter detached, flattened using transparent paper, and imaged from the top. ImageJ software (National Institutes of Health, USA) was used to determine leaf traits. For hyponasty, leaf angle relative to the horizontal was measured by taking the average angle of the captured opposing leaves.

To quantify dry weight, plants were dried in an oven set at 80 °C and weighed on an analytic balance (Sartorius BP221S, Göttingen, Germany). To quantify leaf initiation (rate), the number of all visible leaves (excluding cotyledons) was scored daily. Primary root length was determined using a ruler, after the pellet substrate was rinsed from the roots with tap water.

### Physiological trait analyses

Chlorophyll content was determined using a CCM-300 Chlorophyll Content Meter (Opti-Sciences lnc., Hudson, USA). After calibration, the detector was placed at 5 mm distance from the leaf and chlorophyll content was calculated based on the fluorescence intensity. For destructive biochemical detection, chlorophyll was extracted from detached leaves using 96% DMSO. Absorption was determined at 664, 647, and 750 nm wavelengths using a spectrophotometer plate reader (Synergy HT Multi-Detection Microplate Reader; BioTek Instruments Inc., USA). Dry weight of the remaining plant material was used to calculate relative chlorophyll a, b, and total chlorophyll content.

Relative water content was calculated as (fresh weight − dry weight)/(turgor weight − dry weight)) × 100%. To this aim, fresh weight of excised rosettes was determined using an analytic balance (Sartorius BP221S, Göttingen, Germany). The rosettes were then immediately allowed to saturate with water in petri dishes (24 h in darkness). Excess water was removed and turgor weight of plants was measured. Plants were then dried at 80 °C, and dry weight was recorded.

Stomatal conductance was measured of the abaxial leaf side using an SC-1 Leaf Porometer (Decagon Devices, Inc., Pullman, USA) equilibrated to ambient temperature.

Leaf surface temperatures were recorded using a FLIR A655sc LWIR thermal imaging camera (Teledyne FLIR, USA) with a 13.1 mm FoV 45° × 33.7° hawkeye IR lens. Thermal images were captured every 15 min using FLIR ResearchIR Max 4 software (Teledyne FLIR, USA) from the start of the stress treatments till wilting occurred. Temperatures of 6 leaves per plant were measured at ZT = 0, 6, 12, 18 h, and every 4th image, equaling every 24 h, the regions of interest were adjusted to correct for leaf growth and movement.

To determine MDA content, detached rosettes were ground in ethanol after recording fresh weight and cold-centrifuged. Part of the supernatant was mixed with an equal volume of TCA/thiobarbituric acid (TBA) solution (0.65% (w/v) TBA in 20% (w/v)TCA). The mixture was shaking-incubated at 95 °C for 30 min and cooled in an ice bath. After cold centrifugation, absorbance was measured using a spectrophotometer plate reader (Synergy HT Multi-Detection Microplate Reader; BioTek Instruments Inc., USA) at 532 and 600 nm. MDA concentration was calculated and normalized by fresh weight: MDA (nmol/mL) = ((A532 − A600)/155,000) × 1,000,000.

For quantification of ion leakage, rosettes were detached, placed in a tube containing deionized water, and shaken by rolling (1 h at room temperature [RT]). Initial conductivity was measured using a precalibrated EC-33 conductivity meter (HORIBA, Japan). The samples were then incubated at 95 °C for 30 min and cooled down to RT, after which final conductivity was measured. Ion leakage was calculated as: ion leakage = (initial conductivity/final conductivity) × 100%.

### Transcriptomics sample preparation and analysis

Two of the 7th to 10th leaves, counted from the earliest emerged true leaves at LS10 from 5 to 6 plants, were pooled and flash-frozen in liquid nitrogen and kept at −80 °C. Samples were ground using a cryogenic grinding mill (Retsch, Haan, Germany), and plant total RNA was extracted using the RNeasy kit (Qiagen, Germany) following the manufacturers protocol. In total, 58 samples were collected consisting of 3 to 7 independent biological replicates (leaf pools) across all harvesting timepoints each.

RNA quality control, library construction, and sequencing were performed by Macrogen (Amsterdam, The Netherlands). RNA integrity and purity were determined using an Agilent Technologies 2100 Bioanalyzer (Agilent Technologies, Palo Alto, USA), and only samples with Integrity Number of 7 or above were used for library construction by the TruSeq stranded mRNA protocol (Illumina, USA). Libraries were sequenced using an Illumina Novaseq6000 sequencer (Illumina, USA) with 150 bp pair-end reading. Generated datasets were deposited at the NCBI Gene Expression Omnibus (project ID: PRJNA863409).

Data processing (trimming, read filtering, and mapping) and differential gene expression analysis were conducted as previously described (Verhoeven et al. 2023). Two samples with a total mapped Arabidopsis read count below 7 million due to *ArLV1* infection were removed. For the determination of *ArLV1* effects on plant transcriptome, control (C) samples at timepoint 5 d were selected, as 3 of these samples contained relatively few reads mapping to *ArLV1* (0.01% to 9.56%) and 4 samples contained high amounts of *ArLV1* (78.94% to 90.08%). Only for this analysis, the complete dataset including the 2 low-quality samples was used.

Statistical analysis was performed in R using the log_2_-normalized TPM values in linear models ran for 5 and 10 d timepoints separately, with different stress treatments as variables compared with the control treatment at the same timepoint. Obtained significances were corrected using a Benjamini–Hochberg adjustment (provided by the *prcomp* function). Expressed genes were visualized by ggplot2 (Wickham 2009). In the selection of DEGs, we did not take fold change (FC) into account, given the mild effects of applied stresses on the transcriptome.

*k-means* clustering was used to arrange the significantly regulated genes in clusters with similar expression patterns. The optimal number of clusters was determined visually by plotting the within-cluster sums of squares and the average silhouette with the fviz_nbclust function from the factoextra package in R (Lê et al. 2008). Groups with a high variation in expression between the stress treatments were chosen for additional enrichment analysis.

For the GO enrichment analysis, Metascape (http://Metascape.org/) (Zhou et al. 2019) was used with settings of a *P* value cutoff = 0.01, a minimal number of overlapping genes = 3, and a minimal enrichment value = 1.5.

The locus IDs of Arabidopsis were inputted into Arabidopsis TF database (https://agris-knowledgebase.org/AtTFDB/), and TFs were automatically identified by annotating the candidates to TFs from 50 different TF families. For the construction of GRNs, candidate gene IDs were first imported into TF2Network (Kulkarni et al. 2018) to search for promoter binding sites and thereafter putative upstream TFs were identified. These TFs were then analyzed for co-expression and protein–DNA interactions with the predicted targets and the networks thereafter generated using Cytoscape (Shannon et al. 2003).

### Confirmation of T-DNA insertion lines

gDNA was extracted from leaves using the Direct PCR-Phire and Phusion kit (Thermo Scientific, lnc, USA) following the manufacturers protocol. Presence of T-DNA insertions and homozygosity were determined by PCR (primers see: Supplementary Tables S6 and S7). We were unable to obtain homozygous lines for 12 genes, mainly because of issues with seed germination of obtained seed stocks.

### Statistical analysis

If not explicitly mentioned otherwise, figures were generated using GraphPad Prism 9 (GraphPad Software, La Jolla, USA) or Biorender.com. Statistical analyses were performed using GraphPad Prism 9 or R software. Significance was calculated using an alpha of 0.05.

### Accession numbers

Generated sequence datasets from this article were deposited at the NCBI Gene Expression Omnibus (Project ID: PRJNA863409). Accession numbers (Arabidopsis AGI codes) of differentially expressed TF's and mutants used in this study are indicated in Supplementary Tables S3 to S7.

## Supplementary Material

kiae581_Supplementary_Data

## Data Availability

The transcriptomics data underlying this article are available at the NCBI Gene Expression Omnibus (Project ID: PRJNA863409). Other data is available via the online supplementary materials or will be shared on reasonable request to the corresponding authors.

## Supplementary data

The following materials are available in the online version of this article.

**Supplementary Figure S1.** Progressive decline in %SWC during combined and sequential stresses, single stresses, and control conditions.

**Supplementary Figure S2.** Effect of combined and sequential stresses on leaf initiation and wilting.

**Supplementary Figure S3.** Effect of combined and sequential stresses on chlorophyll content, obtained with a CCM-300 chlorophyll content meter.

**Supplementary Figure S4.** Chlorophyll content of young leaves in control and drought conditions, obtained by a destructive biochemical assay and using a CM-300 chlorophyll meter.

**Supplementary Figure S5.** Effect of combined high temperature and drought and the corresponding single stresses on morphological traits.

**Supplementary Figure S6.** Dynamics in plant petiole and blade length in response to combined high temperature and drought and the corresponding single stresses and controls.

**Supplementary Figure S7.** Effect of combined high temperature and drought and the corresponding single stresses on physiological traits.

**Supplementary Figure S8.** Effect of sequential submergence and drought and the corresponding single stresses on various morphological traits.

**Supplementary Figure S9.** Dynamics in plant petiole and blade length during postsubmergence combined with drought and the relevant single stresses and controls.

**Supplementary Figure S10.** Phenotypic traits as an effect of drought in presence or absence of prior submergence.

**Supplementary Figure S11.** Effect of submergence followed by recovery under drought or well-watered conditions and corresponding single stresses on physiological traits.

**Supplementary Figure S12.** Physiological traits as effect of drought in presence or absent of prior submergence.

**Supplementary Figure S13.** Sequence coverage of RNA-seq dataset and transcriptomic effects of *ArLV1*.

**Supplementary Figure S14.** PCA of transcriptomes of young leaves exposed to combined or sequential stresses, single stresses, or control conditions.

**Supplementary Figure S15.** Correlation matrix of samples in the RNA-seq dataset visualizing the distribution of transcripts of all 56 samples that passed the quality control.

**Supplementary Figure S16.** Relative expression values of 49 known core hypoxia-responsive genes.

**Supplementary Figure S17.** Relative expression values of 33 DEGs regulated by both PS and PSD.

**Supplementary Figure S18.** Relative expression values of 8 TFs that were upregulated in both combined (HTD) and sequential (PSD) stresses, compared with controls (C).

**Supplementary Figure S19.** GRNs for DEGs from the upregulated gene clusters in combined high temperature and drought (HTD) and submergence followed by drought (PSD).

**Supplementary Figure S20.** Effect of combined and individual stresses on leaf development traits of selected mutants.

**Supplementary Figure S21.** Effect of combined and individual stresses on chlorophyll content and stomatal conductance of selected mutants and corresponding WT.

**Supplementary Figure S22.** Effect of sequential and individual stresses on leaf development of selected mutants.

**Supplementary Figure S23.** Effect of sequential and individual stresses on chlorophyll content and stomatal conductance of selected mutants and corresponding WT.

**Supplementary Figure S24.** Typical enhanced leaf hyponasty phenotype of *ilr3* compared with Col-0 WT plants during subjection to high-temperature treatments.

**Supplementary Figure S25.** Representative images of Col-0 WT and *hb34* mutant subjected to control and submergence followed by drought (PSD).

**Supplementary Table S1.** GO enrichment analysis of identified DEG K-means clusters of individual and combined stress treatments

**Supplementary Table S2.** GO enrichment analysis of identified DEG K-means clusters of D, PS and PSD treatments

**Supplementary Table S3.** TFs derived from the upregulated DEG clusters under HTD treatment

**Supplementary Table S4.** TFs derived from the upregulated DEG clusters under PSD treatment

**Supplementary Table S5.** Putative multistress acclimation TFs considered for the reverse genetics approach (mutant analysis) under HTD, PSD, or both treatments

**Supplementary Table S6.** T-DNA insertion mutants used for functional validation under HTD treatment and primers used for genotyping

**Supplementary Table S7.** T-DNA insertion mutants used for functional validation under PSD treatment and primers used for genotyping

**Supplementary Table S8.** Relative phenotypic traits values of tested mutants under HTD

**Supplementary Table S9.** Relative phenotypic traits values of tested mutants under PSD

## Dive Curated Terms

The following phenotypic, genotypic, and functional terms are of significance to the work described in this paper:

ZFP4 Gramene: AT1G66140
ZFP4 Araport: AT1G66140

RPL Gramene: AT5G02030
RPL Araport: AT5G02030

CDF3 Gramene: AT3G47500
CDF3 Araport: AT3G47500

RBL Gramene: AT3G55510
RBL Araport: AT3G55510

vip1 Gramene: AT1G43700
vip1 Araport: AT1G43700

at5g12310 Gramene: AT5G12310
at5g12310 Araport: AT5G12310

at3g10760 Gramene: AT3G10760
at3g10760 Araport: AT3G10760

GUN4 Gramene: AT3G59400
GUN4 Araport: AT3G59400

AT5G16990 Gramene: AT5G16990
AT5G16990 Araport: AT5G16990

PFK1 Gramene: AT4G29220
PFK1 Araport: AT4G29220

ELF6 Gramene: At5g04240
ELF6 Araport: At5g04240

ABI5 Gramene: AT2G36270
ABI5 Araport: AT2G36270

NAC CHEBI: CHEBI:7421

h3k27me3 CHEBI: CHEBI:85045
