# Supplementary material for: Deciphering acclimation to sublethal combined and sequential abiotic stresses in Arabidopsis thaliana
Source: Plant Physiol. 2024 Oct 29;200(3):kiae581. doi: 10.1093/plphys/kiae581 (PMC13016629; doi:10.1093/plphys/kiae581)
Supplement: kiae581_Supplementary_Data [file kiae581_supplementary_data.zip › PP2024RA00944R1_Supplemental_Figures.pdf]

Supplementary Data

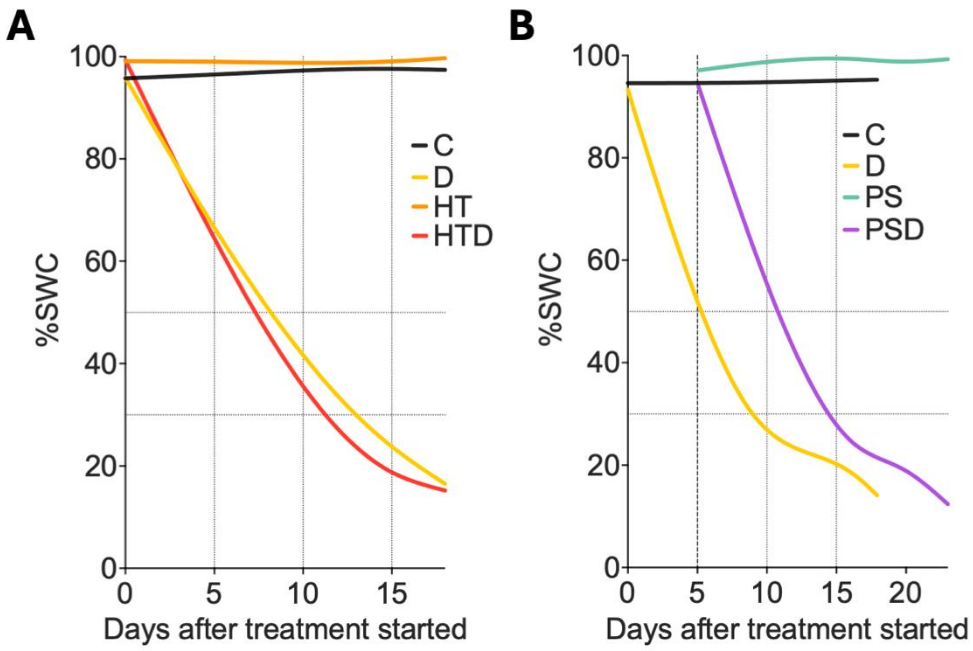

Supplementary Figure S1. Progressive decline in soil water content (%SWC) during combined and sequential stresses, single stresses, and control conditions. Indicated are declines %SWC during combined high temperature and drought **(A)** and post submergence followed by drought **(B)**, and the composite single stresses and control. (A) n = 3-18. (B) n = 8-34. Abbreviations; C: control (black lines), D: drought (yellow), HT: high temperature (orange), HTD: high temperature & drought (red), S/PS: 5-day submergence / (post-)submergence & recovery (blue), PSD: (post-)submergence & drought (purple).

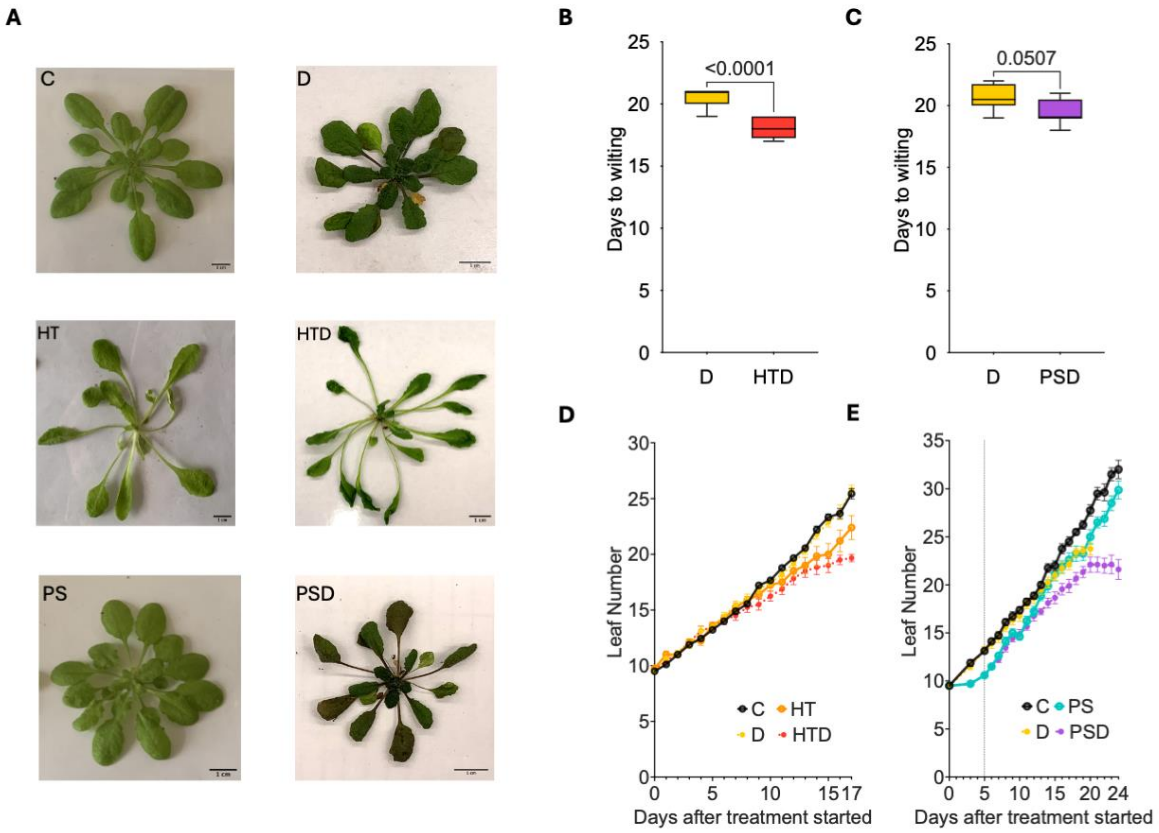

Supplementary Figure S2. Effect of combined and sequential stresses on leaf initiation and wilting. **(A)** Representative images showing Arabidopsis Col-0 rosettes at the wilting stage (right column; non-turgid) after progressive drought and the relative controls (left column; turgid non-wilted plants) at control temperature (21 °C, C and D, upper row), high temperature (27 °C) combined with drought (HT and HTD, middle row) and post submergence followed by drought (PS and PSD, bottom row). **(B, C)** Number of days until wilting occurred in plants subjected to (B) drought at control temperature (21 °C; D) and at high temperature (27 °C; HTD) and (C) drought at control temperature (21°C; D) and post-submergence followed by drought (PSD). Days were counted after water was withdrawn. (B) n = 8-10. (C) n = 8-9. Boxes indicate boundaries of the second and third quartiles (Q) of the data distribution. Black horizontal bars indicate median and whiskers Q1 and Q4 values within 1.5 times the interquartile range. Numbers above the bars indicate p values (unpaired t-test). **(D, E)** Leaf number of plants exposed to combined high temperature and drought (D) (HTD) or post-submergence followed by drought (E) (PSD) and the associated single stresses (HT, HTD, D, PS, PSD) and controls (C). Error bars indicate means ± SEM, (D) n = 6-18. (E) n = 5-33. The dashed vertical line in panel (E) indicates the moment plants were de-submerged. For treatment abbreviations and used colors, see legend of Supplementary Figure S1.

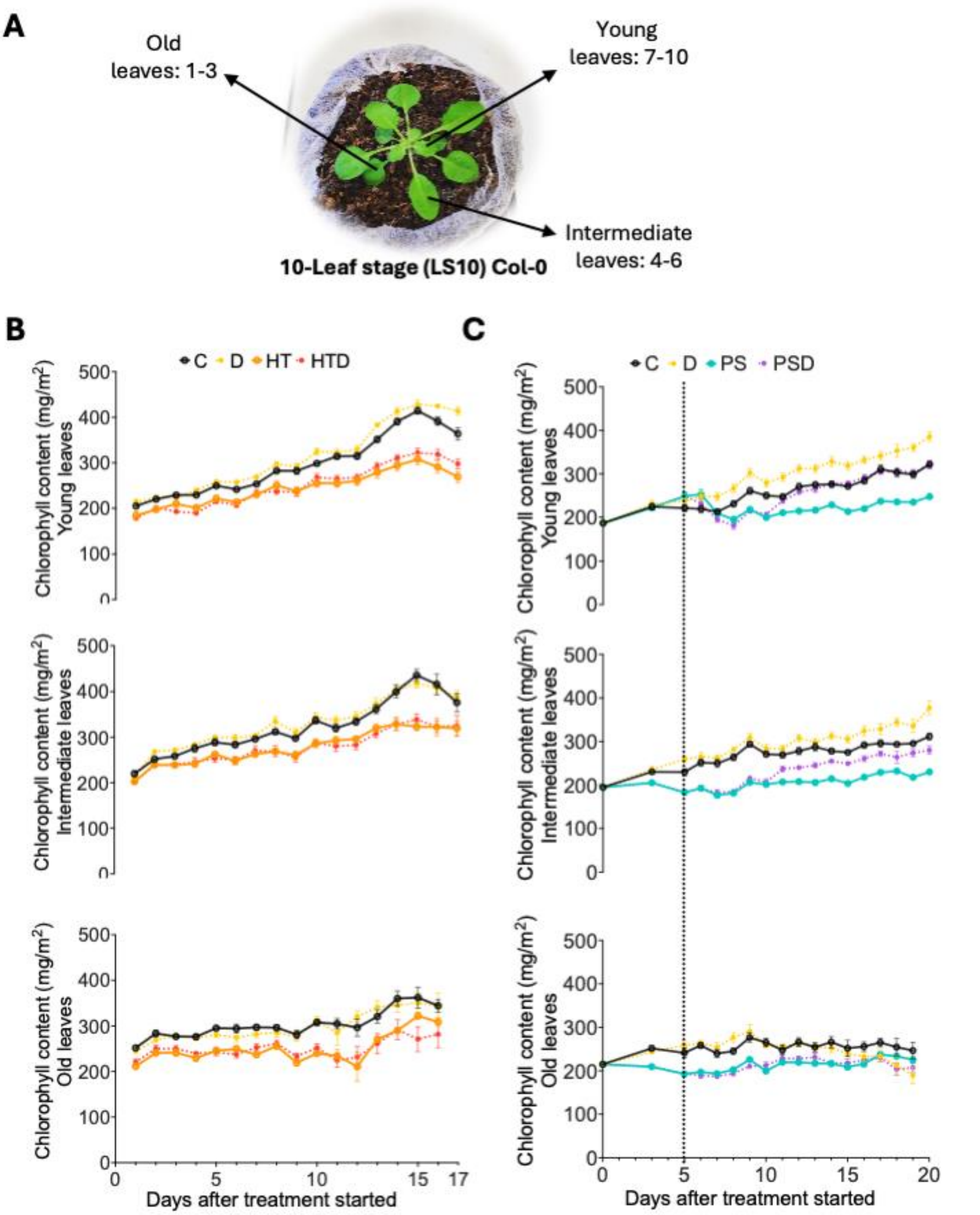

Supplementary Figure S3. Effect of combined and sequential stresses on chlorophyll content, obtained with a CCM-300 chlorophyll content meter. **(A)** Representative image of a 10-leaf stage (LS10) Col-0 plant on Jiffy 7c coconut pellet growth substrate, with young, intermediate, and old leaves indicated. Associated leaf numbers are counted starting from the first true leaves (thus excluding the cotyledons). **(B, C)** Chlorophyll content of young (upper row), intermediate (middle row), and old leaves (lower row) exposed to high temperature & drought (HTD) (B) or post-submergence & drought (PSD) (C), as well as the associated single stresses (HT, PS, D). Error bars indicate means ± SEM, (A) n = 4-9. (B) n = 5-33. The dashed vertical line in panel (C) indicates the moment plants were de-submerged. For treatment abbreviations and used colors, see legend of Supplementary Figure S1.

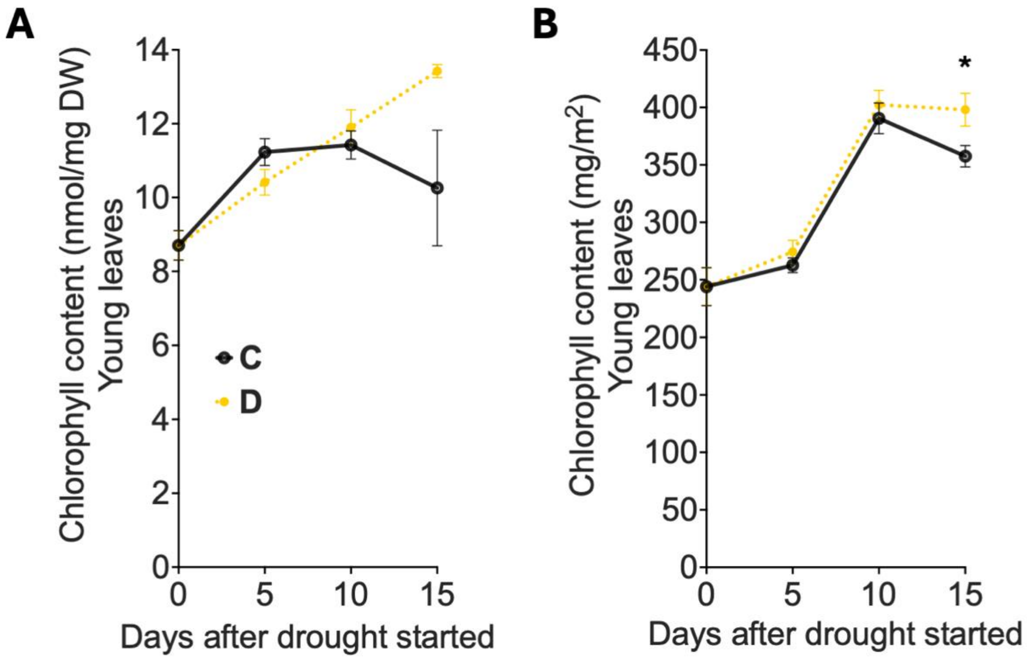

Supplementary Figure S4. Chlorophyll content of young leaves in control and drought conditions, obtained by a destructive biochemical assay and using a CM-300 chlorophyll-meter. Harvests and measurements were conducted at 0, 5, 10, and 15 days after drought (D) started and compared to control (C) conditions. Data indicated are obtained by destructive biochemical assay **(A)** and by using a CM-300 chlorophyll-meter **(B)**. Error bars indicate means ± SEM. n = 4-5. Asterisks represent significant differences between drought and control plants within the same time point ($p < 0.05$, unpaired t-test). For treatment abbreviations and used colors, see legend of Supplementary Figure S1.

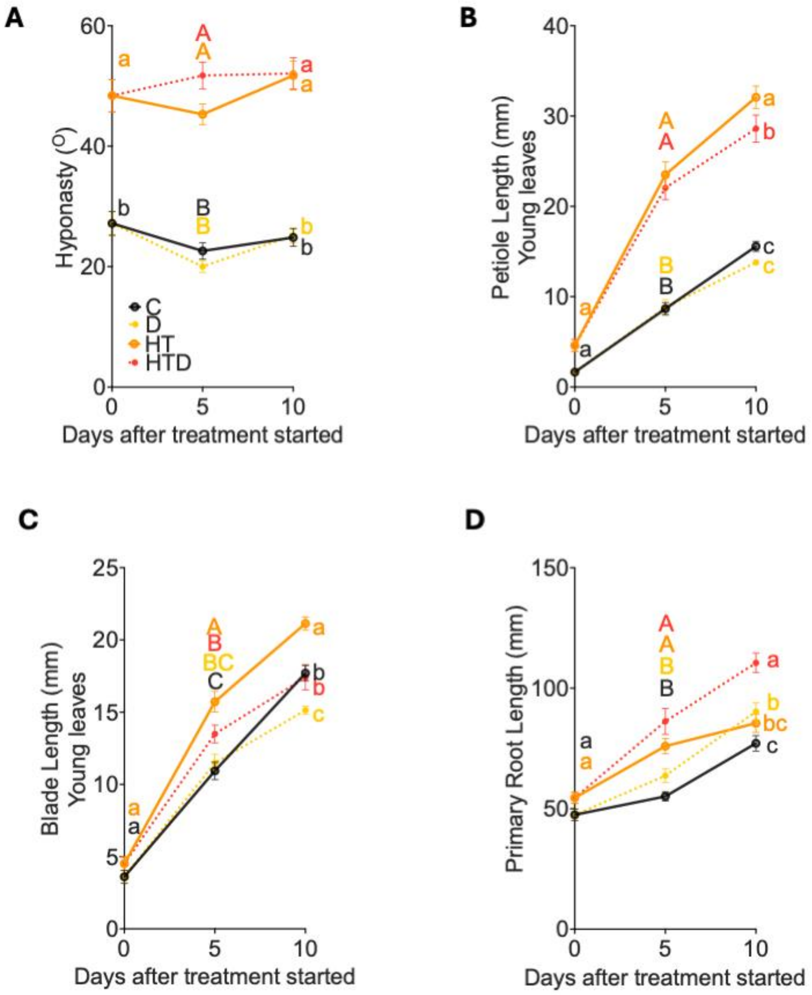

Supplementary Figure S5. Effect of combined high temperature and drought and the corresponding single stresses on morphological traits. **(A)** Average angles of the two most hyponastic leaves of individual plants, relative to the horizontal. n = 14-21. **(B, C)** Average length of petiole (B) and blade (C) of young leaves. n = 15-21. **(D)** Primary root length. n = 14-21. Error bars indicate means ± SEM. Letters denote significant differences between different treatments at the same time points ($p < 0.05$, 2-way ANOVA with Tukey's Post-hoc test). For treatment abbreviations and used colors, see legend of Supplementary Figure S1.

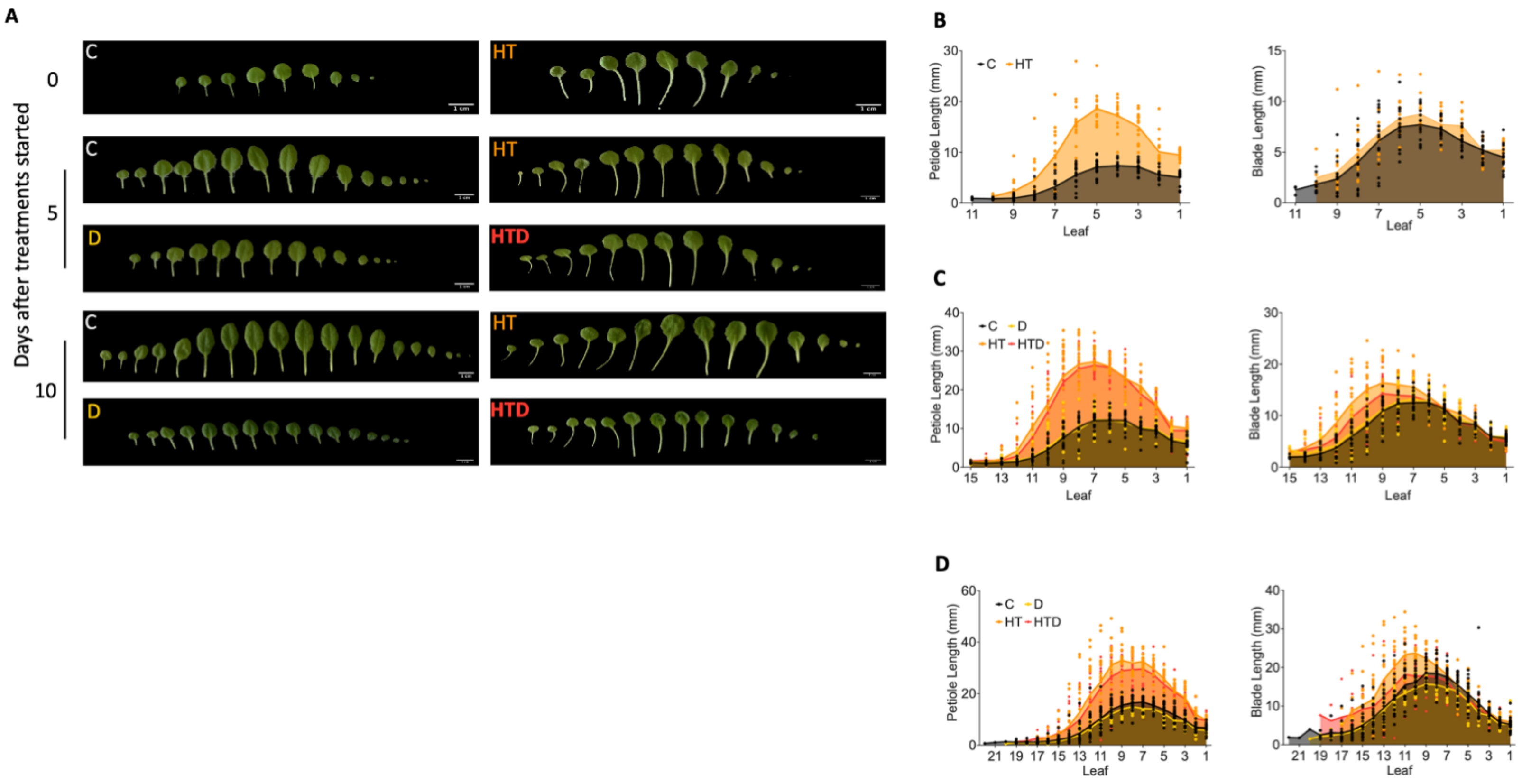

Supplementary Figure S6. Dynamics in plant petiole and blade length in response to combined high temperature and drought and the corresponding single stresses and controls. **(A)** Representative images of dissected leaves (ordered from old to young, with the cotyledons on the far-right side) from plants subjected to single and combined stresses at 0, 5, and 10 days, counted from the start of stress treatments. Images were digitally extracted for comparison. Scale bars indicate 1 cm. **(B, C, D)** Petiole (left) and blade (right) lengths of all leaves from plants subjected to high temperature combined with drought and related single stresses and control at 0 (B), 5 (C) and 10 (D) days after the start of stress treatments. Dots and lines indicate individual plants and averaged data, respectively. n = 15-21. For treatment abbreviations and used colors, see legend of Supplementary Figure S1.

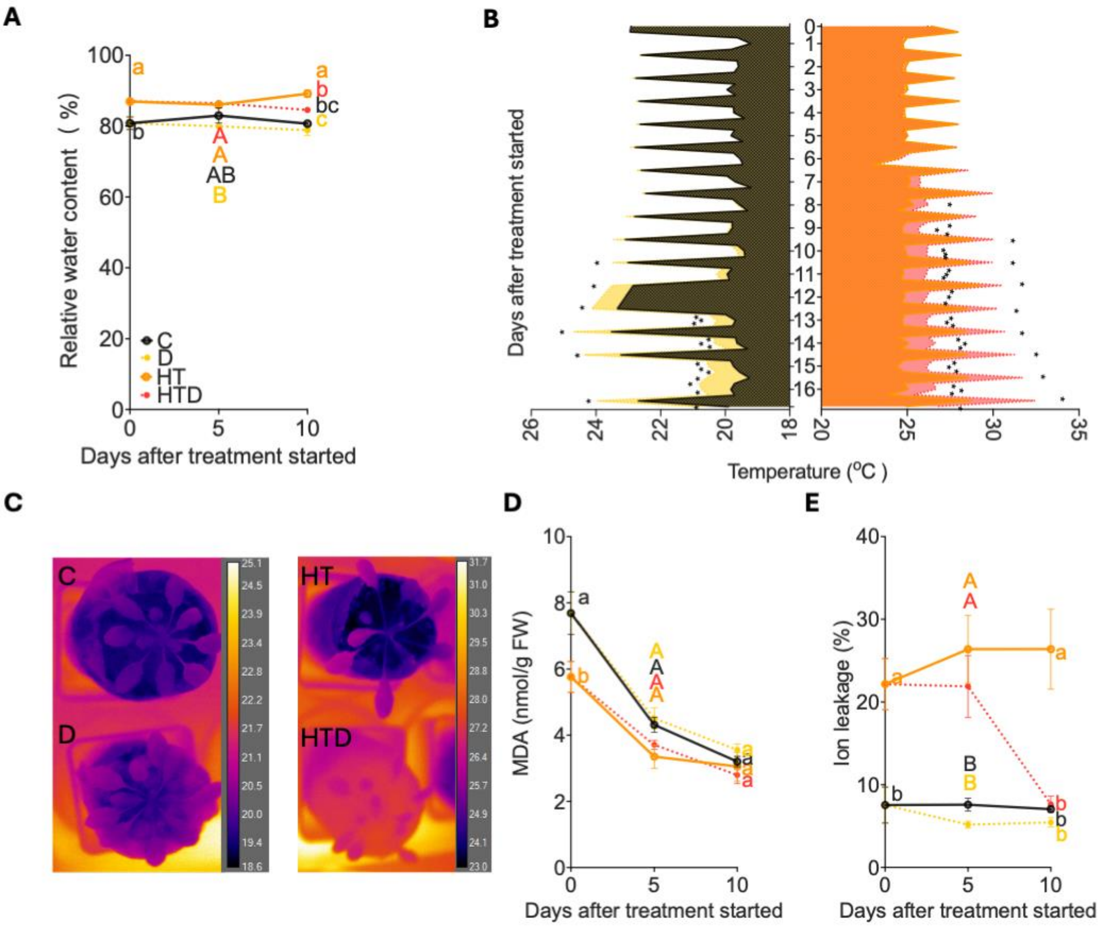

Supplementary Figure S7. Effect of combined high temperature and drought and the corresponding single stresses on physiological traits. **(A)** Rosette water content relative to the maximal water content that the leaves can hold (100%, turgid water - dry weight). n = 8-11. **(B)** Dynamic changes in leaf surface temperature at 21 °C (C and D, left) and 27 °C (HT and HTD, right). Lines represent the average leaf temperature as measured every 6 hours (ZT = 0, 6, 12, and 18 h). n=3. Asterisks represent significant differences between measured leaf temperature between drought (D and HTD) and well-watered plants (C and HT) within the same timepoints (p < 0.05, One-way ANOVA with Tukey's Post-hoc test). Note that temperature fluctuates between the photoperiod (peak) and dark period. Data from three time points (ZT = 18 h on day 11 and ZT = 0 and 6 h on day 12 in 21 °C) were not recorded due to camera failure. **(C)** Representative thermal images of plants at 21 °C (C and D-treated, left) and 27 °C (HT-treated and HTD-treated, right) 10 days after the treatments started. **(D)** Rosette Malondialdehyde (MDA) content. n = 5-10. **(E)** Rosette ion leakage relative to the maximal electrolyte conductivity (100%). n = 6. (A, D, E) Error bars indicate means ± SEM. Letters denote significant differences between treatments within the same time points (p < 0.05, 2-way ANOVA with Tukey's Post-hoc test). For treatment abbreviations and used colors, see legend of Supplementary Figure S1.

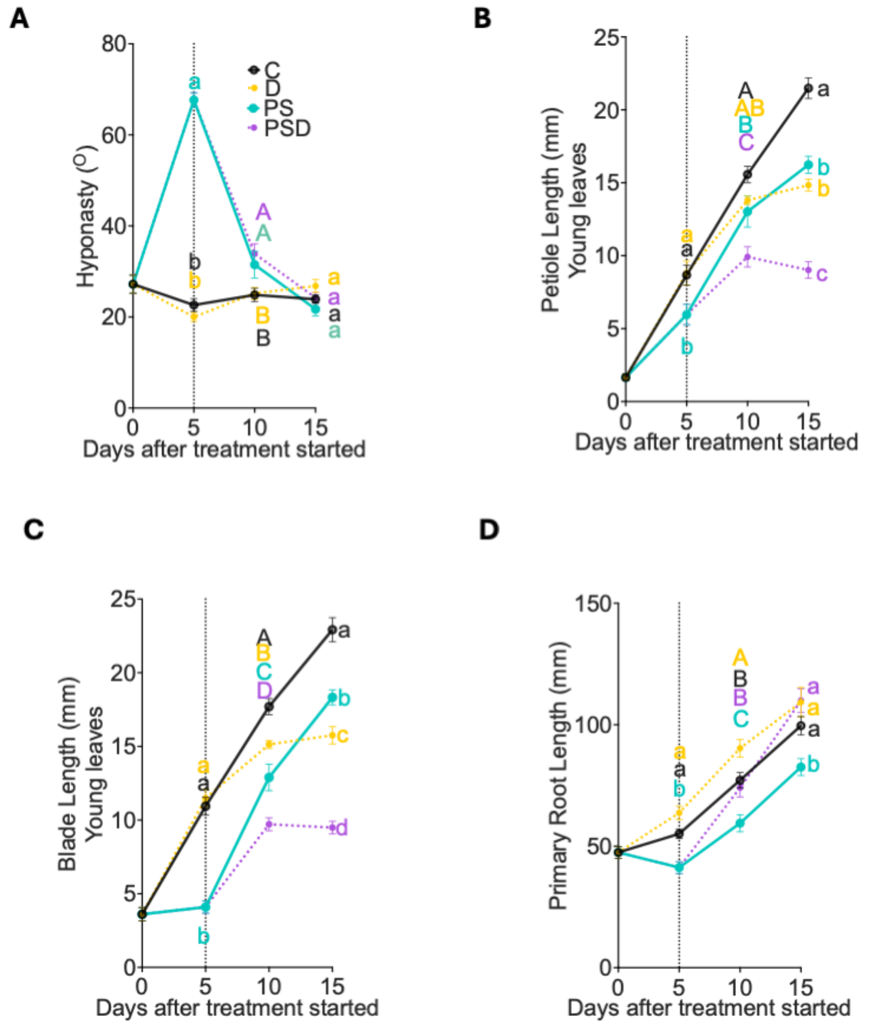

Supplementary Figure S8. Effect of sequential submergence and drought and the corresponding single stresses on various morphological traits. **(A)** Average angles of the two most hyponastic leaves of individual plants, relative to the horizontal. n = 14-21. **(B, C)** Average length of petiole (B) and blade (C) of young leaves. n = 15-21. **(D)** Primary root length. n = 14-21. Error bars indicate means ± SEM. Letters denote significant differences between different treatments at the same time points ($p < 0.05$, 2-way ANOVA with Tukey's Post-hoc test). For treatment abbreviations and used colors, see legend of Supplementary Figure S1.

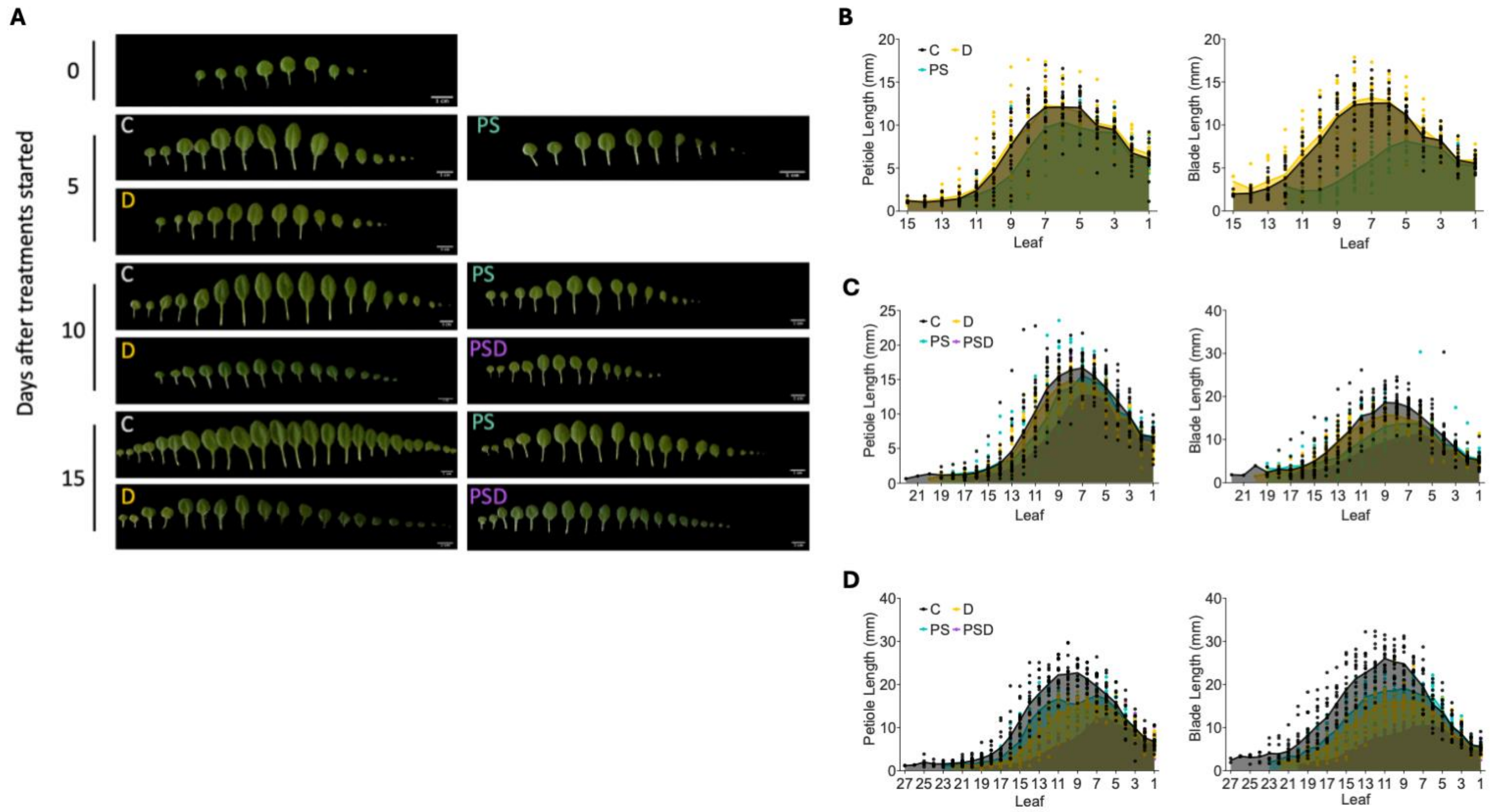

Supplementary Figure S9. Dynamics in plant petiole and blade length during post-submergence combined with drought and the corresponding single stresses and controls. **(A)** Representative images of dissected leaves (ordered from old to young, with the cotyledons on the far-right side) from plants subjected to single and sequential stresses at 0, 5, 10 and 15 (C and D) days, counted from the start of the stress treatments. Images were digitally extracted for comparison. Scale bars indicate 1 cm. (B, C, D) Petiole (left) and blade (right) lengths of all leaves from plants subjected to post-submergence followed by drought and related single stresses and control at 0 (B), 5 (C) and 10 (D) days after the start of de-submergence phase. Dots and lines indicate individual plants and averaged data, respectively. n = 15-21. For treatment abbreviations and used colors, see legend of Supplementary Figure S1.

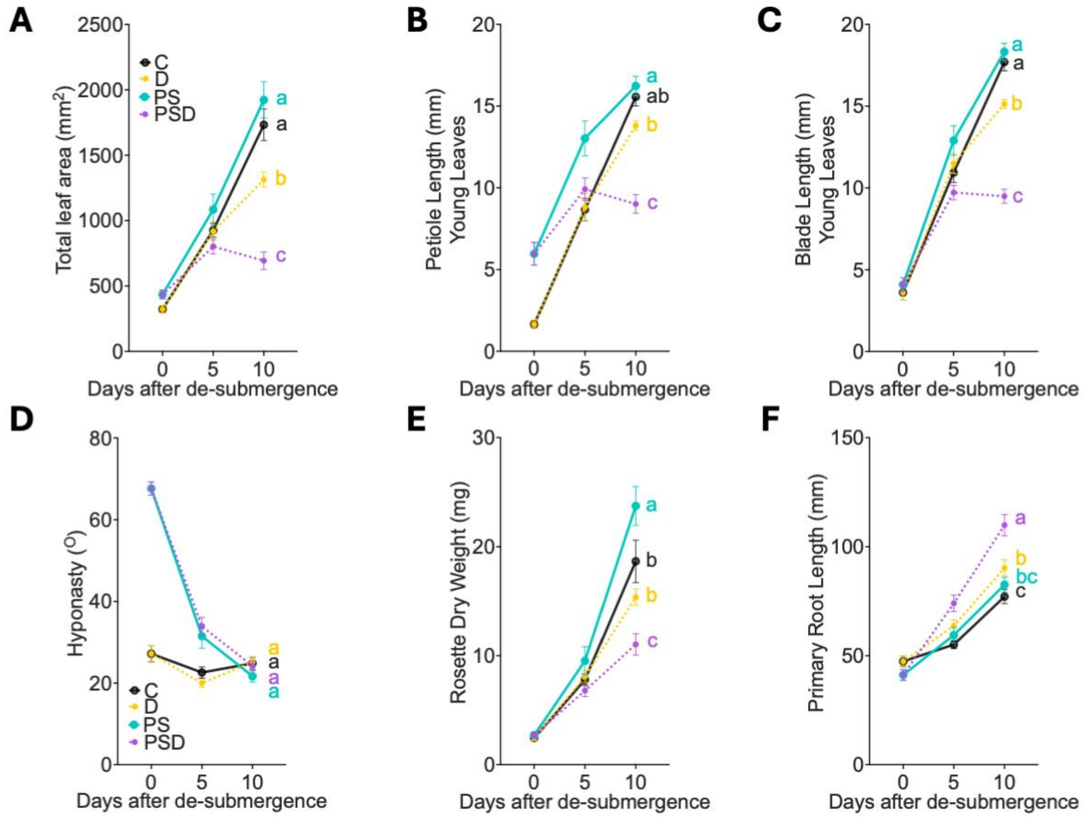

Supplementary Figure S10. Phenotypic traits as an effect of drought in presence or absence of prior submergence. **(A)** Total leaf area of the whole rosette. n = 15-20. **(B, C)** Averaged length of the petiole (B) and blade (C) of young leaves. n = 15-21. **(D)** Averaged angles of the 2 most hyponastic leaves of individual plants, relative to the horizontal. n = 14-21. **(E)** Rosette dry weight. n = 15-18. **(F)** Primary root length n = 14-21. Error bars indicate means ± SEM. Letters denote significant differences between different treatments the stress treatments ($p < 0.05$, 2-way ANOVA with Tukey's Post-hoc test). Data presented here is derived from Figure 1 and Supplementary Figure S8. For treatment abbreviations and used colors, see legend of Supplementary Figure S1.

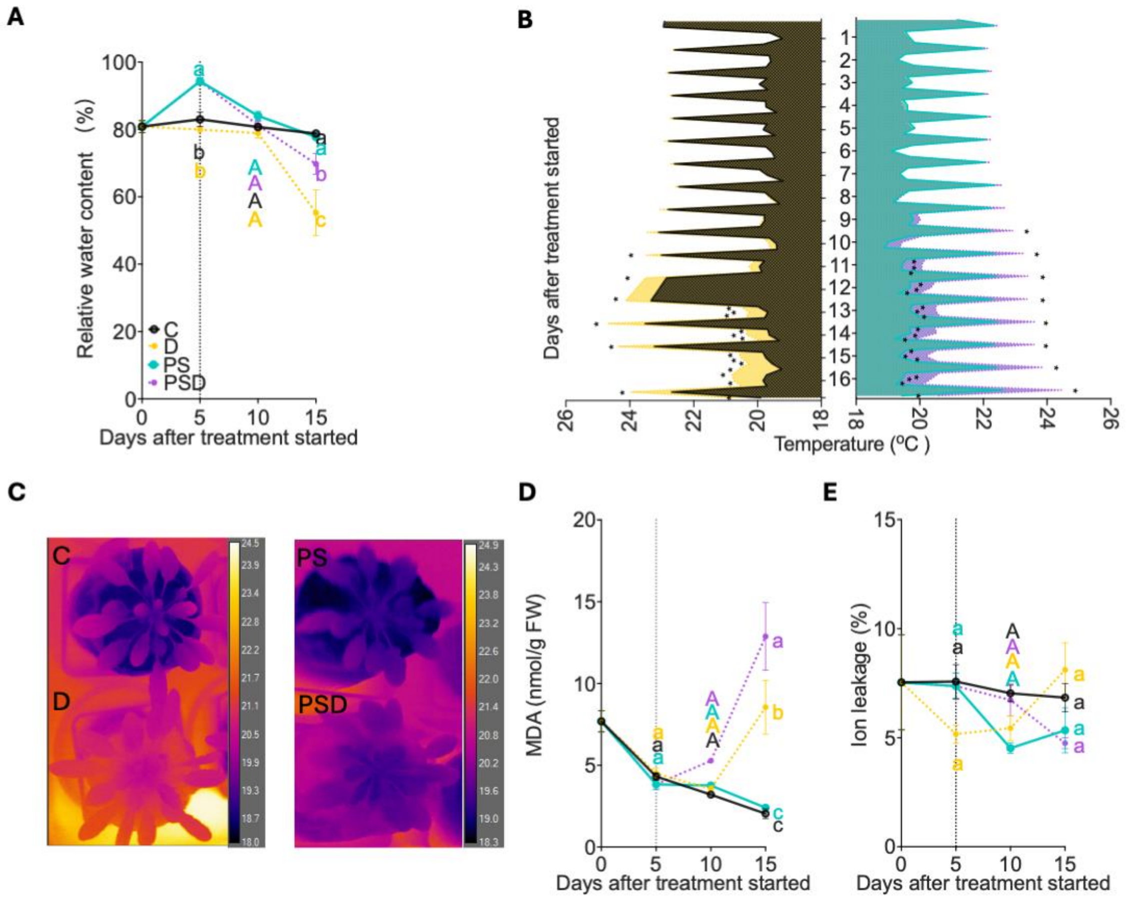

Supplementary Figure S11. Effects of submergence followed by recovery under drought or well-watered conditions and the corresponding single stresses on physiological traits. **(A)** Rosette water content relative to the maximal water content that the leaves can hold (100%, turgid water - dry weight). n = 15-16. **(B)** Dynamic changes in leaf surface temperature of plants with (PS & PSD, right) and without (C & D, left) pre-submergence treatment. Lines represent the average leaf temperature measured every 6 hours (ZT = 0, 6, 12, and 18 h). n = 3. Asterisks represent significant differences between measured leaf temperature within the same timepoints ($p < 0.05$, One-way ANOVA with Tukey's Post-hoc test). Note that temperature fluctuates between the photoperiod (peak) and dark period. Data from three time points (ZT = 18 h on day 11 and ZT = 0 h and 6 on day 12 in 21 °C) were not recorded due to the camera failure. **(C)** Representative thermal images of plants in absence of prior submergence subjected to control and drought treatments for 15 days (C and D, left), or subjection to submergence followed by either well-watered condition or drought for 10 days (PS and PSD, right). **(D)** Rosette malondialdehyde (MDA) content. n = 5-10. **(E)** Rosette ion leakage relative to the maximal electrolyte conductivity (100%). n = 6. (A, D, E) Error bars indicate means ± SEM. Letters denote significant differences between different treatments within the same time points ($p < 0.05$, 2-way ANOVA with Tukey's Post-hoc test). The dashed vertical lines indicate the moment plants were de-submerged. For treatment abbreviations and used colors, see legend of Supplementary Figure S1.

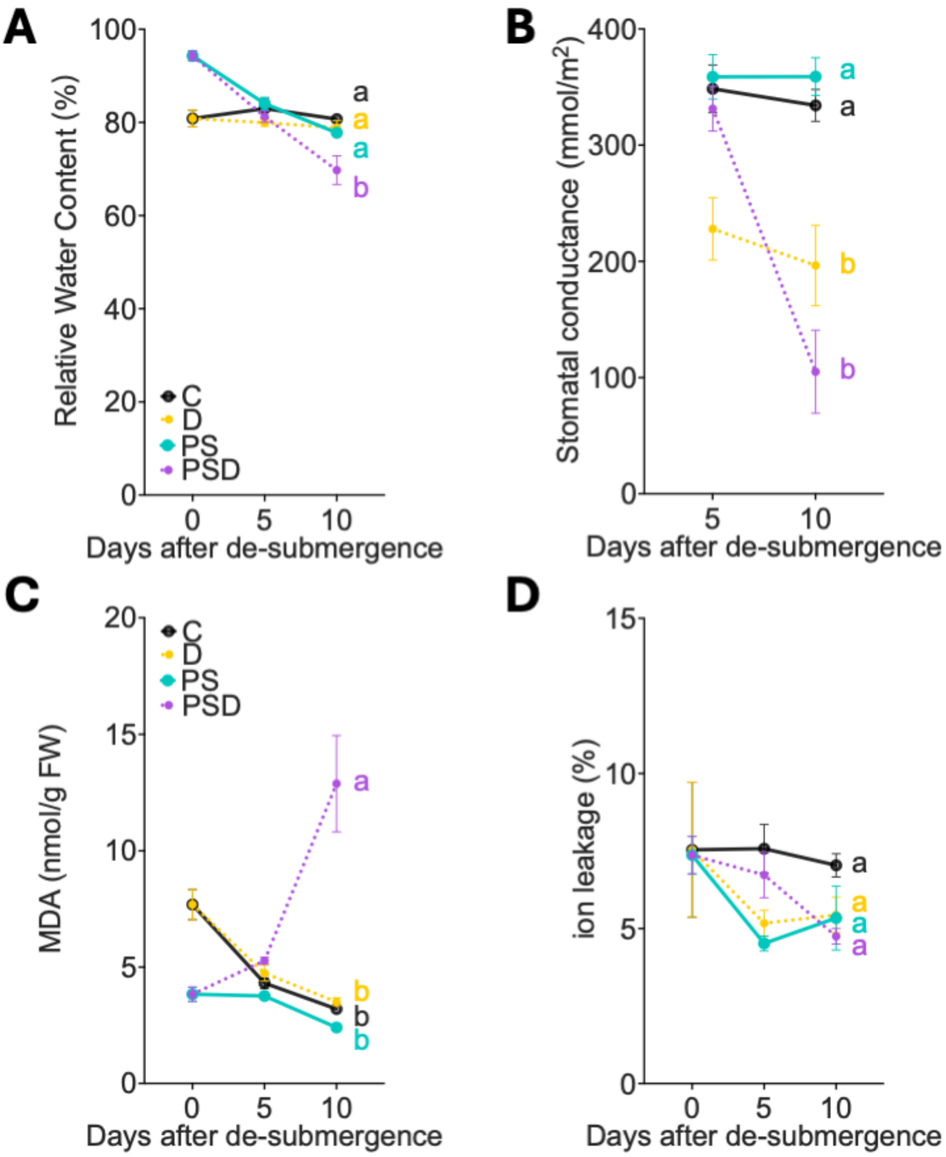

Supplementary Figure S12. Physiological traits as effect of drought in presence or absent of prior submergence. **(A)** Rosette water content relative to the maximal water content that the leaves can hold (100%, turgid water - dry weight). n = 15-16. **(B)** Stomatal conductance of young leaves. n = 5-6. **(C)** Rosette Malondialdehyde (MDA) content. n = 5-10. **(D)** Rosette ion leakage relative to the maximal electrolyte conductivity (100%). n = 6. Error bars indicate means ± SEM. Letters denote significant differences between different treatments at the last timepoint of the stress treatments ($p < 0.05$, 2-way ANOVA with Tukey's Post-hoc test). Data presented here is derived from Figure 1 and Supplementary Figure S11. For treatment abbreviations and used colors, see legend of Supplementary Figure S1.

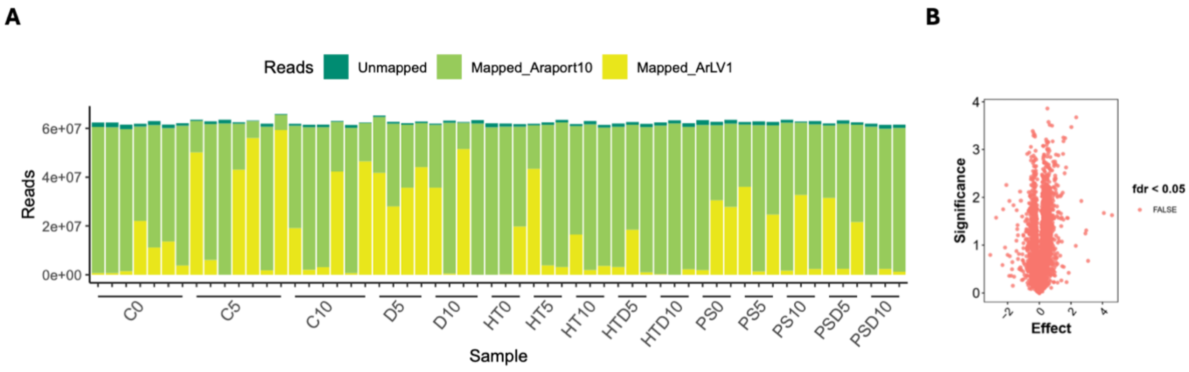

Supplementary Figure S13. Sequence coverage of RNA-seq dataset and transcriptomic effects of *ArLV1*. **(A)** Number of sequenced reads mapping to known Arabidopsis genes part of the transcriptome (Mapped_Araport10, green), RNA1 and RNA2 of the *ArLV1* virus (Mapped_ArLV1, yellow) or neither of the two (Unmapped, dark green). **(B)** Volcano plot indicating transcriptomic differences between samples mapping above and below 50% to the *A. thaliana* transcriptome in Control (C) plants at t = 5 days. Genes with FDR (false detection rate) < 0.05 are considered significant.

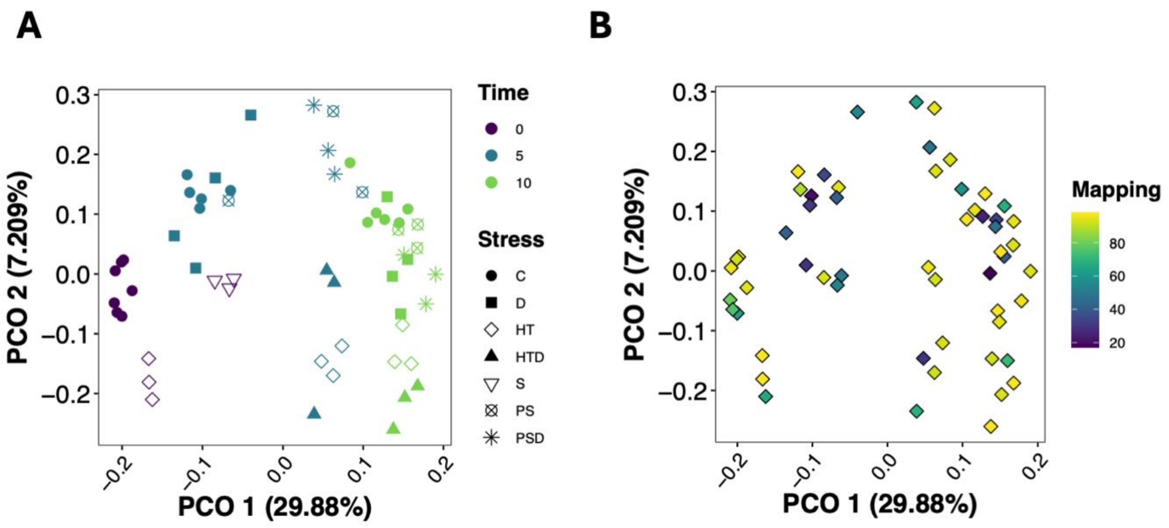

Supplementary Figure S14. PCA of transcriptomes of young leaves exposed to combined or sequential stresses, single stresses, or control conditions. **(A, B)** PCA analysis visualizing distributions of samples categorized by time (0 = purple, 5 days = blue and 10 days = green) and the different stresses, indicated by symbols (A) and the fraction of reads per sample mapping to the Arabidopsis transcriptome (B). Fraction of reads mapping to the Arabidopsis reference transcriptome is presented by a color scale. For treatment abbreviations see legend of Supplementary Figure S1.

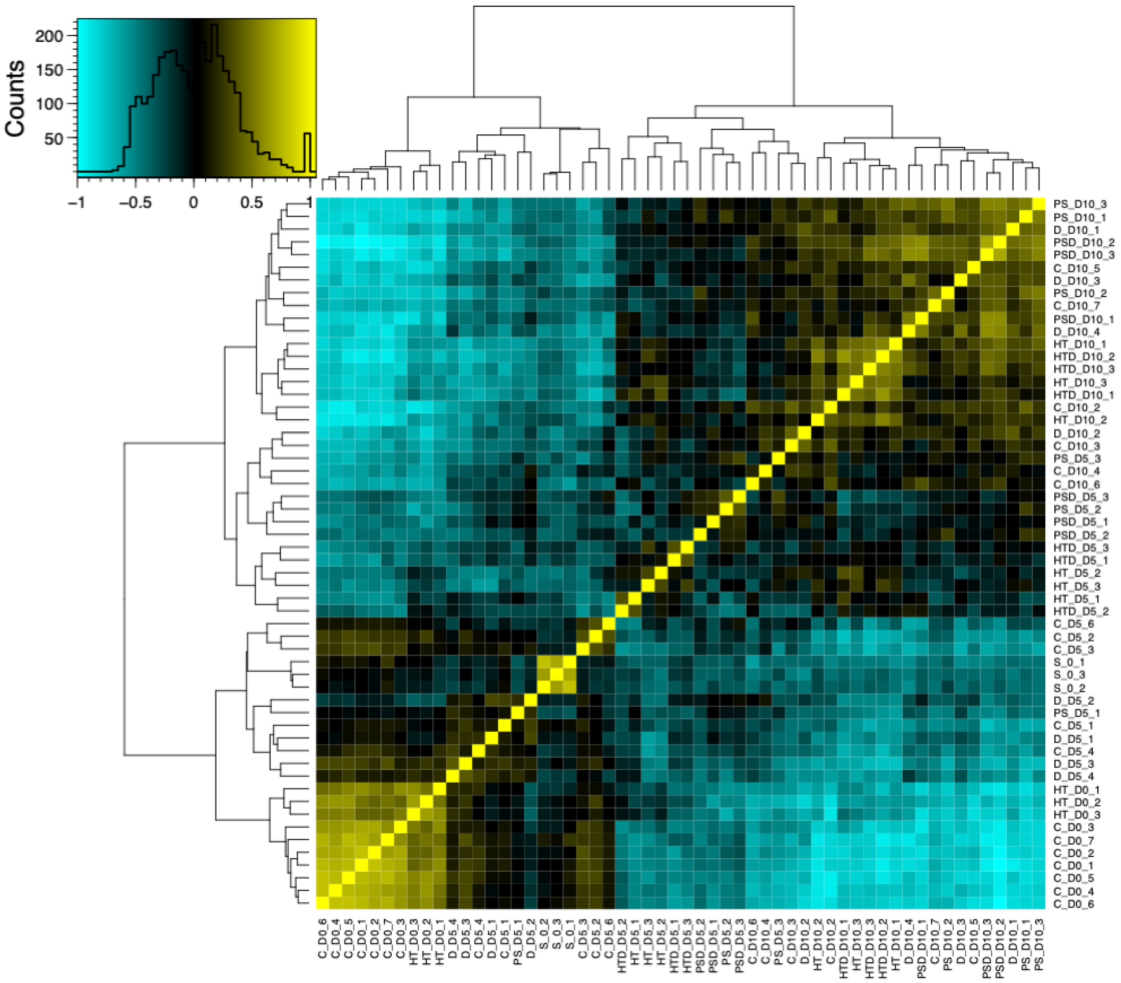

Supplementary Figure S15. Correlation matrix of samples in the RNA-seq dataset visualizing the distribution of transcripts of all 56 samples that passed the quality control. The color scale indicates the strength of the correlation (negative: blue, positive: yellow) and the distribution of the matrix values. The relatedness of individual samples are indicated by the hierarchal clustering trees. Samples are named as; treatment abbreviation_timepoint_biological replicate number. For treatment abbreviations see legend of Supplementary Figure S1.

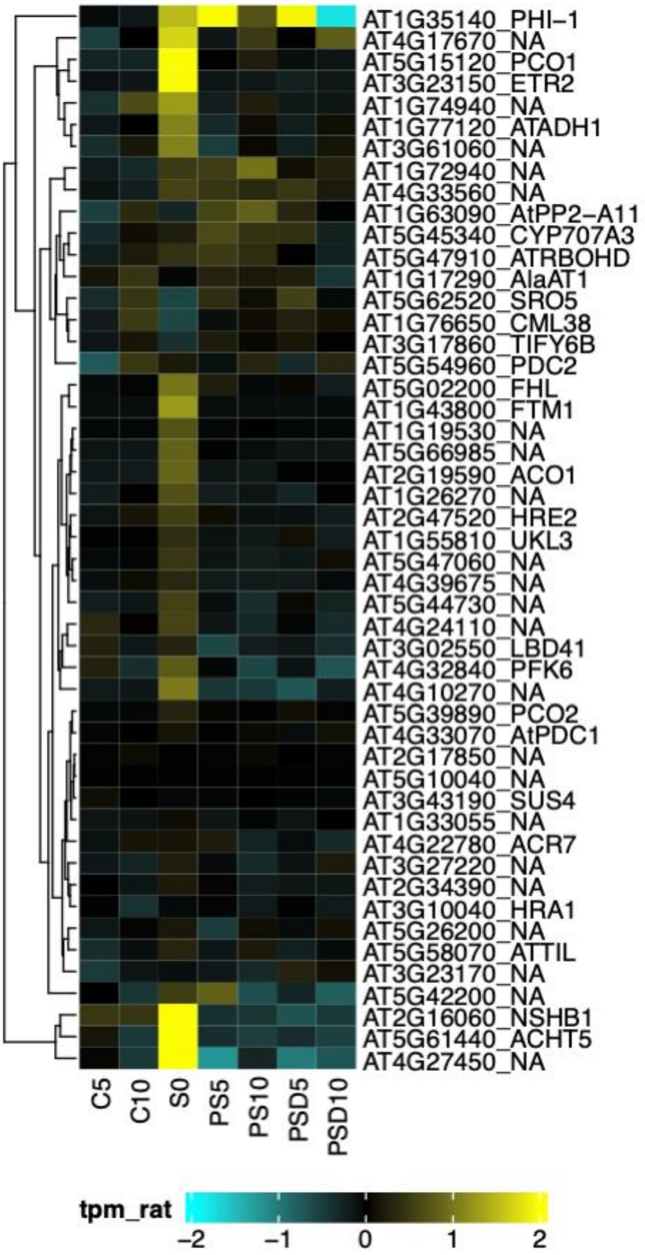

Supplementary Figure S16. Relative expression values of 49 known core-hypoxia responsive genes (Mustroph et al., 2009). Heatmap represents the expression ratio in transcripts per million (TPM) of hypoxia responsive genes in response to submergence (S) followed by drought (PSD) and the relevant individual (D and PS) stresses and control (C). Indicated are the AGI gene locus ID and the commonly used abbreviation if available (otherwise indicates as NA). The color scale indicates the expression levels; yellow represents up- and blue represents down-regulation.

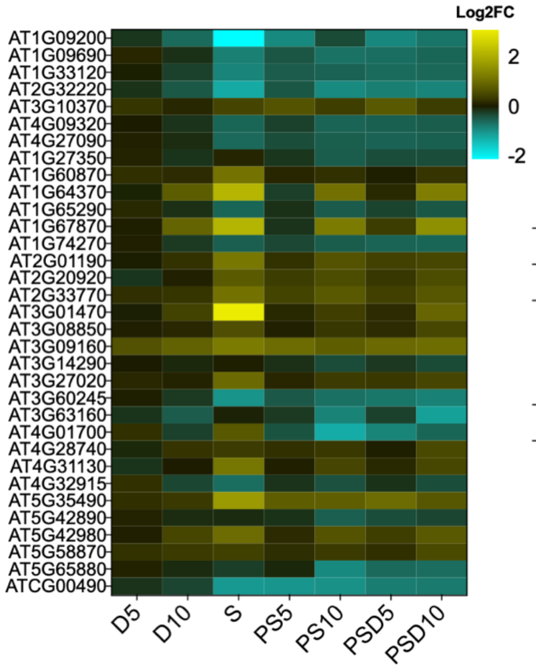

**Category**	KEGG
**Term**	ath03010
**Description**	Ribosome - Arabidopsis thaliana (thale cress)
**-Log$_{10}$P**	5.13612

Supplementary Figure S17. Relative expression values of 33 DEGs regulated by both PS and PSD. For each DEG, the AGI gene locus ID is indicated. Color scales indicate Log$_2$FC value (relative to control (C) conditions); yellow and blue indicate up- and down- regulations, respectively. The GO and KEGG enrichment analysis of the 33 DEGs are indicated in the table.

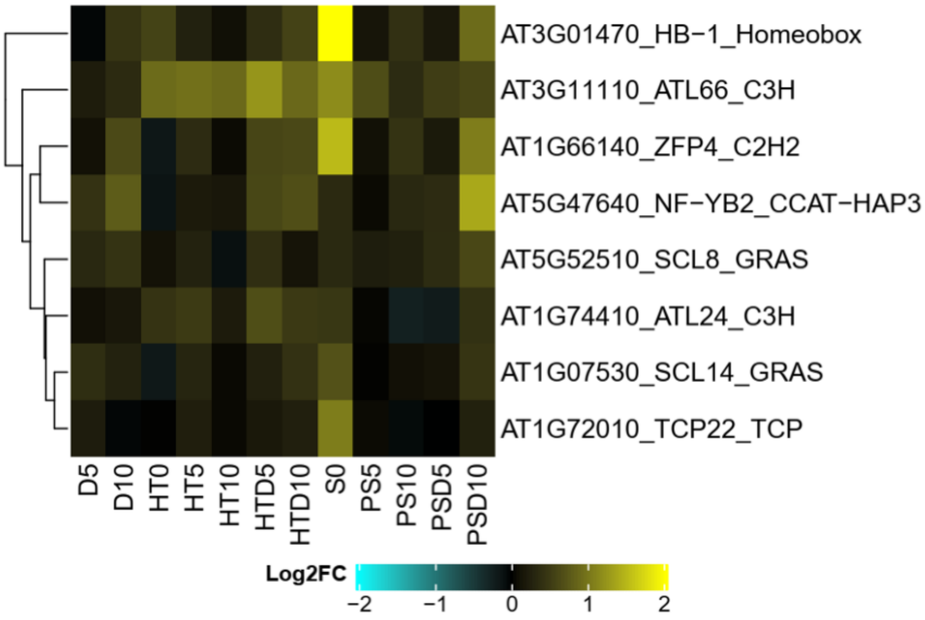

Supplementary Figure S18. Relative expression values of 8 TFs that were upregulated in both combined (HTD) and sequential (PSD) stresses, compared to controls (C). Indicated are the AGI gene locus ID and the commonly used abbreviation. Color scales indicate Log_2FC value (relative to control (C) conditions); yellow and blue indicate up- and down- regulations, respectively.

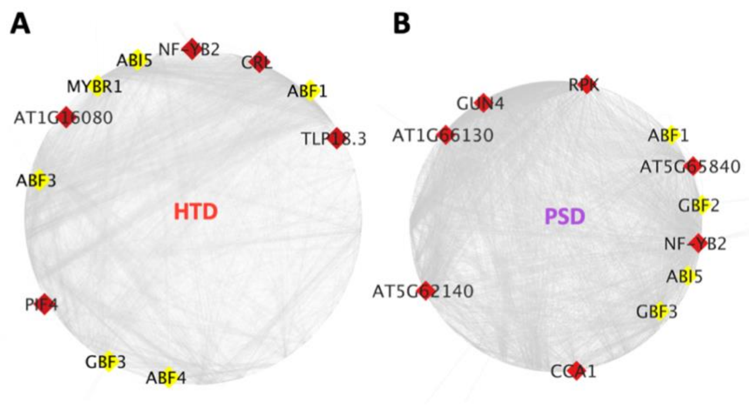

Gene Name	Locus ID
GBF3	AT2G46270
ABF1	AT1G49720
ABI5	AT2G36270
NF-YB2	AT5G47640

Supplementary Figure S19. Gene regulatory network (GRNs) for DEGs from the upregulated gene clusters in combined high temperature and drought (HTD) and submergence followed by drought (PSD). For each GRN, the putative upstream regulators (11) with the greatest number of connections with the others in the same network are indicated. Indicated are GRNs for DEGs from the upregulated gene clusters in **(A)** combined high temperature and drought (HTD) and **(B)** submergence followed by drought (PSD). Genes associated with ABA responses are highlighted with yellow nodes and the common highlighted regulators shared by the two GRNs are indicated in the table on the right (Gene name and AGI locus ID).

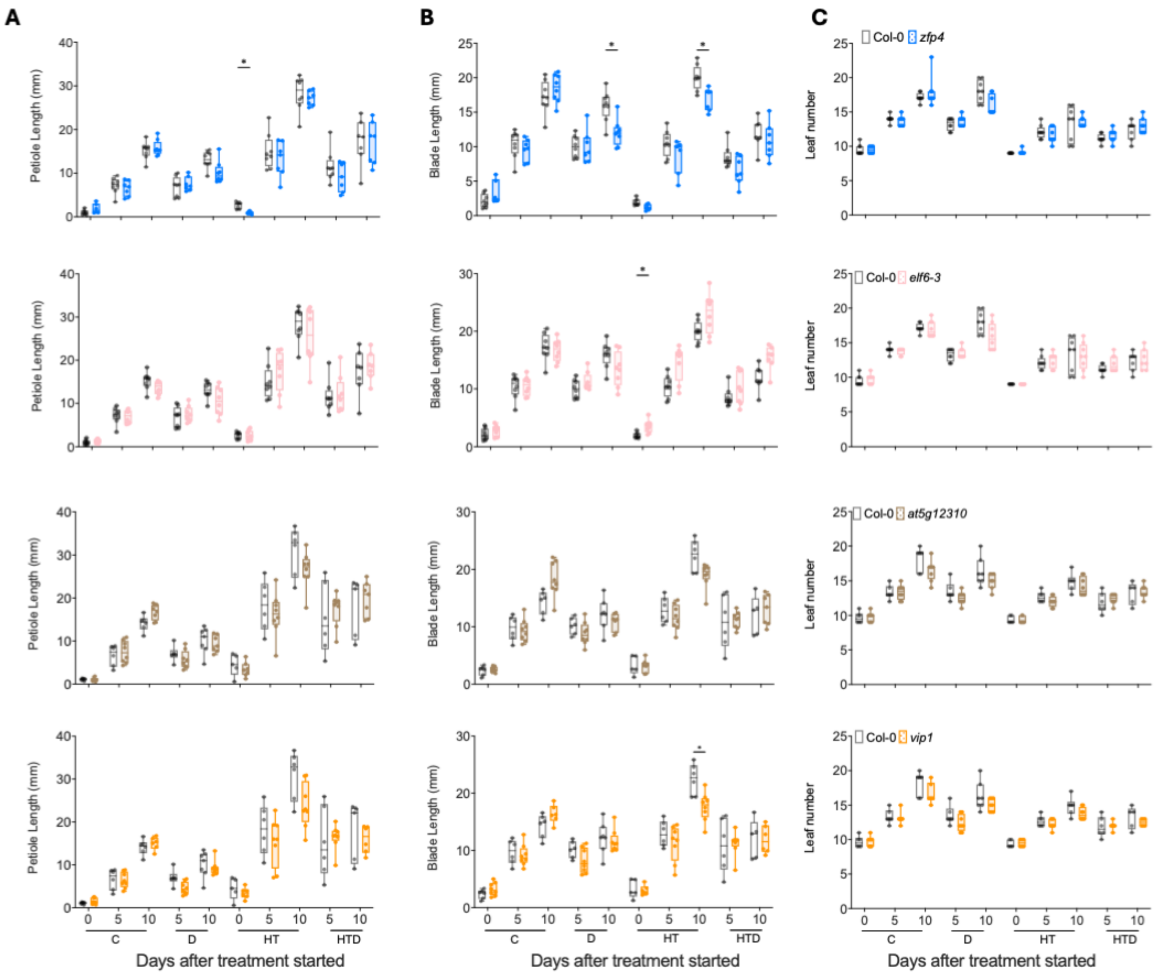

Supplementary Figure S20. Effect of combined and individual stresses on leaf development traits of selected mutants. **(A, B, C)** Average lengths of petiole (A) and blade (B) of young leaves, and leaf number (C) of Arabidopsis mutants (*zfp4* (blue), *elf6-3* (pink), *at5g12310* (brown), *vip1* (orange) and wild-type plants (Col-0; grey). Box plots show the median and boxes indicate boundaries of the second and third quartiles (Q1 and Q3) of the data distribution. Whiskers indicate Q1 and Q4 values within 1.5 times the interquartile range. Asterisks represent significant differences between the mutant and the corresponding wild-type plants within the same timepoint ($p < 0.05$, multiple unpaired t-test with Holm-Šídák correction). Numbers indicates days after treatment started. For treatment abbreviations see legend of Supplementary Figure S1. n = 5-10.

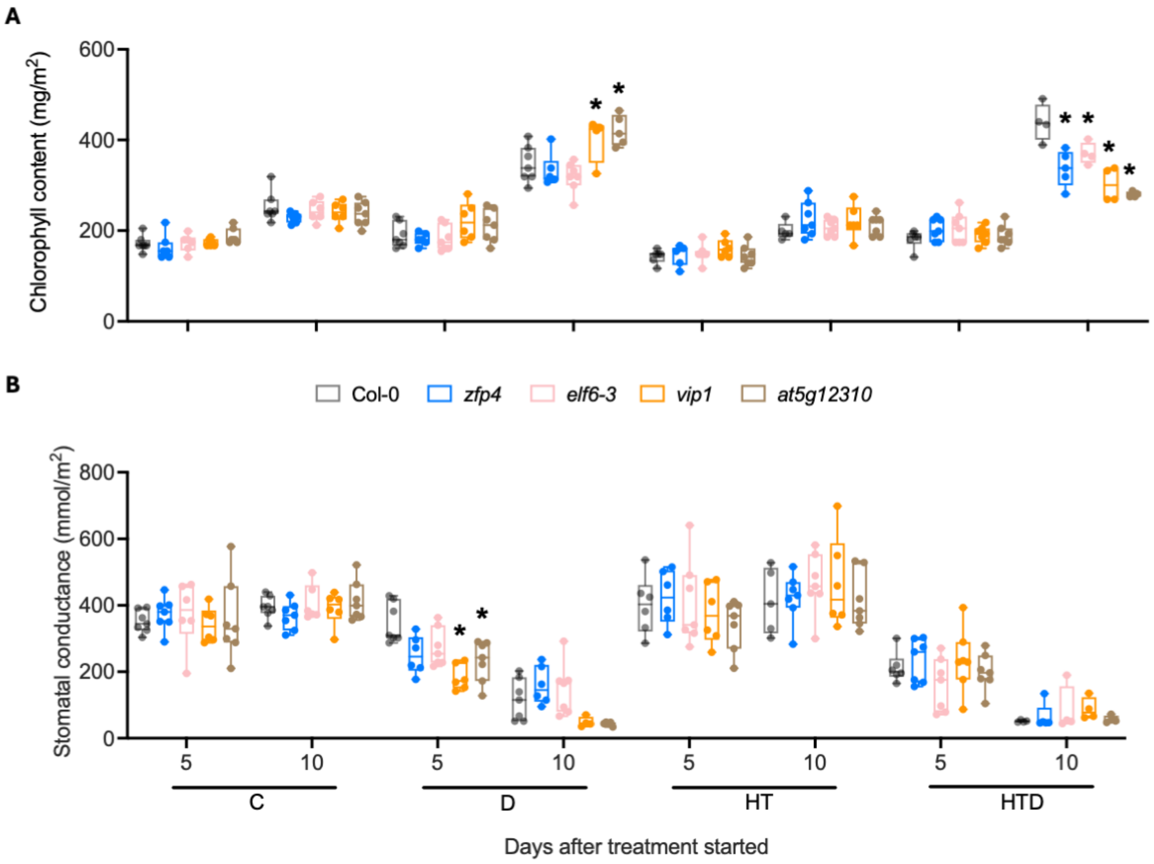

Supplementary Figure S21. Effect of combined and individual stresses on chlorophyll content and stomatal conductance of selected mutants and corresponding wild-type. **(A, B)** chlorophyll content (A) and stomatal conductance (B) of Arabidopsis mutants (*zfp4* (blue), *elf6-3* (pink), *at5g12310* (brown), *vip1* (orange) and *pif4-2* (red)) and the wild-type (Col-0). Boxes indicate boundaries of the second and third quartiles (Q) of the data distribution. Horizontal bars indicate median and whiskers Q1 and Q4 values within 1.5 times the interquartile range. Asterisks represent significant differences between mutant and the corresponding wild-type within the same timepoint ($p < 0.05$, one-way ANOVA with Dunnett test). Numbers indicate days after treatments started. For treatment abbreviations see legend of Supplementary Figure S1. n = 4-7.

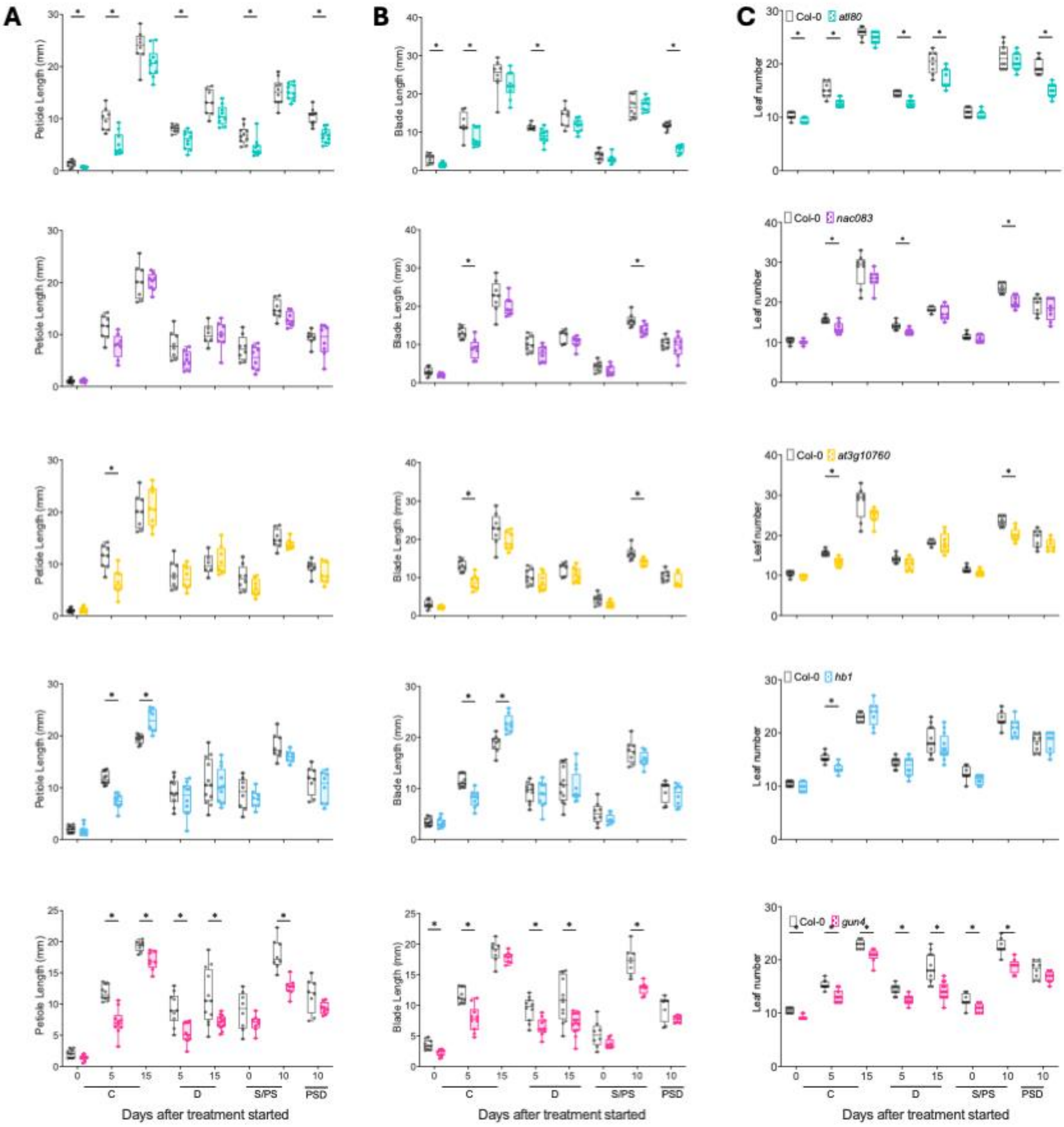

Supplementary Figure S22. Effect of sequential and individual stresses on leaf development of selected mutants. **(A, B, C)** Average lengths of petiole (A) and blade (B) of young leaves, and (C) leaf number of Arabidopsis mutants (*atl80* (aqua), *nac083* (purple), *at3g10760* (yellow), *hb1* (azure) and *gun4* (magenta)) and wild-type plants (Col-0; grey). Box plots show the median and boxes indicate boundaries of the second and third quartiles (Q1 and Q3) of the data distribution. Whiskers indicate Q1 and Q4 values within 1.5 times the interquartile range. Asterisks represent significant differences between the mutant and the corresponding wild-type plants within the same timepoint ($p < 0.05$, multiple unpaired t-test with Holm-Šídák correction). Numbers indicates days after treatment started. For treatment abbreviations see legend of Supplementary Figure S1. n = 6-13.

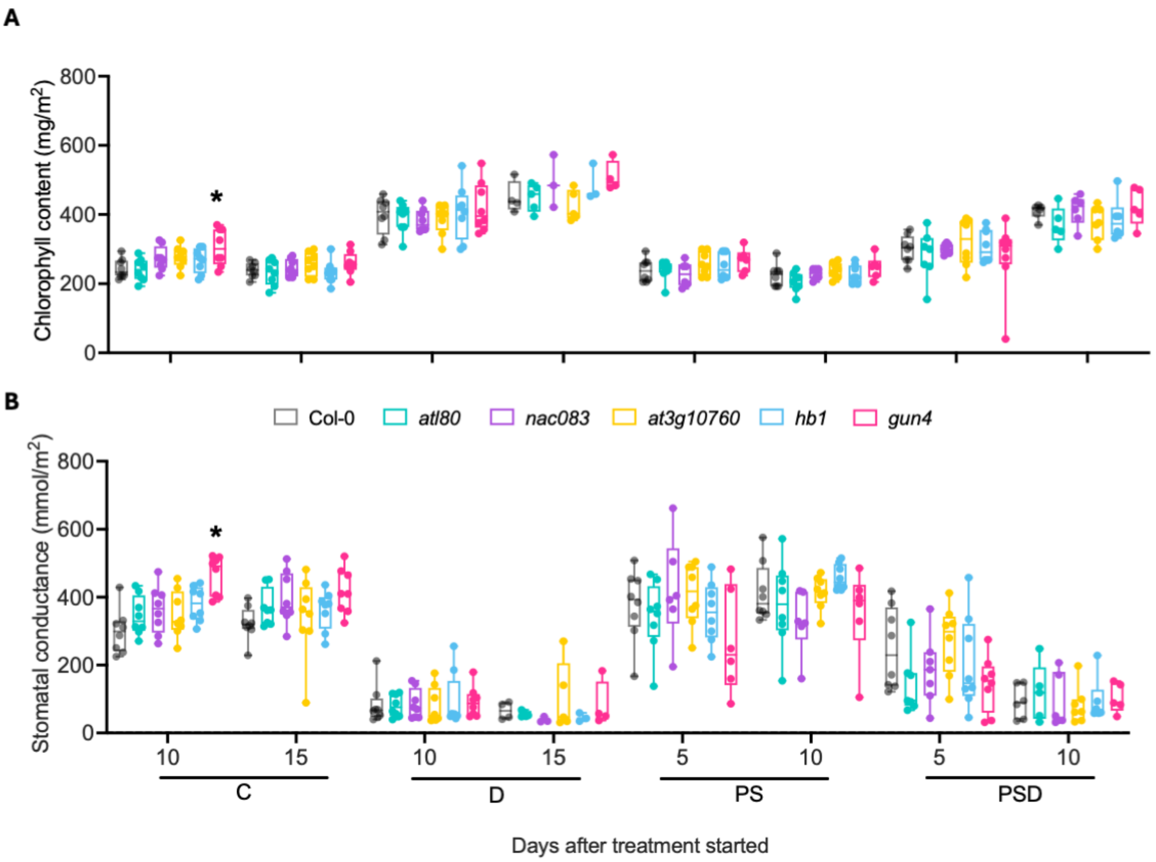

Supplementary Figure S23. Effect of sequential and individual stresses on chlorophyll content and stomatal conductance of selected mutants and corresponding wild-type. **(A, B)** chlorophyll content (A) and stomatal conductance (B) of Arabidopsis mutants (*atl80* (aqua), *nac083* (purple), *at3g10760* (yellow), *hb1* (azure) and *gun4* (magenta)) and the wild-type plants (Col-0; gray). Boxes indicate boundaries of the second and third quartiles (Q) of the data distribution. Horizontal bars indicate median and whiskers Q1 and Q4 values within 1.5 times the interquartile range. Asterisks represent significant differences between the mutant and the corresponding wild-type plants within the same time point ($p < 0.05$, one-way ANOVA with Dunnett test). Numbers indicate days after treatment started. For treatment abbreviations see legend of Supplementary Figure S1. n = 3-8.

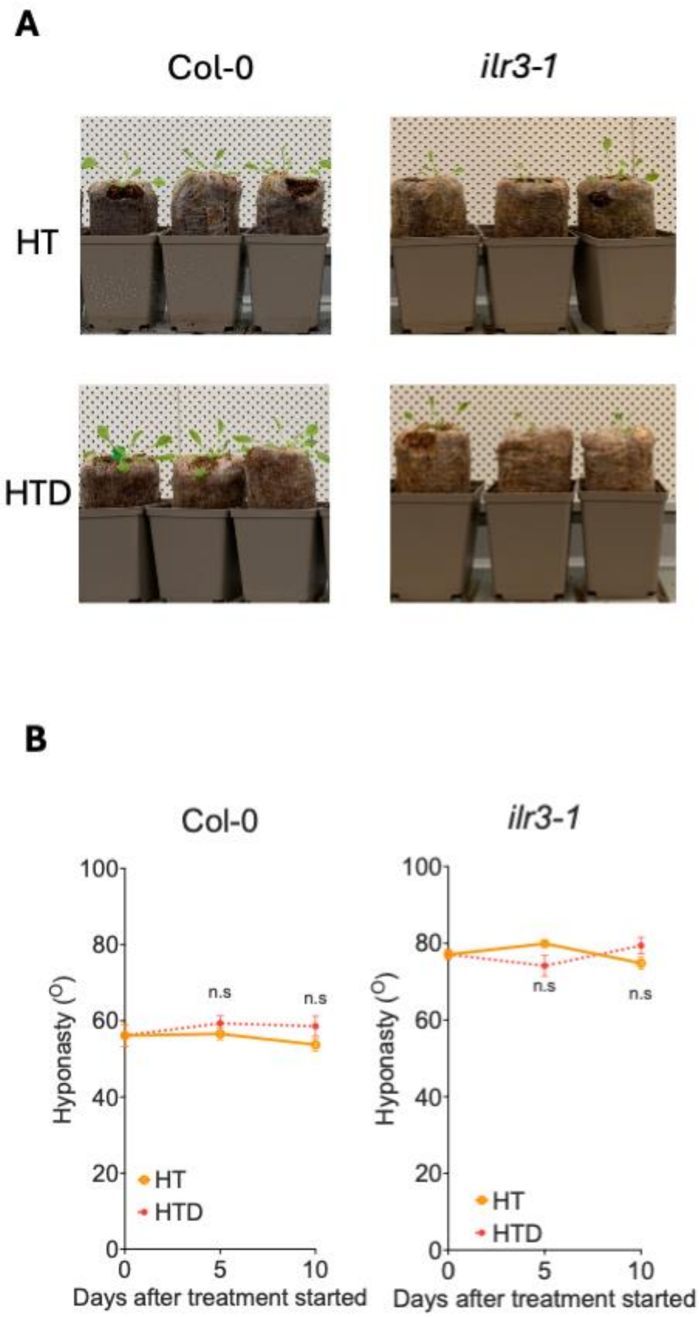

Supplementary Figure S24. Typical enhanced leaf hyponasty phenotype of *ilr3* compared to Col-0 wild-type plants during subjection to high temperature treatments. **(A)** Representative rosette (side) images of Col-0 (left), *ilr3-1* (right) upon 5-day high temperature (HT, upper row) or combined high temperature and drought (HTD, lower row) treatment. **(B)** Average angles of the 2 most hyponastic leaves of individual plants, relative to the horizontal. Red dashed lines represent HTD treatment and solid orange lines indicate HT treatment. The letter 'n.s' represents no significant differences between treatments within the same timepoint ($p>0.05$, unpaired t-test). For treatment abbreviations and used colors, see legend of Supplementary Figure S1. n = 5-10.

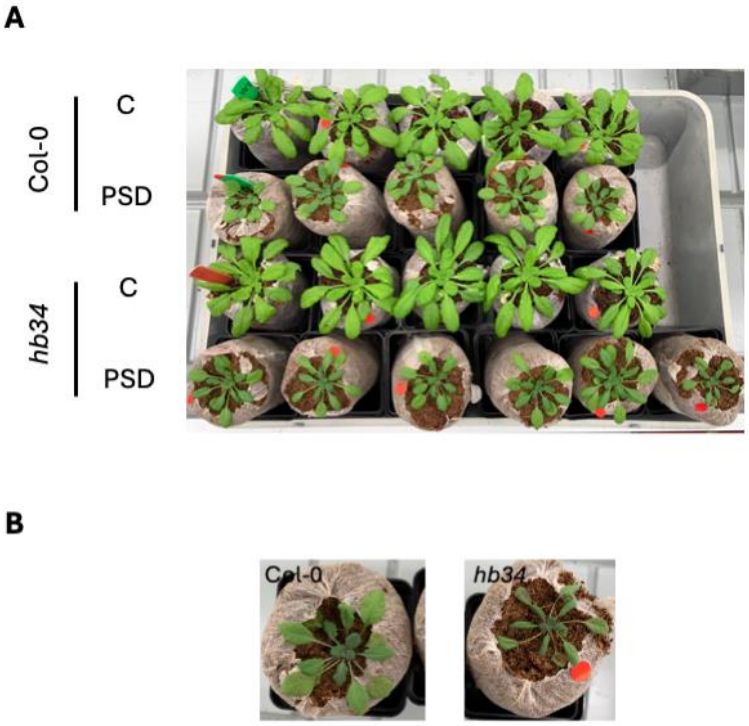

Supplementary Figure S25. Representative images of Col-0 wild-type and *hb34* mutant subjected to control and submergence followed by drought (PSD). Images are from Col-0 (upper two rows) and *hb34* mutant (lower two rows) plants on Jiffy 7c coconut pellet growth substrate, subjected to control (C; 1st and 3rd row) and submergence followed by drought (PSD; 2nd and 4th row) at **(A)** 15 days after treatments started and **(B)** the day of wilting.

Supplementary References

- Aguilar Martinez, J.A., Sinha, N.R., 2013. Analysis of the role of Arabidopsis class I TCP genes AtTCP7, AtTCP8, AtTCP22, and AtTCP23 in leaf development. Frontiers in Plant Science 4, 406.
- Ansari, M.I., Lin, T.-P., 2011. Arabidopsis thaliana thylakoid lumen 18.3 protein (TLP 18.3) gene regulate developmental process. CB 1, 17–21.
- Capella, M., Ribone, P.A., Arce, A.L., Chan, R.L., 2015. *Arabidopsis thaliana* HomeoBox 1 (At HB 1), a Homedomain-Leucine Zipper I (HD -Zip I) transcription factor, is regulated by PHYTOCHROME-INTERACTING FACTOR 1 to promote hypocotyl elongation. New Phytol 207, 669–682. https://doi.org/10.1111/nph.13401
- Cappadocia, L., Maréchal, A., Parent, J.-S., Lepage, É., Sygusch, J., Brisson, N., 2010. Crystal Structures of DNA-Whirly Complexes and Their Role in *Arabidopsis* Organelle Genome Repair. The Plant Cell 22, 1849–1867. https://doi.org/10.1105/tpc.109.071399
- Chiang, Y.-H., Zubo, Y.O., Tapken, W., Kim, H.J., Lavanway, A.M., Howard, L., Pilon, M., Kieber, J.J., Schaller, G.E., 2012. Functional Characterization of the GATA Transcription Factors GNC and CGA1 Reveals Their Key Role in Chloroplast Development, Growth, and Division in Arabidopsis. Plant Physiology 160, 332–348. https://doi.org/10.1104/pp.112.198705
- Cifuentes-Esquivel, N., Celiz-Balboa, J., Henriquez-Valencia, C., Mitina, I., Arraño-Salinas, P., Moreno, A.A., Meneses, C., Blanco-Herrera, F., Orellana, A., 2018. bZIP17 regulates the expression of genes related to seed storage and germination, reducing seed susceptibility to osmotic stress. J of Cellular Biochemistry 119, 6857–6868. https://doi.org/10.1002/jcb.26882
- Corrales, A., Carrillo, L., Lasierra, P., Nebauer, S.G., Dominguez-Figueroa, J., Renau-Morata, B., Pollmann, S., Granell, A., Molina, R., Vicente-Carbajosa, J., Medina, J., 2017. Multifaceted role of cycling DOF factor 3 (CDF3) in the regulation of flowering time and abiotic stress responses in *Arabidopsis*. Plant Cell & Environment 40, 748–764. https://doi.org/10.1111/pce.12894

- Fode, B., Siemsen, T., Thurow, C., Weigel, R., Gatz, C., 2008. The *Arabidopsis* GRAS Protein SCL14 Interacts with Class II TGA Transcription Factors and Is Essential for the Activation of Stress-Inducible Promoters. The Plant Cell 20, 3122–3135. https://doi.org/10.1105/tpc.108.058974
- Fölsche, V., Großmann, C., Richter, A.S., 2022. Impact of Porphyrin Binding to GENOMES UNCOUPLED 4 on Tetrapyrrole Biosynthesis in planta. Front. Plant Sci. 13, 850504. https://doi.org/10.3389/fpls.2022.850504
- Frerigmann, H., Gigolashvili, T., 2014. MYB34, MYB51, and MYB122 Distinctly Regulate Indolic Glucosinolate Biosynthesis in Arabidopsis thaliana. Molecular Plant 7, 814–828. https://doi.org/10.1093/mp/ssu004
- Gao, H., Brandizzi, F., Benning, C., Larkin, R.M., 2008. A membrane-tethered transcription factor defines a branch of the heat stress response in *Arabidopsis thaliana*. Proc. Natl. Acad. Sci. U.S.A. 105, 16398–16403. https://doi.org/10.1073/pnas.0808463105
- Joseph, M.P., Papdi, C., Kozma-Bognár, L., Nagy, I., López-Carbonell, M., Rigó, G., Koncz, C., Szabados, L., 2014. The Arabidopsis ZINC FINGER PROTEIN3 Interferes with Abscisic Acid and Light Signaling in Seed Germination and Plant Development. Plant Physiology 165, 1203–1220. https://doi.org/10.1104/pp.113.234294
- Keyzor, C., Mermaz, B., Trigazis, E., Jo, S., Song, J., 2021. Histone Demethylases ELF6 and JMJ13 Antagonistically Regulate Self-Fertility in Arabidopsis. Front. Plant Sci. 12, 640135. https://doi.org/10.3389/fpls.2021.640135
- Lee, Y.K., Kumari, S., Olson, A., Hauser, F., Ware, D., 2022. Role of a ZF-HD Transcription Factor in miR157-Mediated Feed-Forward Regulatory Module That Determines Plant Architecture in Arabidopsis. IJMS 23, 8665. https://doi.org/10.3390/ijms23158665
- Lobbes, D., Rallapalli, G., Schmidt, D.D., Martin, C., Clarke, J., 2006. SERRATE: a new player on the plant microRNA scene. EMBO Reports 7, 1052–1058. https://doi.org/10.1038/sj.embor.7400806
- Nambara, E., Suzuki, M., Abrams, S., McCarty, D.R., Kamiya, Y., McCourt, P., 2002. A Screen for Genes That Function in Abscisic Acid Signaling in *Arabidopsis thaliana*. Genetics 161, 1247–1255. https://doi.org/10.1093/genetics/161.3.1247

- Rampey, R.A., Woodward, A.W., Hobbs, B.N., Tierney, M.P., Lahner, B., Salt, D.E., Bartel, B., 2006. An Arabidopsis Basic Helix-Loop-Helix Leucine Zipper Protein Modulates Metal Homeostasis and Auxin Conjugate Responsiveness. Genetics 174, 1841–1857. https://doi.org/10.1534/genetics.106.061044
- Sato, H., Suzuki, T., Takahashi, F., Shinozaki, K., Yamaguchi-Shinozaki, K., 2019. NF-YB2 and NF-YB3 Have Functionally Diverged and Differentially Induce Drought and Heat Stress-Specific Genes. Plant Physiol. 180, 1677–1690. https://doi.org/10.1104/pp.19.00391
- Stamm, P., Ravindran, P., Mohanty, B., Tan, E.L., Yu, H., Kumar, P.P., 2012. Insights into the molecular mechanism of RGL2-mediated inhibition of seed germination in Arabidopsis thaliana. BMC Plant Biol 12, 179. https://doi.org/10.1186/1471-2229-12-179
- Sun, Le, Lu, S.-J., Zhang, S.-S., Zhou, S.-F., Sun, Ling, Liu, J.-X., 2013. The Lumen-Facing Domain Is Important for the Biological Function and Organelle-to-Organelle Movement of bZIP28 during ER Stress in Arabidopsis. Molecular Plant 6, 1605–1615. https://doi.org/10.1093/mp/sst059
- Tissot, N., Robe, K., Gao, F., Grant-Grant, S., Boucherez, J., Bellegarde, F., Maghiaoui, A., Marcelin, R., Izquierdo, E., Benhamed, M., Martin, A., Vignols, F., Roschzttardtz, H., Gaymard, F., Briat, J., Dubos, C., 2019. Transcriptional integration of the responses to iron availability in Arabidopsis by the bHLH factor ILR3. New Phytologist 223, 1433–1446. https://doi.org/10.1111/nph.15753
- Yang, S.-D., Seo, P.J., Yoon, H.-K., Park, C.-M., 2011. The *Arabidopsis* NAC Transcription Factor VNI2 Integrates Abscisic Acid Signals into Leaf Senescence via the *COR* / *RD* Genes. The Plant Cell 23, 2155–2168. https://doi.org/10.1105/tpc.111.084913
- Yu, X., Li, Li, Li, Lei, Guo, M., Chory, J., Yin, Y., 2008. Modulation of brassinosteroid-regulated gene expression by jumonji domain-containing proteins ELF6 and REF6 in *Arabidopsis*. Proc. Natl. Acad. Sci. U.S.A. 105, 7618–7623. https://doi.org/10.1073/pnas.0802254105
- Zhao, H., Nie, K., Zhou, H., Yan, X., Zhan, Q., Zheng, Y., Song, C., 2020. ABI5 modulates seed germination via feedback regulation of the expression of the

PYR/PYL/RCAR ABA receptor genes. New Phytol 228, 596–608. https://doi.org/10.1111/nph.16713

- Zhao, Q., Li, M., Jia, Z., Liu, F., Ma, H., Huang, Y., Song, S., 2016. AtMYB44 Positively Regulates the Enhanced Elongation of Primary Roots Induced by *N* -3-Oxo-Hexanoyl-Homoserine Lactone in *Arabidopsis thaliana*. MPMI 29, 774–785. https://doi.org/10.1094/MPMI-03-16-0063-R
- Zhao, Y., Jiang, T., Li, L., Zhang, X., Yang, T., Liu, C., Chu, J., Zheng, B., 2021. The chromatin remodeling complex imitation of switch controls stamen filament elongation by promoting jasmonic acid biosynthesis in Arabidopsis. Journal of Genetics and Genomics 48, 123–133. https://doi.org/10.1016/j.jgg.2021.02.003
- Ziemer, P., 2008. Die Bedeutung der GRAS-Proteine für die Entwicklung von Pflanzen untersucht am Modellorganismus Arabidopsis thaliana. Dissertation, LMU München: Faculty of Biology. https://doi.org/10.5282/edoc.9572
