# Supplementary material for: Deciphering acclimation to sublethal combined and sequential abiotic stresses in Arabidopsis thaliana
Source: Plant Physiol. 2024 Oct 29;200(3):kiae581. doi: 10.1093/plphys/kiae581 (PMC13016629; doi:10.1093/plphys/kiae581)
Supplement: kiae581_Supplementary_Data [file pp.00944.2024-s06.pdf › pp.00944.2024-s06.pdf]

Plant Physiology®

# Deciphering Arabidopsis thaliana Acclimation to Sublethal Combined and Sequential Abiotic Stresses

Zhang Jiang, Ava Verhoeven, Yihong Li, Romy Geertsma, Rashmi Sasidharan, and Martijn Van Zanten

**Review Timeline:**

| | |
|---|---|
| Submission Date: | 13-Jun-2024 |
| Editorial Decision: | 26-Jul-2024 |
| Revision Received: | 06-Sep-2024 |
| Accepted: | 10-Oct-2024 |

26-Jul-2024

Dr. Martijn Van Zanten
Universiteit Utrecht
Molecular Plant Physiology
Utrecht 3584 CH
Netherlands

RE: Deciphering Arabidopsis thaliana Acclimation to Sublethal Combined Abiotic Stresses

Dear Dr. Van Zanten:

We have received reviews of your manuscript, which you may view at the end of this letter. On the basis of the advice received and my own evaluation, I believe that, pending revision, this article may ultimately be acceptable for publication in Plant Physiology.

Both reviewers thought your manuscript is an exciting contribution to the field and commend the amount of work presented here. They did, however, point out a few issues that I agree will improve the manuscript once addressed. The comments are quite detailed and from what I can see do not require further experiments, so they should be quite straight forward to address. In particular I would like to highlight point 1 from Reviewer 1 as something important to consider.

When you are ready to submit your revision, click the below link and find the red arrow under your Author Tasks heading for this revision submission.

If you want to visit this version of the manuscript, you may click the link below and then click on the folder that reads "Post Decision Manuscripts".

Link Not Available

When uploading the revised version of this article, please be sure to include as complete an answer as possible to the referees' criticisms during the first round of reviews in the "response to reviewers" section.

Plant Physiology considers a revision for up to 60 days after a manuscript has been provisionally accepted; limited extension of this timeframe is at the discretion of the Monitoring Editor. A reminder notice will be sent to you 2 weeks before the deadline, at which time you may choose to contact the editorial office to request an extension; if so, please provide a justification for your request. After the expiration of this deadline, the article would need to be submitted as a resubmission and would be handled as such.

Thank you very much for giving us an opportunity to review this work. I look forward to receiving the next version.

Sincerely,

Editor
Plant Physiology

----

Want to add this revision deadline to your calendar? Click below!

---------------------------------------------------------------------------- Reviewer comments:
Reviewer #1 (Comments for the Author):

Deciphering Arabidopsis thaliana acclimation to sublethal combined abiotic stress

In this article, Jiang and colleagues seek to describe the underlying transcriptional networks which govern the Arabidopsis response to; drought and heat, and submergence followed by drought.

The authors extensively characterise the physiological response of Arabidopsis to these multiple stresses, and establish an effective framework to measure gene expression using RNA sequencing. They show that plants exhibit a distinct transcriptional response to multiple stresses compared to single stresses, and that for both HTD and PSD conditions, responses cluster into four distinct subgroups controlling different biological aspects of the response. Their analysis reveals ELF6 and ATL80 as putative regulators of multiple stresses in Arabidopsis thaliana, which they confirm by phenotyping TDNA knockout lines.

The manuscript is well written and I applaud the effort of the authors- a tremendous amount of work has been undertaken to produce these data. I have here some suggestions which I believe will improve their study.

Major points:

1) Figure 4C,F, lines 194 - Can the authors confirm that the genes used for GO term enrichment, and subsequent analysis, are indeed differentially expressed? Figure 4C and F show the expression of 'differentially expressed' genes, but in all cases there are points sitting at Log2FC = 0. The figure legend states '|Log2FC| > 0' as defining differential expression. This is not sufficient and would not effectively capture a transcriptional response accurately, as even variation between biological replicates would yield gene expression values of '|Log2FC| > 0', even if an adjusted pValue was somehow < 0.05. Could the authors please clarify? My suggestion would be to implement a threshold cut off of at least '|Log2FC| > 0.58' which would reflect a change of at least 1.5 fold. This would likely impact the outcome of the clustering, GO term enrichment, and GRN analysis.

2) Could the authors please comment on their choice to use young leaves for their study and focus on day 5 and 10? Particularly when their phenotyping shows that;

- the chlorophyll content of young leaves at 5 and 10 days of drought show no difference to control plants (Fig S4B)
- petiole length is unchanged (S5B) in response to drought
- blade length is unchanged (S5C) in response to drought
- water content is unchanged (S7A) in response to drought
- after 10 days of drought, the water content of whole rosettes was unaffected (S12A)

The age of the tissues appears to have impacted their findings, as they observe almost no DEGs in their drought treated plants. This suggests their drought treatment is having little to no effect on the young leaves, and therefore their conclusions around the impact of dual stress on plant transcriptional responses should be less strong. Whilst acknowledged in the discussion, the cited paper (Prasch & Sonnewald) harvested 'leaves from five to eight plants which were grouped for one biological replicate'. I am therefore unsure the drought treatment has worked as would typically be expected.

3) How the authors constructed the gene regulatory networks and the identification of the 11 nodes regulating multi-stress response are not mentioned in the manuscript. Please include these.

4) Figure 3: Whilst the authors show clear physiological differential responses to multi-stress in their knock out mutants, do these possess phenotypes under single stresses? The importance of these genes in coordinating multi-stress responses might also be evaluated by looking at survival rates in response to HTD and PSD, but also D and S.

5) Figures 3B, 3D, 6C, 6D - would the authors please consider adjusting the colours of these heatmaps to reflect the data they present? The data in 3B for instance is skewed toward values between 1 and 2. Because the scale goes up to 4, this makes it very hard to evaluate the magnitude of differential responses. Furthermore, could the authors make it clear in the figure legend that positive values (derived by comparing to Col-0) indicate stronger or weaker responses, as this is not clear. For instance, gun4 (Fig 3D) has a response that is blue (close to 0) but this is somehow statistically significantly different to WT?

Minor points:

The authors might consider altering their title to 'Deciphering Arabidopsis thaliana acclimation to sublethal combined and sequential abiotic stresses', as the PSD condition is not combined.

Line 267 - Of the 29 mutants with a putative...
Figure 4B, C, 5B,C - Would the authors consider reformatting these figures (perhaps by moving one or two of their targets to the supplement, or displaying less timepoints) as they are too small and the resolution is poor when zoomed in.
Line 300 - Under HTD, not In HTD
Fig 4C zpf4 legend (top right) has a typo and should be zfp4
Line 440 and Figure 2B - could the authors comment in the discussion on why the HTD transcriptional response seems to repress the HT response? 233 genes are no longer differentially expressed suggesting the addition of drought specifically represses the HT transcriptional response.
Line 81 - EARLY flowering 6 (EARLY is missing)

Line 250 - The data being referred to is in figure S19, not S20.
Line 239, 385, 420 - Please be clear throughout the manuscript when talking about the PSD condition that this is sequential and not combined.
Line 401 - I am not convinced of the rationale that HT was dominant over D because plants had been subject to HT for much longer. If anything, this would suggest the opposite, as plants would have become adapted to the high temperature. Please consider revising this conclusion, or supplying further clarification.
Line 414 - 'the observed inhibition of growth and development could be explained by hampered photosynthetic acclimation'. The data presented do not support this conclusion; figure 2F suggests photosynthesis is upregulated in response to drought, and figure S4 shows an increase in chlorophyll content of young leaves in response to drought.
454 - Consider including in the body text the number of downregulated genes not considered for HTD and PSD for context. It might also be worth mentioning in the results section why the downregulated genes were not analysed.
In the interest of transparency, please consider formatting the supplemental tables in .xlsx or .csv files rather than having them embedded in a .pdf - in their current format downstream use of the data by the community virtually impossible.

Reviewer #2 (Comments for the Author):

This is an excellent study of the response of Arabidopsis to sublethal abiotic stress combination. The authors used two different types of combinations (drought and heat and submergence followed by drought), conducted RNA-Seq and phenotypic characterization, and tested over 40 mutants based on their findings. Several of these mutants demonstrated a phenotype identifying novel genes with a potential to impact plant acclimation to abiotic stress combination. I find this manuscript to be novel and of high level and importance, and I would like the authors to answer the following points before it could be published:

Line 89 - (and above) The authors refer to additive effects. Perhaps indicate that the additive effects are mainly negative.

Lines 89 - In this paragraph, please indicate the length of time for submergence in the text.

Line 164 - I find it interesting that the authors detected viral RNA at such levels in their samples and I comment them for reporting/addressing this. I never look for these sequences in my RNA-Seq data, but I will start from now. I hope others will also look for pathogen-related sequences in their RNA-Seq data, as pathogens will impact abiotic stress responses. I am happy with the controls the authors did for this matter.

Figure 2 - The transcriptomic response of plants to drought was very minimal. Please refer to it in the text with more explanations.

Line 242 - Why not show the results of the GRN analysis in the manuscript (now only shown in supplemental material)? Also please add some text in this section about what/how the GRN was performed.

Regarding mutant analysis, was this done with 2 independent alleles for each gene? I would like to see an analysis of 2 independent alleles at least for the few genes the authors mentioned in the abstract, but maybe the editor would like to see for more.

There is no mention as to how the GRN was done in the method section. Please add more information on this.

Regarding the discussion, Zandalinas et al 2021 [Zandalinas SI, Sengupta S, Fritschi FB, Azad RK, Nechushtai R, Mittler R. (2021) The impact of multifactorial stress combination on plant growth and survival. New Phytol. 230(3):1034-1048.] used sublethal stress levels to study the response of Arabidopsis to multifactorial stress combination identifying similar additive effects. Please add this work to your discussion.

**Reviewer #1**

In this article, Jiang and colleagues seek to describe the underlying transcriptional networks which govern the Arabidopsis response to; drought and heat, and submergence followed by drought.

The authors extensively characterise the physiological response of Arabidopsis to these multiple stresses, and establish an effective framework to measure gene expression using RNA sequencing. They show that plants exhibit a distinct transcriptional response to multiple stresses compared to single stresses, and that for both HTD and PSD conditions, responses cluster into four distinct subgroups controlling different biological aspects of the response. Their analysis reveals ELF6 and ATL80 as putative regulators of multiple stresses in Arabidopsis thaliana, which they confirm by phenotyping TDNA knockout lines.

The manuscript is well written and I applaud the effort of the authors- a tremendous amount of work has been undertaken to produce these data. I have here some suggestions which I believe will improve their study.

**Major points**:

1) Figure 4C,F, lines 194 - Can the authors confirm that the genes used for GO term enrichment, and subsequent analysis, are indeed differentially expressed? Figure 4C and F show the expression of 'differentially expressed' genes, but in all cases there are points sitting at Log2FC = 0. The figure legend states '|Log2FC| > 0' as defining differential expression. This is not sufficient and would not effectively capture a transcriptional response accurately, as even variation between biological replicates would yield gene expression values of '|Log2FC| > 0', even if an adjusted pValue was somehow < 0.05. Could the authors please clarify?
My suggestion would be to implement a threshold cut off of at least '|Log2FC| > 0.58' which would reflect a change of at least 1.5 fold. This would likely impact the outcome of the clustering, GO term enrichment, and GRN analysis.

>> **Response:** We believe the reviewer is referring to Figure 2, not Figure 4.
We confirm that the genes indicated in Figure 2A and B are differentially expressed, based on differences in (Log2-normalized) TPM counts (after Benjamini-Hochberg correction). In the selection of DEGs we did not take fold change (FC) into account, given the mild effects on the transcriptome.
To avoid confusion, we have now removed the mention of '|$Log_2$FC| > 0' from the legend. To further clarify we have also added the following statement to the materials and methods (lines 602-603): *"In the selection of DEGs we did not take fold change (FC) into account, given the mild effects of applied stresses on the transcriptome."*

The reason that in Figure 2C and F genes are also indicated at (or near) Log2FC = 0 is that in this figure, in each treatment (individual Violin plot), all genes are presented that are significantly regulated in at least one or more of the treatments, thus also including those genes that are not affected by the indicated treatment per se. To clarify this, we added the following remark to the legend: *"Note that in each treatment all genes are indicated that are significantly regulated in at least one or more of the treatments."*

Due to the mild severity of the applied stresses, we did not detect very strong transcriptomic responses. We therefore choose a relatively lenient significance threshold of p<0.05 in our analysis to capture a broad range of DEG candidate genes. The proposed inclusion of a FC

threshold (of 0.58) would lead to the loss of many prime candidates, including ELF6, which is of central importance to our study. We therefore do not favor including the proposed threshold (of 0.58), because it has major implications for the entire paper. At lines 179-180 we mention that using a lenient threshold was needed to detect DEGs by mentioning: *"In response to D, only 3 differentially expressed genes were detected, despite using a relatively lenient threshold (DEGs FDR < 0.05)."*

2) Could the authors please comment on their choice to use young leaves for their study and focus on day 5 and 10? Particularly when their phenotyping shows that;

- the chlorophyll content of young leaves at 5 and 10 days of drought show no difference to control plants (Fig S4B)
- petiole length is unchanged (S5B) in response to drought
- blade length is unchanged (S5C) in response to drought
- water content is unchanged (S7A) in response to drought
- after 10 days of drought, the water content of whole rosettes was unaffected (S12A)

The age of the tissues appears to have impacted their findings, as they observe almost no DEGs in their drought treated plants. This suggests their drought treatment is having little to no effect on the young leaves, and therefore their conclusions around the impact of dual stress on plant transcriptional responses should be less strong. Whilst acknowledged in the discussion, the cited paper (Prasch & Sonnewald) harvested 'leaves from five to eight plants which were grouped for one biological replicate'. I am therefore unsure the drought treatment has worked as would typically be expected.

>> **Response:** While some phenotypic parameters such as chlorophyll content and blade length indeed showed minimal changes to our mild and relatively short drought treatment, other established developmental and physiological markers of drought, such as primary root length (Figure S5D) and stomatal conductance (Figure 1E), exhibited clear and significant responses, confirming that drought was perceived by the plants. Moreover, under HTD, more unique DEGs were detected compared to single D or HT, which confirms that D treatment did affect the plants, despite having a subtle effect. We are therefore confident that the plants used in this study experienced mild drought as anticipated. We discuss the relative subtle effects in the discussion section (Line 441-444):
"*the young leaves that were sampled may be less responsive to drought than for instance roots tissues, as plants might evoke protective mechanisms to prioritize stress protection of younger leaves and meristematic tissues (Rankenberg et al., 2024, 2021; Verelst et al., 2013)".*

Our choice to focus on young leaves at days 5 and 10 after initiation of drought treatment was driven by the premise that these tissues actively engage in plant developmental acclimation (response of developing tissues) to HTD and PSD, rather than dynamic acclimation (response of fully developed tissues). Young leaves were therefore deemed more suitable as they were fully exposed to stresses while expanding. Although perhaps not so strongly responsive as for instance root tissues indeed, the effect of drought on shoots is relatively poorly studied. We therefore think that our work focusing on above-ground effects adds to the literature base on plant acclimation to drought.

3) How the authors constructed the gene regulatory networks and the identification of the 11 nodes regulating multi-stress response are not mentioned in the manuscript. Please include these.

>> **Response:** This comment is similar to point 5 and point 7 of Reviewer 2.

This was indeed missing. We now included a description of our gene regulatory network (GRN) construction in both the Materials and Methods (Line 614-616) and the Results (Line 241-243) sections of the revised manuscript. Briefly, we employed the TF2Network tool to infer GRNs from the RNA-Seq data, focusing on identifying putative upstream regulators and co-expressors associated with the upregulation of DEGs under HTD and PSD. DEGs from upregulated HTD or PSD clusters (Cluster 3 and 4) were inputted respectively to TF2Network tool for the construction of the GRNs. This tool integrates the information of both protein-DNA interactions and co-expression that are experimentally confirmed or computationally predicted in Arabidopsis. For the constructed GRNs, we selected the top 11 nodes with the highest number of connections with the others within the same GRN.

4) Figure 3: Whilst the authors show clear physiological differential responses to multi-stress in their knock out mutants, do these possess phenotypes under single stresses? The importance of these genes in coordinating multi-stress responses might also be evaluated by looking at survival rates in response to HTD and PSD, but also D and S.

>> **Response:** In our study, survival rates were only relevant under drought (D), high temperature combined with drought (HTD), and post-submergence followed by drought (PSD), as these conditions led to eventual plant mortality. The single stresses: submergence (S) under light conditions, post-submergence (PS), and high temperature (HT) at 27 °C did not result in plant death. Therefore, survival rates were not measured under these conditions. We now mention this in the discussion section (Line 465-467): *"Also, since the mild HT (at 27 °C), S (in the light) and PS treatments did not cause plant mortality, the survival related measurements were only conducted on plants exposed to HTD or PSD."*

Our initial screening of mutant lines aimed at identifying those mutants with the most pronounced phenotypic deviations from Col-0 (wild type) in response to combined HTD or sequential PSD. This approach allowed us to focus on mutants showing significant responses linked to acclimation and survival under these multi-stress conditions. Subsequently, only the most promising mutant lines, as identified through this screening, were subjected to detailed morphological and physiological characterization under both individual and combinatorial stress conditions (Figures 4-5, Figures S20-S23).

5) Figures 3B, 3D, 6C, 6D - would the authors please consider adjusting the colours of these heatmaps to reflect the data they present? The data in 3B for instance is skewed toward values between 1 and 2. Because the scale goes up to 4, this makes it very hard to evaluate the magnitude of differential responses. Furthermore, could the authors make it clear in the figure legend that positive values (derived by comparing to Col-0) indicate stronger or weaker responses, as this is not clear. For instance, *gun4* (Fig 3D) has a response that is blue (close to 0) but this is somehow statistically significantly different to WT?

>> **Response:** We agree that the previous color scales might have obscured subtle differences. In the revised figures, we have adjusted the color gradients to the yellow-cyan style and re-set the scales for a better reflection of the range of observed values. Additionally, we have also clarified the positive/negative effects indicated by the color of the heatmaps by adding the following statement to the figure legends of Figure 3 and Figure 6: "*Yellow and cyan indicate positive and negative effect, respectively, compared to Col-0.*"

**Minor points:**

6) The authors might consider altering their title to 'Deciphering Arabidopsis thaliana acclimation to sublethal combined and sequential abiotic stresses', as the PSD condition is not combined.

>> **Response:** We appreciate the suggestion and have amended the title as suggested to 'Deciphering *Arabidopsis thaliana* Acclimation to Sublethal Combined and Sequential Abiotic Stresses'.

7) Line 267 - Of the 29 mutants with a putative...

>> **Response:** Corrected as suggested (Line 272 in the revised manuscript); I.e. the 'a' has been added.

8) Figure 4B, C, 5B,C - Would the authors consider reformatting these figures (perhaps by moving one or two of their targets to the supplement, or displaying less timepoints) as they are too small and the resolution is poor when zoomed in.

>> **Response:** Figure 4 and 5 have been formatted to aid readability as requested.

9) Line 300 - Under HTD, not In HTD

>> **Response:** We have changed 'In HTD' to 'Under HTD' (Line 305 in the revised manuscript) as requested (and at line 115 as well).

10) Fig 4C zpf4 legend (top right) has a typo and should be zfp4

>> **Response:** Corrected as suggested.

11) Line 440 and Figure 2B - could the authors comment in the discussion on why the HTD transcriptional response seems to repress the HT response? 233 genes are no longer differentially expressed suggesting the addition of drought specifically represses the HT transcriptional response.

>> **Response:** We do not think that these 233 genes are "repressed" by drought. The combined or sequential stress should be viewed as a distinct condition that elicits a unique physiological and transcriptomic response in plants that cannot be directly compared to application of stress in isolation. The observed differences in gene expression between HT and HTD should be interpreted as a different reprogramming of the transcriptome under the combined stress condition, rather than as a 'simple' repression of the HT response by drought.
As requested, we added the following sentence to the discussion for clarification (lines 451-455 in the revised manuscript): *"We thus propose that changes in gene expression triggered by combined or sequential stresses should be interpreted as a different reprogramming of the*

*transcriptome as compared to the individual stresses, rather than as a simple tweaking of the single stress responses, despite some overlap in DEGs is noted."*

12) Line 81 - EARLY flowering 6 (EARLY is missing)

>> **Response:** Corrected as suggested.

13) Line 250 - The data being referred to is in figure S19, not S20.

>> **Response:** Corrected as suggested.

14) Line 239, 385, 420 - Please be clear throughout the manuscript when talking about the PSD condition that this is sequential and not combined.

>> **Response:** We have now clarified this, as requested, by adding the words 'combined' and 'sequential' to the HTD and PSD abbreviations at several places throughout the manuscript and figure legends.

15) Line 401 - I am not convinced of the rationale that HT was dominant over D because plants had been subject to HT for much longer. If anything, this would suggest the opposite, as plants would have become adapted to the high temperature. Please consider revising this conclusion, or supplying further clarification.

>> **Response:** We agree that using the term 'dominant' is indeed too strong/misleading. Therefore, we amended the text, as requested. We now write (lines 409-411); *"….in our study, HT had larger impact than D on plant rosette growth and leaf development (Fig. 1, B-D; Supplemental Figs. S5 and S6). This is likely because in our experiments HT was applied from germination onwards, well before D was applied."*
To avoid similar confusion, we also changed the word 'dominant' to 'stronger' in line 188 in the revised manuscript.

16) Line 414 - 'the observed inhibition of growth and development could be explained by hampered photosynthetic acclimation'. The data presented do not support this conclusion; figure 2F suggests photosynthesis is upregulated in response to drought, and figure S4 shows an increase in chlorophyll content of young leaves in response to drought.

>> **Response:** We agree with the reviewer that our claim was not fully supported by our data. We therefore removed the indicated sentence in the revised manuscript.

17) Consider including in the body text the number of downregulated genes not considered for HTD and PSD for context. It might also be worth mentioning in the results section why the downregulated genes were not analysed.

>> **Response:**
We added the number of downregulated DEGs, as requested, to the Result section by saying: *"The downregulated DEGs from Cluster 1 and 2 under HTD (836 DEGs) and PSD (504 DEGs) (Fig. 2) were not considered here."* (Line 263-264)

We revised the text in the Results section as requested to clarify the rationale for our candidate gene selection:

*"Because the validation was based on assessing mutants, only TFs that were upregulated during either combined HTD or sequential PSD, supplemented with some of the overrepresented genes from the GRNs, were considered in this study."* (Line 260-263).

For completeness, in the Discussion section we had already addressed the consequence of the omission of downregulated genes: *'This approach admittedly neglects downregulated genes, which might also play important roles in mediating responses to combined and/or sequential stresses and are worthwhile to test in future experiments.'* (Line 463-465).

18) In the interest of transparency, please consider formatting the supplemental tables in .xlsx or .csv files rather than having them embedded in a .pdf - in their current format downstream use of the data by the community virtually impossible.

\>> **Response:** All supplemental tables are now provided in .xlsx format to facilitate data accessibility for the research community, as requested.

---

**Reviewer #2 (Comments for the Author):**

This is an excellent study of the response of Arabidopsis to sublethal abiotic stress combination. The authors used two different types of combinations (drought and heat and submergence followed by drought), conducted RNA-Seq and phenotypic characterization, and tested over 40 mutants based on their findings. Several of these mutants demonstrated a phenotype identifying novel genes with a potential to impact plant acclimation to abiotic stress combination. I find this manuscript to be novel and of high level and importance, and I would like the authors to answer the following points before it could be published:

1) Line 89 - (and above) The authors refer to additive effects. Perhaps indicate that the additive effects are mainly negative.

\>> **Response:** As requested, we have clarified in the manuscript that the in general additive effects are indeed predominantly negative, emphasizing the compounded detrimental impacts on plant growth, development and physiology. (Line 25, 73 and 86 in the revised manuscript)

2) Lines 89 - In this paragraph, please indicate the length of time for submergence in the text.

\>> **Response:** As requested, the duration of submergence treatments has been added to this line (i.e. 5 days of submergence).

3) Line 164 - I find it interesting that the authors detected viral RNA at such levels in their samples and I comment them for reporting/addressing this. I never look for these sequences in my RNA-Seq data, but I will start from now. I hope others will also look for pathogen-related sequences in their RNA-Seq data, as pathogens will impact abiotic stress responses. I am happy with the controls the authors did for this matter.

\>> **Response:** Thank you for the compliment on this aspect. It is indeed crucial to consider viral contamination when performing RNA-seq experiments, especially when the percentage of mapped reads is unexpectedly low as was the case in some of our samples. We would like to highlight our previous publication: Verhoeven *et al.* 2023 (*New Phytologist*, doi: https://doi.org/10.1111/nph.18466) on this matter, where we studied the effects of ArLV1 on

plant transcriptome and physiology. Monitoring for potential pathogen sequences has indeed provided valuable insights, and we encourage the broader community to adopt similar analyses in their RNA-Seq analysis pipelines.

4) Figure 2 - The transcriptomic response of plants to drought was very minimal. Please refer to it in the text with more explanations.

\>> **Response:** We explain in the text: "*The relatively mild drought intensity likely explains the limited number of DEGs (Clauw et al., 2015). In addition, the young leaves that were sampled may be less responsive to drought than for instance roots tissues, as plants might evoke protective mechanisms to prioritize stress protection of younger leaves and meristematic tissues (Rankenberg et al., 2024, 2021; Verelst et al., 2013).*" (Line 440-444).

We have also added a remark to the Result section to emphasis the mild drought effect: "*This is likely due to the mild progressive drought used here. However, plants in our treatment did experience drought as evidenced by typical physiological responses such as an elongated primary root (Supplemental Fig. S5D), and reduced stomatal conductance (Fig. 1E).*" (Line 181-184).

5) Line 242 - Why not show the results of the GRN analysis in the manuscript (now only shown in supplemental material)? Also please add some text in this section about what/how the GRN was performed.

\>> **Response:** The candidate genes selected for functional validation in our study were derived from two main sources: (i) transcription factors (TFs) identified from up-regulated gene clusters (Figure 2G and 2H), and (ii) genes that exhibited the most interactions with other regulators within the constructed gene regulatory networks (GRNs) (Figure S19). Given that the majority of the validated candidates are TFs, with only a few derived directly from the GRNs, we chose to highlight the heatmaps of these identified TFs as a main figure, and present the GRNs in the supplementary material.

To construct the GRNs, we utilized the TF2Network tool, which allows for the identification of regulatory interactions between TFs and their target genes. We have now added this information to the both the Materials and Methods (Line 615-616 in the revised manuscript) and the Result (Line 241-243 in the revised manuscript) sections as requested.

6) Regarding mutant analysis, was this done with 2 independent alleles for each gene? I would like to see an analysis of 2 independent alleles at least for the few genes the authors mentioned in the abstract, but maybe the editor would like to see for more.

\>> **Response:** We initially sought to include additional mutant lines for functional validation. However, we encountered difficulties with some NASC lines, such as issues with germination or failure to obtain homozygous mutant lines. For example, in the case of *ATL80*, which we identified as a positive regulator under PSD conditions, we attempted to use both the SALK_046204 and SALK_088190 lines. Unfortunately, we were unable to secure homozygous lines for SALK_046204, so we proceeded with only the SALK_088190 line for our study.
To ensure the robustness of our results, we prioritized the use of mutant lines that have been validated in previous publications (as indicated in Table S6 and S7). Our primary objective was to explore genetic effects across a broad range of candidates (Table S5), rather than to dissect

the precise molecular mechanisms of specific genes at this stage. Future work will focus on unraveling the detailed roles of confirmed candidate genes in multi-stress tolerance.

7) There is no mention as to how the GRN was done in the method section. Please add more information on this.

>> **Response:** This comment is similar to point 3 of reviewer 1. A description of the methodology used for GRN construction has been added to the Materials and Methods section as requested (Line 614-615) and explanation has been also added to the Results section (Line 241-243).

8) Regarding the discussion, Zandalinas et al 2021 [Zandalinas SI, Sengupta S, Fritschi FB, Azad RK, Nechushtai R, Mittler R. (2021) The impact of multifactorial stress combination on plant growth and survival. New Phytol. 230(3):1034-1048.] used sublethal stress levels to study the response of Arabidopsis to multifactorial stress combination identifying similar additive effects. Please add this work to your discussion.

>> **Response:** We have incorporated a discussion of Zandalinas et al. (2021) in the Discussion section (lines 388-391) of the revised manuscript, acknowledging this important contribution to the understanding of multifactorial stress impacts and drawing parallels with our findings on additive negative effects under sublethal stress combinations. We now write:
*"Previous research has revealed that in general, multifactorial stress combinations of sublethal severity have negative impacts on plant growth and survival (Zandalinas et al., 2021b). This highlights the importance of studying plant acclimation to stress combinations at a relevant stress intensity."*

10-Oct-2024
Dr. Martijn Van Zanten
Universiteit Utrecht
Molecular Plant Physiology
Utrecht 3584 CH
Netherlands

MSID: PP2024-RA-00944R1
MS TITLE: Deciphering Arabidopsis thaliana Acclimation to Sublethal Combined and Sequential Abiotic Stresses

Dear Dr. Martijn Van Zanten:

I am pleased to inform you that your revised manuscript "Deciphering Arabidopsis thaliana Acclimation to Sublethal Combined and Sequential Abiotic Stresses" has been accepted for publication in Plant Physiology. Reviewer 1 has a couple of very minor suggestions, which you may want to adopt before the proofing stage. Your manuscript will be checked for consistency with journal formatting and image standards by one of our Science Editors, who will contact you shortly. They may have a number of queries and suggestions for you. Please keep in mind that they work to increase the online visibility of your article and its attraction for readers from the widest of backgrounds. You will have the opportunity to review any revisions, and I hope you will work constructively with them to get the most out of your research. You will also have the opportunity to review all changes when you receive the author proofs for your article.

Once validated for publication, your article will be sent to a Science Editor for a final review of your manuscript and source files. After your final files are uploaded, your article will appear in the next available issue and the online Preview version of your article will be posted after your signed Copyright and Checklist forms and final source files are received.

Please be sure to include the following in your manuscript file:
Harvard style citations
List of all author contributions and funding information at the beginning of manuscript after the titles and author list.
List all supplemental materials associated with the article, with titles, at the end of the ms, before the references and acknowledgments section.
Complete Figure Legends for all figures appearing in the article listed before the Literature Cited.

**LICENSE INFORMATION** Shortly after your paper is sent to production, you will receive an email from our publisher's author support team, **SciPris**, with information on article processing charges, as well as the types of Open Access license you would be able to purchase. The accepted version of your manuscript will not appear on our Advance Articles page until the license is selected and signed, so please look out for an email from SciPris. For more information see the Article Publication Fees section of our author guidelines: https://academic.oup.com/plcell/pages/General-Instructions

Finally, we encourage your submission of artwork for the journal cover. If there is an image or illustration related to your paper that you would like to have considered, please email it in .pdf or .tif format to clowe@aspb.org. An image title, brief, 2-3 sentence description of the image, and the name of the person credited for the image are also encouraged. If your image is selected to appear on the online cover of the journal, you will be contacted with the scheduled issue date and further information.

Thank you and congratulations on your Plant Physiology paper!

Sincerely,
Editor
Plant Physiology

=====
FIRST AUTHOR PROFILES
=====

We recognize the hard work that goes into being First Author of a Plant Physiology paper by publishing a "First Author Feature" for each First Author alongside your paper.

If the first author(s) would like to submit information for a feature on Facebook and Twitter, fill out the form by clicking on the link below:

Plant Physiology First Author Feature Form: https://forms.office.com/r/ZBCGZfhm8F
If you cannot access the form through the link above, please contact Rachel at rbelsky@aspb.org.

To upload an image of yourself to accompany your profile, please put your photo in the correct journal folder at the link below. You must label your image: Author First Name Last Name Article Title.

First Author Profile Image Dropbox: https://www.dropbox.com/scl/fo/xz029p7qjr2jml2rp2kmq/h?dl=0&rlkey=6fkxcaqmxx35ap152b2osy1fz

=====
IMPORTANT REMINDER: PEER REVIEW REPORTS
=====

If you opted to publish a peer review report along with your article during the original submission process, it will be prepared by the editorial staff and publicly posted with your manuscript, inside the zip file that contains any other supplemental material. As a reminder, the peer review report is a public record of all comments from editors and reviewers, as well as your prior responses, as you received them in the decision letters for each draft of your manuscript. If you agreed to publish this report and have changed your mind, or are not sure if you selected this option, please contact the editorial office as soon as possible before signing the license agreement from our publisher.

-------------------------------------------------------------------------- Reviewer comments:
Reviewer #1 (Comments for the Author):

This is a resubmission of a previous version of this manuscript which I reviewed earlier in 2024. I thank the authors for addressing my queries from the first submission. Whilst the manuscript is much improved, I have some minor comments that the authors might consider addressing.

Line 254: "had the same number of nodes in THE GRN,"
Line 305: "Under combined TREATMENT..."
Line 334: "all mutants and WTs revealed A strong..."

I have no further comments and once again applaud the authors for the immense amount of work which has been undertaken to produce this compelling work.

Reviewer #2 (Comments for the Author):

The authors answered all of my concerns.
